# SCAMP3-Driven Regulation of ERK1/2 and Autophagy Phosphoproteomics Signatures in Triple-Negative Breast Cancer

**DOI:** 10.3390/ijms26199577

**Published:** 2025-10-01

**Authors:** Beatriz M. Morales-Cabán, Yadira M. Cantres-Rosario, Eduardo L. Tosado-Rodríguez, Abiel Roche-Lima, Loyda M. Meléndez, Nawal M. Boukli, Ivette J. Suarez-Arroyo

**Affiliations:** 1 Department of Biochemistry, School of Medicine, Universidad Central del Caribe, Bayamón, PR 00956, USA; 419bmorales@uccaribe.edu; 2 Translational Proteomics Center, Comprehensive Cancer Center, University of Puerto Rico, Medical Sciences Campus, San Juan, PR 00921, USA; yadira.cantres@upr.edu (Y.M.C.-R.); loyda.melendez@upr.edu (L.M.M.); 3 Integrated Informatics Services Facility, University of Puerto Rico, Medical Sciences Campus, Río Piedras, PR 00936, USA; eduardo.tosado@upr.edu (E.L.T.-R.); abiel.roche@upr.edu (A.R.-L.); 4 School of Dental Medicine, Universidad Ana G. Méndez, Gurabo, PR 00778, USA; 5 Department of Microbiology, University of Puerto Rico, Medical Sciences Campus, San Juan, PR 00936, USA; 6 Department of Microbiology and Immunology, Universidad Central del Caribe, Bayamón, PR 00960, USA; nawal.boukli@uccaribe.edu

**Keywords:** SCAMP3, triple negative breast cancer, ERK1/2, autophagy, phosphoproteomics, mTOR

## Abstract

Extracellular signal-regulated kinase 1/2 (ERK1/2) inhibitors show therapeutic potential in triple-negative breast cancer (TNBC), but resistance through compensatory signaling limits their efficacy. We previously identified the secretory carrier membrane protein 3 (SCAMP3) as a regulator of TNBC progression and ERK1/2 activation. Here, we investigated the role of SCAMP3 in ERK1/2 signaling and therapeutic response using TMT-based LC-MS/MS phosphoproteomics of wild-type (WT) and *SCAMP3* knockout (SC3KO) SUM-149 cells under basal conditions, after epidermal growth factor (EGF) stimulation, and during ERK1/2 inhibition with MK-8353. A total of 4408 phosphosites were quantified, with 1093 significantly changed. SC3KO abolished residual ERK activity under MK-8353 and affected the compensatory activation of oncogenic pathways observed in WT cells. SC3KO reduced the phosphorylation of ERK feedback regulators RAF proto-oncogene serine/threonine-protein kinase Raf-1 (S43) and the dual-specificity mitogen-activated protein kinase kinase 2 (MEK2) (T394), affected other ERK targets, including nucleoporins, transcription factors, and metabolic enzymes triosephosphate isomerase (TPI1) (S21) and ATP-citrate lyase (ACLY) (S455). SCAMP3 loss also impaired the mammalian target of rapamycin complex I (mTORC1) signaling and disrupted autophagic flux, evidenced by elevated sequestosome-1 (SQSTM1/p62) and microtubule-associated protein light chain 3 (LC3B-II) with reduced levels of the autophagosome lysosome maturation marker, Rab7A. Beyond ERK substrates, SC3KO affected phosphorylation events mediated by other kinases. These findings position SCAMP3 as a central coordinator of ERK signaling and autophagy. Our results support SCAMP3 as a potential therapeutic target to enhance ERK1/2 inhibitor clinical efficacy and overcome adaptive resistance mechanisms in TNBC.

## 1. Introduction

Triple-negative breast cancer (TNBC) accounts for approximately 20% of all breast cancer cases and is defined by the absence of therapeutically targetable estrogen receptor (ER), progesterone receptor (PR), and human epidermal growth factor receptor 2 (HER2) expression. TNBC comprises distinct molecular subtypes, including Basal-Like 1 and 2 (BL1/BL2), Mesenchymal (M), Immunomodulatory, and Luminal Androgen Receptor (LAR), each with unique gene expression profiles and clinical outcomes. The pathogenesis across these subtypes involves the dysregulation of several signaling pathways central to tumorigenesis and progression into metastatic disease, such as epidermal growth factor receptor (EGFR), phosphatidylinositol 3-kinase/protein kinase B/mammalian target of rapamycin (PI3K/Akt/mTOR), janus kinase/signal transducer and activator of transcription (JAK/STAT), and mitogen-activated protein kinase (MAPK)/extracellular signal-regulated kinase 1/2 (ERK1/2) [1].

MAPK/ERK1/2 pathway is critical for cellular proliferation, differentiation, and survival. In many subtypes of TNBC, particularly the BSL, this pathway is constitutively active. This aberrant signaling is often initiated by the overexpression or hyperactivation of upstream receptor tyrosine kinases (RTKs), such EGFR, or through mutations in downstream effectors like rat sarcoma (RAS) or serine/threonine-protein kinase B-raf (BRAF) [1]. Consequently, preclinical and clinical efforts have focused on inhibitors of Dual specificity mitogen-activated protein kinase kinase (MEK) and ERK (e.g., trametinib, ulexertinib). However, their efficacy as monotherapies has been demonstrated to be limited. A primary reason is the rapid onset of adaptive resistance, where cancer cells dynamically rewire their signaling pathways to circumvent inhibition [2].

A critical cellular process intertwined with MAPK/ERK1/2 signaling and therapeutic resistance is autophagy. Autophagy plays a dual and context-dependent role in cancer. In early stages, it can suppress benign tumorigenesis by clearing damaged organelles and protein aggregates, thereby maintaining cellular homeostasis and genomic stability [3]. However, in established tumors subjected to metabolic stress or therapeutic environments, autophagy acts as a survival mechanism [4]. The ERK1/2 pathway is a key regulator of this process, capable of inducing autophagy through the phosphorylation of autophagy proteins [5,6]. However, the scaffolding proteins that coordinate ERK1/2 activity to the autophagic machinery remain poorly defined.

Emerging evidence points to secretory carrier membrane protein 3 (SCAMP3) as a potentially important regulator of oncogenic signaling and vesicle trafficking. SCAMPs is a family of integral membrane proteins functioning in endosomal sorting and vesicle trafficking between the trans-Golgi network, endosomes, and the plasma membrane [7]. SCAMP3 expression has been found elevated in multiple cancers, including hepatocellular carcinoma, glioma, and pancreatic cancer, where its high expression correlates with poor prognosis, cancer progression, and chemoresistance [8,9,10]. In our previous work, we established that SCAMP3 is a critical regulator of EGFR trafficking, thereby sustaining ERK1/2 activation and promoting proliferation and motility in TNBC cells [11]. Interestingly, this pro-oncogenic role appears to be context-dependent, as SCAMP3 can act as a tumor suppressor in lung adenocarcinoma by promoting EGFR degradation and attenuating ERK1/2 signaling [12]. While the MAPK/ERK is a central driver of TNBC, the network-level mechanisms that coordinate its signal transduction are poorly understood. Considering SCAMP3 as an ERK1/2 pathway regulator and its implication in modulating multiple oncogenic pathways highlights the need to define its specific molecular functions within TNBC [8,11,13]. Given that a drug’s therapeutic effect rarely results from engaging a single protein, but is rather the result of its influence on the entire biological network [14], a study focused on a systems-level view is necessary.

To address this gap, we employed isobaric label tandem mass tag (TMT)-based LC-MS/MS quantitative proteomics to comprehensively profile SCAMP3-dependent signaling networks in TNBC cells. By comparing wild-type and *SCAMP3*-knockout cells under basal, EGF-stimulated, and ERK1/2-inhibited conditions, we sought to achieve the following: (1) identify SCAMP3-regulated phosphorylation events, (2) characterize the feedback and compensatory signaling responses triggered by ERK1/2 inhibition, and (3) investigate the functional interplay between ERK1/2 activity and autophagy regulation.

Our findings reveal that SCAMP3 acts as a central hub in TNBC. It influences ERK-dependent phosphorylation of transcription factors, metabolic enzymes, and feedback regulators and controls autophagy. SCAMP3’s role as a regulator of phosphorylation events extends far beyond the MAPK cascade. Its loss alters the phosphorylation of canonical substrates for other key oncogenic kinases. These insights position SCAMP3 as a key co-regulator of oncogenic signaling and a potential new therapeutic target in TNBC.

## 2. Results

### 2.1. SCAMP3 Knockout Enhances ERK1/2 Inhibition Effects

**Figure 1:**
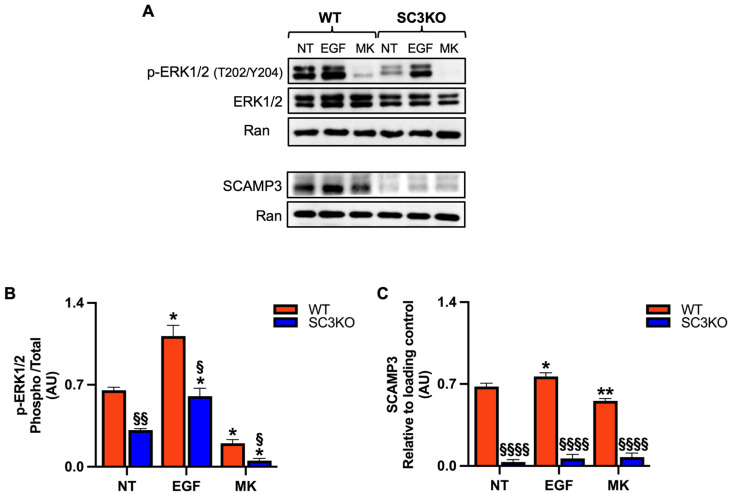
Loss of SCAMP3 enhances the effects of MK-8353 (**A**) Representative immunoblot of whole-cell lysates from wild-type (WT) and SCAMP3-knockout (SC3KO) SUM-149 cells. Cells were left untreated (NT), stimulated with EGF (10 ng/mL, 30 min), or treated with MK-8353 (MK) (8 µM, 2 h) after EGF stimulation. Blots were probed for SCAMP3, total ERK1/2, and phosphorylated ERK1 (Thr202/Tyr204, 44 kDa) and ERK2 (Thr185/Tyr187, 42 kDa). p-ERK1/2 antibody detects phosphorylation at Thr202/Tyr204 of ERK1 and the corresponding Thr185/Tyr187 sites of ERK2. Ran served as a loading control. (**B**) Densitometric quantification of p-ERK1/2 relative to total ERK1/2, and (**C**) SCAMP3 levels normalized to Ran, from three independent experiments. Data are presented as mean ± SEM (*n* = 3). Statistical significance was determined using two-way ANOVA with Tukey’s post hoc test: (*) vs. untreated (NT) control within each genotype (* *p* ≤ 0.05, ** *p* < 0.01), and (^§^) WT vs. SC3KO under the same condition (^§^ *p* ≤ 0.05, ^§§^ *p* < 0.01, ^§§§§^ *p* < 0.0001).

To investigate the role of the secretory carrier membrane protein 3 (SCAMP3) in Triple-negative breast cancer (TNBC) and its contribution to the extracellular signal-regulated kinase 1/2 (ERK1/2) signaling, we used *SCAMP3*-knockout (SC3KO) SUM-149 cells previously generated in our laboratory [11]. Wild-type (WT) and SC3KO cells were treated with 8 μM MK-8353 for 2 h or EGF (10 ng/mL) for 30 min. As shown in Figure 1A,B, SC3KO reduced basal p-ERK1/2 levels, confirming our previous findings [11]. Treatment with MK-8353 showed a difference in inhibitor sensitivity, while a residual p-ERK2 signal was detectable in WT cells, and the combination of SC3KO and MK-8353 treatment led to the complete abolishment of pERK-1/2. MK-8353 also decreased SCAMP3 levels (Figure 1A,C), whereas EGF upregulated SCAMP3, suggesting a potential feedback loop.

### 2.2. Phosphoproteomics Profiling Reveals SCAMP3-Dependent Signaling Networks

**Figure 2:**
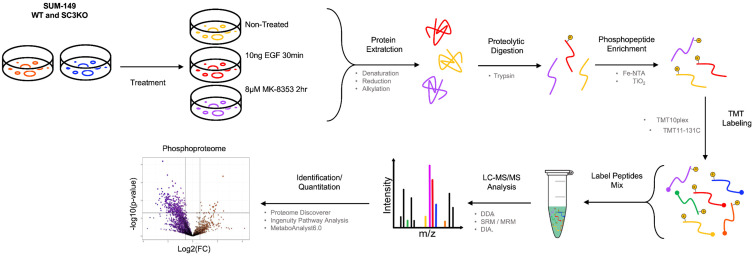
Schematic overview for TMT-based quantitative phosphoproteomics analysis. The schematic illustrates the TMT isobaric labeling-based quantitative phosphoproteomics analysis of wild-type (WT) and SCAMP3-knockout (SC3KO) SUM-149 cells under three conditions: untreated (NT), epidermal growth factor (EGF) stimulation (10 ng/mL, 30 min), or MK-8353 (8 μM, 2 h). Proteins from total lysates were digested into peptides and subjected to phosphopeptide enrichment using immobilized metal affinity chromatography (IMAC). Peptides from all conditions were labeled with unique isobaric tandem mass tags (TMTs) (Table S1). All labeled samples were pooled and analyzed using high-resolution liquid chromatography-tandem mass spectrometry (LC-MS/MS). The workflow was performed on four (*n* = 4) independent biological replicates.

To define SCAMP3/ERK1/2 regulated signaling with quantitative proteomics, we used TMT-based LC-MS/MS phosphoproteomics in wild-type (WT) and *SCAMP3* knockout (SC3KO) cells under the same conditions used before (untreated, EGF (10 ng/mL, 30 min), MK-8353 (8 µM, 2 h). The schematic of the TMT-based phosphoproteomics is described in Figure 2. From this approach, we identified 4408 unique phosphosites. We focused on phosphopeptides whose quantity is significantly affected by treatment conditions. We defined a regulated phosphopeptide as one with a log_2_-fold change of ≥1.5, with a *p*-value of ≤0.05. From this analysis, we observed the regulation of 1093 phosphosites corresponding to 689 proteins (Table S1).

**Figure 3:**
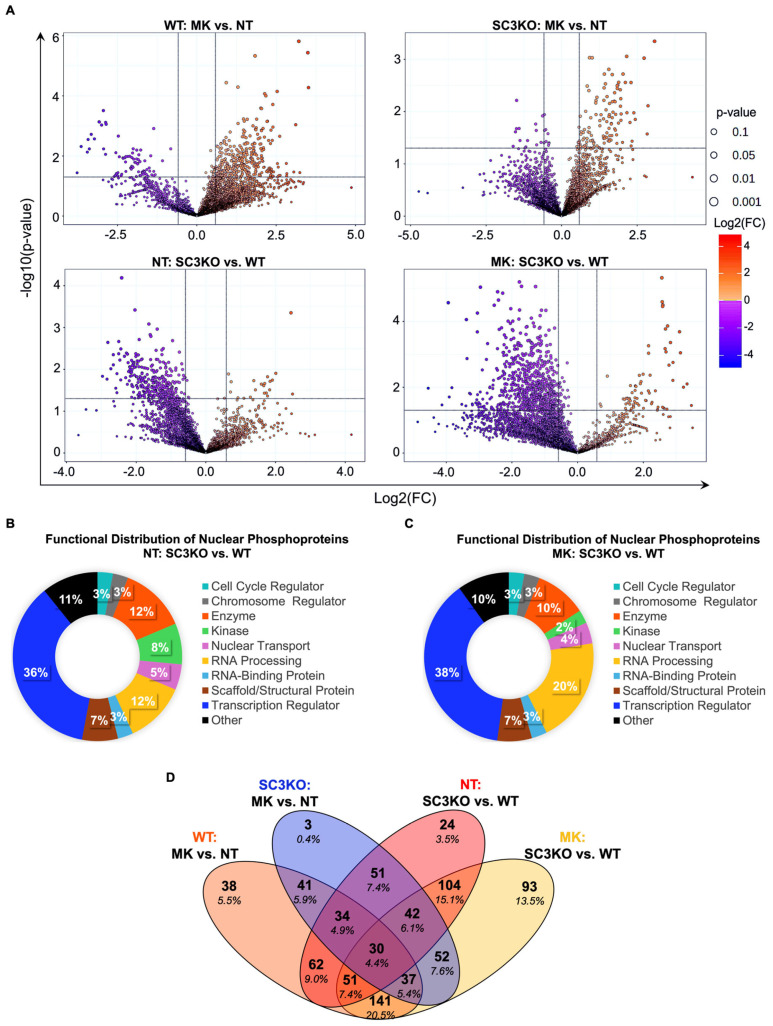
Phosphoproteomics profile of SUM-149 WT and SC3KO cells. (**A**) Volcano plots showing differentially phosphorylated sites for four comparisons: (**A**) upper-left panel: wild-type (WT) cells treated with MK-8353 vs. untreated (NT) (WT: MK vs. NT); upper-right panel: SCAMP3-knockout (SC3KO) cells treated with MK-8353 vs. NT (SC3KO: MK vs. NT); lower-left panel: untreated cells, comparing SC3KO vs. WT (NT: SC3KO vs. WT); lower-right panel: MK-8353-treated cells, comparing SC3KO vs. WT (MK: SC3KO vs. WT). Volcano plot shows the effects on phosphorylated sites expression analyzed at −1.5 ≥ 1.5 log_2_-fold change (vertical black lines). Down-regulated phosphosites are to the left of the vertical black line while up-regulated phosphosites are to the right. Phosphosites with significant changes (*p* ≤ 0.05) are plotted above the horizontal line. (**B**,**C**) Functional analysis of regulated differentially phosphorylated nuclear proteins in (**B**) untreated and (**C**) MK-8353-treated conditions. (**D**) Venn diagram illustrating the overlap of significantly regulated phosphoproteins among the different comparisons. Statistical analysis was performed using MetaboAnalyst 6.0 with a significance threshold of *p* ≤ 0.05 and a Log_2_ fold change (FC) ≥ 1.5. Functional analysis was performed using Ingenuity Pathway Analysis (IPA) software version 24.0.1.

In MK-8353-treated WT cells compared with untreated control (WT: MK vs. NT), 216 phosphosites were upregulated and 96 were downregulated (Figure 3A). In SC3KO cells, MK-8353 induced the upregulation of 77 phosphosites and the downregulation of 7 phosphosites (SC3KO: MK vs. NT). Comparative analysis between SC3KO and WT cells under basal conditions (NT: SC3KO vs. WT) identified 17 upregulated and 186 downregulated phosphosites. MK-8353-treated SC3KO cells showed 55 upregulated and 439 downregulated phosphosites relative to MK-treated WT cells (MK: SC3KO vs. WT). Most deregulated phosphorylated proteins and phosphosites localized to the nucleus (Figure S1), with 48% of them classified as transcription regulators and enzymes (Figure 3B,C). A conserved subset of 30 phosphosites was shared across all comparisons (Figure 3D).

**Figure 4:**
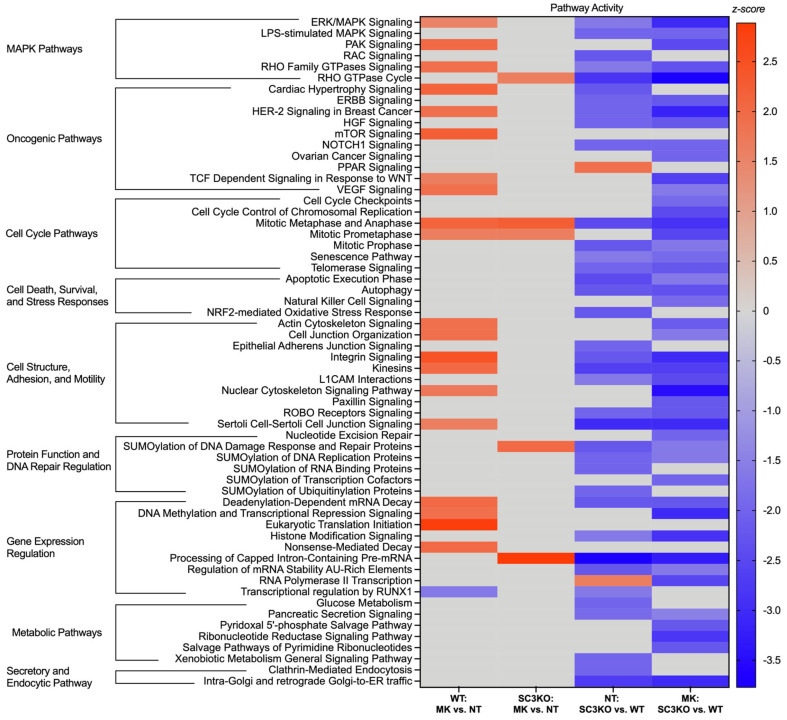
SCAMP3 loss reverses the pathway response to ERK inhibition. The heatmap displays the predicted activity state of key canonical pathways across the four comparisons. The color intensity corresponds to the activation z-score. Red bars indicate predicted pathway activation (z-score ≥ 1.5), blue indicates predicted pathway inhibition (z-score ≤ −1.5), and gray indicates that no significant activity pattern was predicted. For this analysis, only statistically significant pathways (*p* ≤ 0.05) with at least five proteins showing directionally consistent changes in phosphorylation were considered. Canonical pathway analysis was performed using Ingenuity Pathway Analysis (IPA) software version 24.0.1.

Next, we performed bioinformatic analyses of regulated phosphoproteins to gain a global view of signaling pathways regulated by SCAMP3 and ERK1/2. We focused the analysis on pathways with a z-score of ≥1.5 to predict changes in activity (Figure 4). The analysis showed divergence in cellular response to ERK inhibition. In WT cells, MK-8353 triggered a broad range of compensatory activation of oncogenic pathways (mTOR signaling), cell cycle pathways, eukaryotic translation initiation, and other MAPK pathways [2,4,15,16].

In contrast, in SC3KO cells, the same pathways were predicted to be inhibited upon MK-8353 treatment. Cell death and survival pathways, such as autophagy and the apoptotic execution phase, and metabolic pathways were also predicted to be inactivated. This opposing response suggests that SCAMP3 is a critical mediator of signaling rewiring in cancer cells after ERK1/2 pathway inhibition.

The knockout of SCAMP3 modulated the activation of other canonical pathways independently of ERK1/2. Peroxisome proliferator-activated receptor (PPAR) signaling and RNA Polymerase II transcription are predicted to be activated in NT: SC3KO vs. WT group. As expected, secretory and endocytic pathways (clathrin-mediated endocytosis and Golgi trafficking) were identified with low activity in SC3KO cells (Figures S5 and S6).

### 2.3. SCAMP3 Knockout Leads to Inhibition of ERK1/2 Pathway

After observing that the knockout of SCAMP3 inactivated the canonical MAPK/ERK pathway, we examined which upstream and downstream ERK substrates were affected. We identified 42 deregulated phosphosites in the NT: SC3KO vs. WT comparison recognized as ERK targets, as referenced in “A Compendium of ERK1/2 Targets” and the PhosphoSitePlus^®^ dataset (Table 1, Tables S2 and S3) [17].

Consistent with the results shown in Figure 1, phosphoproteomics analysis showed that SCAMP3 knockout reduced p-ERK2 at T185 (−1.2). Although this change did not meet the pre-established cutoff, T185 is critical for ERK2 autophosphorylation [18]. Since MEK1/2-mediated phosphorylation of ERK1/2 at T185 and Y187 is essential for full activation, perturbations at these sites could impair ERK complex assembly and downstream signaling fidelity [19]. ERK1/2 signaling is modulated by negative feedback loops, which involve ERK1/2-dependent phosphorylation of upstream components and adaptor proteins, forming a self-limiting circuit that regulates signal amplitude and duration [19]. One central feedback mechanism includes the phosphorylation of Raf-1 at six regulatory sites across its N-terminus (S29/S43), flexible hinge (S259/S296/S301), and C-terminus (S642). These modifications hinder Raf-1 translocation to the plasma membrane and Ras binding, reducing MAPK/ERK1/2 activity [20]. In our dataset, we identified SC3KO decreased p-Raf-1 at the inhibitory site, S43 (−1.6). and this effect was even more pronounced upon ERK inhibition with MK-8353 (−2.0).

ERK1/2 can also phosphorylate MEK with a negative outcome. ERK1/2 phosphorylates MEK1 at T292/T386 and MEK2 at T394, disrupting MEK heterodimer formation and limiting kinase activity [19,21]. In WT cells, MK-8353 upregulated MEK2 T394 (+2.6), while in SC3KO cells, this phosphosite is downregulated (−2.2). Most of this effect could be attributed to the loss of SCAMP3, since in SC3KO, the phosphorylation of MEK2 at T394 decreased (−1.9).

ERK1/2 phosphorylates phenylalanine-glycine (FG)-repeat domains of nucleoporins, modulating their interaction with nuclear transport receptors. Nuclear pore complexes Nup153 and NUP214, and the translocated promoter region protein (TPR) have previously been identified as substrates of ERK1/2 [22]. SC3KO increased the phosphorylation of the nuclear basket-associated protein, NUP153 at S334 (+2.0), a site directly phosphorylated by ERK1/2 [22], and decreased S209 (−2.0). MK-8353 decreased S614 (−2.0) and increased S334 (+1.5) when compared with treated WT cells. ERK1/2 also phosphorylates the cytoplasmic nucleoporin Nup214 at S1710 and S1809, affecting its binding to the nuclear import receptor, importin-β [22]. In our data, MK-8353 downregulated Nup214 S646 (−2.1) but increased phosphorylation at S433 and T434 (+1.9). Interestingly, SC3KO reversed these effects, decreasing S433/T434 phosphorylation (−2.0). SC3KO also decreased site S940 (−3.2). Upon reaching the nuclear pore, ERK is tethered by the nucleoporin TPR, which acts as a scaffold protein. This anchoring facilitates the subsequent phosphorylation of TPR and NUP153 by ERK [23]. TPR is typically downregulated under ERK suppression [24]. In our data, MK-8353 increased S2155 (+2.6), while SC3KO had the opposite effect.

As shown in Figure 3, SC3KO modulates phosphorylation of transcription factors, particularly members of the E74-like ETS transcription factor (ELF) subfamily [25]. In WT cells, the inhibition of ERK1/2 increased the phosphorylation of ELF1 at S187 (+1.5), but when SCAMP3 is absent, this effect was reversed (−2.5). SC3KO also decreased the phosphorylation of ELF4 at S188 (−1.8). Although the phosphorylation of ELF1 S187 has been linked to EGF signaling, the functional role of ELF4 S188 remains unknown. The activity of ERK1/2 after treatment can be explained by the residual p-ERK2 observed in Figure 1.

### 2.4. SCAMP3 Regulates Pseudokinases and Other Kinase Substrates

Loss of SCAMP3 influenced phosphorylation events beyond ERK-regulated sites. To explore SCAMP3’s broader role in kinase signaling, we performed in silico kinase enrichment analysis using K3 Kinase Enrichment Analysis (KEA3) software, version 3.0 (Figures S2–S4, Table S4). Among hypophosphorylated proteins in SC3KO vs. WT cells, the top five enriched kinases were casein kinase 2 alpha, subunits 1 and 2 (CK2alpha1 and alpha2), cyclin-dependent kinases 2 and 9 (CDK2 and 9), and the extracellular signal-regulated kinase 2 (ERK2). ERK2, as expected, emerged as a key regulator, with computational predictions linking it to 81 substrates.

Table 2 details phosphosites differentially regulated by SC3KO and their predicted upstream kinases. PEAK1-related kinase-activating pseudokinase 1 (*PRAG1*) (encoding pseudopodium enriched atypical kinase (PEAK1)-related kinase-activating pseudokinase 1 (PEAK2) is a catalytically inactive tyrosine-protein kinase that assembles complexes that influence oncogenic processes [26,27]. In colorectal cancer, the tyrosine-protein kinase Abelson tyrosine-protein kinase (Abl)-mediated phosphorylation of PEAK2 at Y413 enhances Abl activation and tumor progression [27]. PEAK2 also interacts with C-terminal Src kinase (CSK), a negative regulator of the Src family kinases (SFKs), facilitating phosphorylation at Y238, Y343, and Y391 to promote cell motility [28,29]. Additionally, the pre-mRNA processing factor kinase (PRP4K) is linked to phosphorylation of PEAK2 at S745, though its functional role remains unclear [30]. In our data, SC3KO reduced phosphorylation of PEAK2 at this site (−2.1), suggesting SCAMP3 may regulate PRP4K.

*SCAMP3* knockout also affected the phosphorylation of casein kinase 2 (CK2) substrate, calnexin. Calnexin is an endoplasmic reticulum (ER)-resident chaperone involved in quality control. Its phosphorylation at the C-terminal drives calnexin’s redistribution within ER membranes and contributes to cellular remodeling [31]. While ERK’s direct role in calnexin phosphorylation is unconfirmed, MK-8353 treatment reduced phosphorylation at S554 (−2.1). CK2 phosphorylates calnexin at S554 and S564, modulating its interaction with the ER retention factor phosphofurin acidic cluster sorting protein 2 (PACS-2) and promoting its redistribution to the plasma membrane [32]. SC3KO decreased the phosphorylation of calnexin at S554 (−2.0), whereas MK-8353 reversed this effect (+3.5), suggesting that SCAMP3 likely modulates ERK1/2 and CK2-dependent signaling pathways to regulate calnexin dynamics.

### 2.5. SCAMP3 Influences Metabolic Processes in TNBC

We identified three key metabolic enzymes as substrates of the enriched kinases: ATP-citrate lyase (ACLY), triosephosphate isomerase (TPI1), and choline phosphate cytidylyltransferase A (PCYT1A). TPI1 catalyzes the isomerization of glyceraldehyde 3-phosphate and dihydroxyacetone phosphate, an essential reaction for maintaining glycolytic flux [33]. Our data showed that SC3KO significantly decreases TPI1 phosphorylation at S21 (−2.9) (Figures S5 and S6). The phosphorylation of TPI1 in S21 by protein kinase A (PKA) enhances its enzymatic activity and supports cancer growth [34]. Cyclin-dependent kinase 2 (CDK2) also targets the same site during drug-induced apoptosis and oncogenesis [35,36].

ACLY catalyzes the ATP-dependent conversion of citrate and coenzyme A (CoA) to oxaloacetate and acetyl-CoA, linking glycolysis to lipid biosynthesis by supplying acetyl-CoA for fatty acid synthesis, cholesterol production, and histone acetylation [37]. The activity of ACLY is regulated by Akt1 via phosphorylation at S455 in a mTORC2-dependent manner [38,39]. SC3KO reduced ACLY phosphorylation at S455 (−1.8). This finding implicates a regulation of Akt by SCAMP3, which is consistent with our previous work [11].

PCYT1A/choline-phosphate cytidylyltransferase A (CCT-α) is the rate-limiting enzyme in the phosphatidylcholine (PC) biosynthesis pathway, with its activity and subcellular localization modulated through reversible phosphorylation [40,41]. ERK1/2-mediated phosphorylation at S315 suppresses enzymatic function under activating conditions, whereas rapid dephosphorylation at S315 combined with sustained phosphorylation at S362 and Y359 facilitates translocation to the nuclear envelope [42,43]. PCYT1A/CCT-α is also phosphorylated at S362 by casein kinase 2 (CK2). When the PCYT1A/CCT-α is phosphorylated at this site by CK2, the enzyme detaches from the membranes and becomes inactive [44]. In our study, MK-8353 treatment or the knockout of SCAMP3 increased the phosphorylation of PCYT1A/CCT-α at S362 (+4.0; +2.5). Interestingly, ERK1/2 inhibition in SC3KO cells reduced S362 phosphorylation, indicating an intricate regulation mechanism between SCAMP3, ERK, and CK2. Collectively, these results position SCAMP3 as a potential regulator of metabolism reprogramming in cancer.

### 2.6. SCAMP3 Modulates the Phosphorylation of Cell Surface Receptors

Given that SCAMP3 regulates EGFR, we hypothesized that it may also influence the phosphorylation of other cell surface receptors. In comparison with WT cells, SC3KO decreased phosphorylation of the receptor cluster of differentiation 44 (CD44) at S697 (−1.8). This site has been functionally linked to cell motility upon phorbol ester treatment and associated with lysophosphatidic acid (LPA) signaling [45,46]. As a validated phospho-S697 antibody is not commercially available, we validated our results by immunoblotting for total CD44 (Figure S7). CD44 basal expression was similar between genotypes. However, following EGF stimulation or MK-8353 treatment, CD44 levels were significantly reduced in SC3KO cells compared with WT, suggesting that SCAMP3/EGFR contributes to maintaining CD44 abundance.

Furthermore, SCAMP3 loss altered the phosphorylation dynamics of the Roundabout receptor (ROBO1), which is implicated in axon guidance, tumorigenesis, and metastasis [47,48]. Although ROBO1’s serine phosphorylation sites are less well characterized than its tyrosine residues, phosphorylation at S940 has been linked to enhanced invasiveness in lung adenocarcinoma [49]. Notably, SC3KO reduced the phosphorylation of ROBO1 at S1055 (−2.7), whereas it increased the phosphorylation at S940 (+2.2). These findings suggest that SCAMP3 modulates not only receptor tyrosine kinase (RTK) signaling but also non-RTK receptors, potentially influencing cellular responses to extracellular signals. Future mechanistic experiments should focus on determining whether SCAMP3 loss modulates their downstream signaling in response to their ligands.

### 2.7. SCAMP3 Loss Disrupts Autophagic Flux

**Figure 5:**
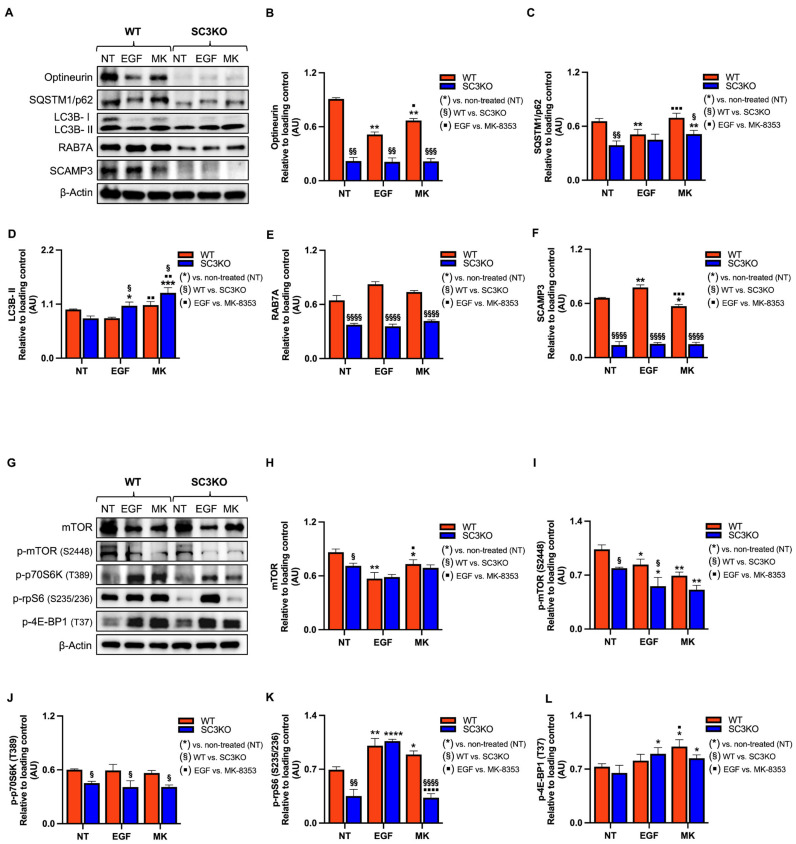
SCAMP3 loss combined with MK-8353 disrupts autophagic flux and mTOR signaling. Representative immunoblot of whole-cell lysates from wild-type (WT) and SCAMP3-knockout (SC3KO) SUM-149 cells. Cells were either left untreated (NT), stimulated with EGF (10 ng/mL EGF, 30 min), or treated with MK-8353 (MK) (8 µM, 2 h). (**A**) Blots probed for autophagy markers: optineurin, SQSTM1/p62, LC3B, RAB7A, and SCAMP3. β-actin served as a loading control. (**B**–**F**) Densitometric quantification of protein levels normalized to β-actin from three independent experiments. (**G**) Blots probed for mTOR, p-mTOR (2448), p-p70S6K (T389), p-rpS6 (S235/236), and p-4EBP1 (T37). β-actin served as a loading control. (**H**–**L**) Densitometric quantification of phosphorylated and total protein levels normalized to β-actin from three independent experiments. Data are shown as mean ± SEM (*n* = 3). Statistical significance was determined using two-way ANOVA with Tukey’s post hoc test: (*) vs. untreated (NT) control within each genotype (* *p* ≤ 0.05, ** *p* < 0.01, *** *p* < 0.001, **** *p* < 0.0001); (^§^) WT vs. SC3KO under the same condition (^§^ *p* ≤ 0.05, ^§§^ *p* < 0.01, ^§§§^ *p* < 0.001, ^§§§§^ *p* < 0.0001); (▪) EGF vs. MK-8353 treatment (▪ *p* ≤ 0.05, ▪▪ *p* ≤ 0.01, ▪▪▪ *p* ≤ 0.001 and ▪▪▪▪ *p* < 0.0001).

Building on our IPA, which predicted an inactivation of the autophagy pathway (Figure 4), and the previously reported role of SCAMP3 in this process [13], we next sought to determine the functional consequences of SCAMP3 loss by examining key autophagy markers and the upstream regulator, mTOR. Under basal conditions, SC3KO decreased phosphorylation of the autophagy cargo receptors sequestosome-1 (SQSTM1/p62) and optineurin (OPTN) at S272 (−1.5) and S526 (−1.5), respectively. Upon ERK1/2 inhibition, phosphorylation of SQSTM1/p62 increased in SC3KO cells, whereas changes in p-OPTN remained SCAMP3-dependent. As SQSTM1/p62 and OPTN are primarily regulated via degradation, we examined their total protein levels using Western blot. To evaluate autophagic activity, we assessed autophagosome flux by LC3B-II and Ras-related protein RAB7A (Figure 5).

Western blot analysis of untreated cells showed that SC3KO reduced protein levels of the autophagy receptors OPTN and SQSTM1/p62, as well as RAB7A, a late endosomal GTPase required for autophagosome lysosome fusion and regulated by the GTPase activating protein, TBC1 domain family member 5 (TBC1D5) (Figure 5A–C,E) [50,51,52]. Consistently, results showed a reduction in the phosphorylation of m-TOR and its downstream targets phospho-ribosomal protein S6 kinase (p-p70S6K) and phospho- small ribosomal subunit protein S6 (p-rpS6) relative to WT cells (Figure 5G–K).

In WT cells, treatment with MK-8353 increased the phosphorylation of rpS6, validating our phosphoproteomics data (S236; +1.6) (Tables S2 and S3), and the eukaryotic translation initiation factor 4E-binding protein 1 (4EBP1), suggesting a compensatory feedback loop (Figure 5G,K,L). Contrary results were observed in SC3KO cells. The inhibition of ERK1/2 increased the levels of SQSTM1/p62 and of LC3B-II, an autophagosome accumulation marker [53], suggesting impaired autophagic flux (Figure 5A,D). RAB7A was downregulated in SC3KO cells under all conditions, pointing to defects in autophagosome maturation (Figure 5A,E).

**Figure 6:**
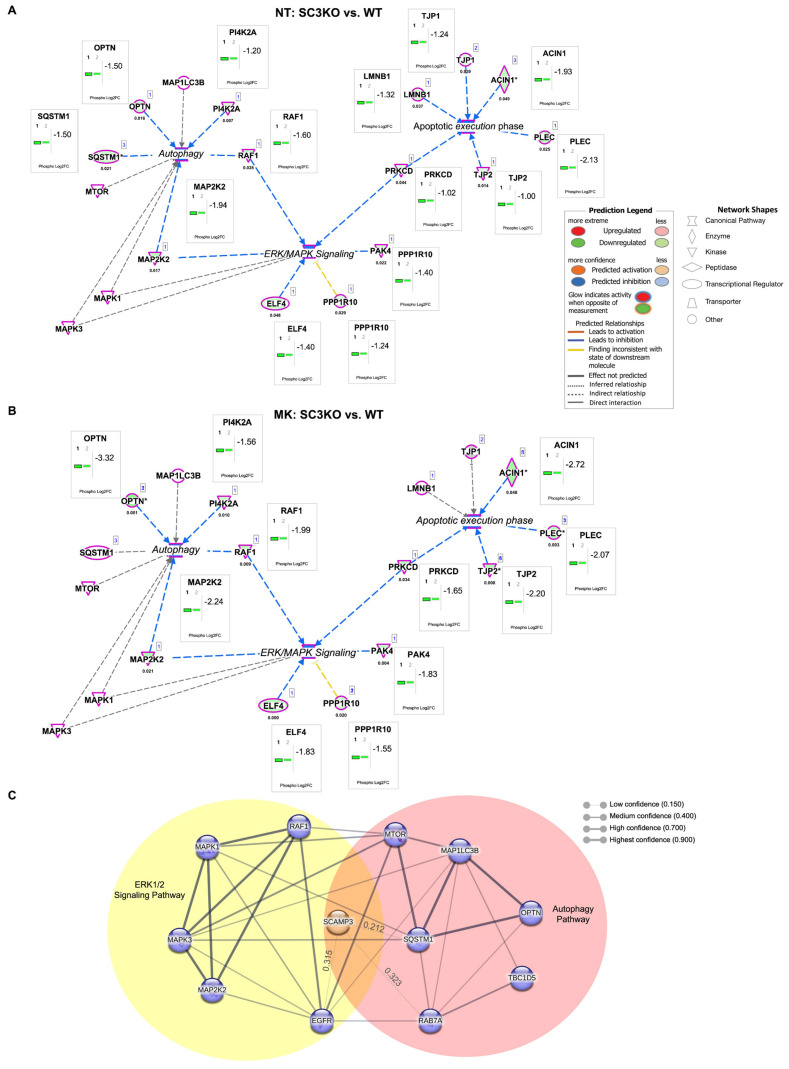
Molecular network analysis of the ERK/MAPK pathway and associated canonical pathways. Integrated molecular network of ERK/MAPK signaling, autophagy, and apoptotic execution pathways in non-treated (**A**) NT (SC3KO vs. WT) and MK-8353 treated groups (**B**) (MK: SC3KO vs. WT). Molecular network analysis was performed using Ingenuity Pathway Analysis (IPA^®^). Node color indicates relative expression (red: upregulated; green: downregulated), and edges represent curated molecular relationships from the IPA knowledgebase. Legend indicates the function of each protein and the interactions between them. Significant proteins are determined by Log_2_FC ≤ 1.5 and *p* ≤ 0.05. (**C**) Protein–protein interaction network of SCAMP3-regulated phosphoproteins within the ERK1/2 cascade (yellow) and autophagy (red) (NT: SC3KO vs. WT). The network was generated using the STRING 2024 database with a confidence cutoff of 0.400. Interactions with scores < 0.400 are included and labeled with their numerical confidence values. Predicted interactions with SCAMP3, EGFR, MAP1LC3B, and mTOR are also highlighted. Gene names displayed in the network correspond to the following proteins: *ACIN1*, apoptotic chromatin condensation inducer in the nucleus; *ELF4*, E74 like ETS transcription factor 4; *LMNB1*, Lamin-B1; *MAP1LC3B*, microtubule-associated protein 1 light chain 3 beta; *MAP2K2*, dual specificity mitogen-activated protein kinase kinase 2; *MAPK1*, mitogen-activated protein kinase 1; *MAPK3*, MAP kinase-activated protein kinase 3; *mTOR*, mammalian target of rapamycin; *OPTN*, optineurin; *PAK4*, serine/threonine-protein kinase PAK 4; *PI4K2A*, phosphatidylinositol 4-kinase type 2-alpha; *PLEC*, Plectin; *PPP1R10*, serine/threonine-protein phosphatase 1 regulatory subunit 10; *PRKCD*, Protein kinase C delta type; *RAF1*, RAF proto-oncogene serine/threonine-protein kinase: *SQSTM1*, Sequestosome-1; *TBC1D5*, TBC1 domain family member 5; *TJP1*, tight junction protein ZO-1, and *TJP2*, tight junction protein ZO-2. In IPA networks, an asterisk (*) next to a protein name means the node is representative for a group of molecules/isoforms/aliases, not just a single, unique protein.

IPA analyses were built with networks for MAPK/ERK, autophagy, and apoptotic execution. In untreated cells, the molecular network analysis showed that SC3KO cells exhibit coordinated downregulation of key MAPK/ERK components (MAP2K2/MEK2 and RAF-1) together with autophagy regulators (SQSTM1 and OPTN). These alterations overlap with changes in apoptotic execution proteins (Figure 6A). Upon ERK inhibition, autophagy-related proteins remained dysregulated, consistent with defective autophagy (Figure 6B). In this group dataset, protein kinase C delta type (PRKCD) was identified as the linker between the MAPK/ERK pathway and apoptotic execution signaling. Under treatment conditions, the autophosphorylation site (S304) of PRKCD was downregulated by 1.6 FC [54]. It is important to highlight that the knockout of *SCAMP3* decreased S304, but the regulation did not meet the criteria of Log_2_FC ≤ 1.5.

To map the functional connections of SCAMP3, we performed a protein–protein interaction analysis using the STRING database, applying a medium confidence threshold of 0.400. Given the limited literature on SCAMP3, interactions below this threshold were also included. The resulting network positioned SCAMP3 at the intersection of the ERK1/2 signaling cascade and autophagy pathways (Figure 6C). Collectively, these findings indicate that SCAMP3 coordinates crosstalk between ERK1/2 signaling and autophagy, and its loss compromises autophagic flux, particularly under ERK pathway inhibition.

**Figure 7:**
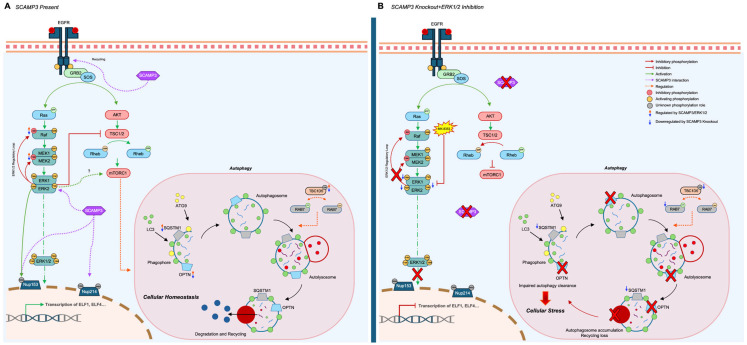
Proposed model: SCAMP3 coordinates ERK1/2 signaling and autophagy in TNBC. (**A**) Wild-type cells: Upon EGFR stimulation, SCAMP3 supports ERK1/2 activation, promoting phosphorylation of downstream substrates and enabling recruitment of LC3B and RAB7A. This facilitates autophagosome maturation and fusion with lysosomes, maintaining efficient autophagic flux. (**B**) SC3KO cells: Loss of SCAMP3 impairs ERK activation, reduces phosphorylation of key regulators, and decreases RAB7A. These changes lead to autophagosome accumulation and blocked autophagic clearance. ERK inhibition with MK-8353 further potentiates these effects. Names displayed in the figure correspond to the following proteins: AKT, protein kinase B; *ATG9*, autophagy-related 9A; *ELF1*, E74 Like ETS Transcription Factor 1; *ELF4*, E74 Like ETS Transcription Factor 4, ERK1/2, extracellular signal-regulated kinase 1/2; GRB2, growth factor receptor bound protein 2; LC3, microtubule associated protein 1 light chain 3; MEK1/2, mitogen-activated protein kinases; *NUP153*, nucleoporin 153; *NUP214*, nucleoporin 214; *OPTN*, optineurin; *RAB7*, Ras-related protein RAB7A; *RAF*, RAF proto-oncogene serine/threonine-protein kinase: *RAS*, rat sarcoma; SCAMP3, secretory carrier associated protein 3; *SOS*, SOS Ras/Rac Guanine Nucleotide Exchange Factor 1; *SQSTM1*, Sequestosome-1, *TBC1D5*, TBC1 domain family member 5; TSC1/2, tuberous sclerosis.

We propose that in WT cells (Figure 7A), SCAMP3 promotes effective signal transduction through the ERK pathway following EGF stimulation, thereby enabling the efficient phosphorylation of cytoplasmic and nuclear targets, including autophagy regulators. This phosphorylation cascade facilitates the recruitment and activation of RAB7A and LC3B, supporting functional autophagy. In SC3KO cells, disruption of ERK1/2 (Figure 7B) promotes an attenuated phosphorylation of downstream effectors, including mTOR. These events trigger the onset of autophagy but reduce levels of RAB7A, resulting in the accumulation of immature autophagosomes and impaired autophagic flux. Treatment with MK-8353 enhances this autophagic defect (Figure 6B).

## 3. Discussion

The dynamic regulation of the extracellular signal-regulated kinase 1/2 (ERK1/2) signaling is key for sustaining oncogenic processes in triple-negative breast cancer (TNBC). Using an integrated approach that combined ERK1/2 pharmacologic inhibition, quantitative phosphoproteomics, and functional assays, we identified SCAMP3 as a regulator of ERK1/2 phosphorylation dynamics with potential roles in downstream signaling amplification, feedback control, and autophagy regulation. Secretory carrier membrane protein 3 (SCAMP3) loss impaired ERK1/2 activation, altered phosphorylation of oncogenic signaling components, and disrupted autophagic flux, pointing to a scaffolding role for SCAMP3 in maintaining ERK1/2-driven tumor cell plasticity.

Although ERK1 and ERK2 share ~85% sequence similarity and regulate overlapping substrates, they play non-redundant roles in cancer [55]. In TNBC, ERK2 knockdown more effectively suppresses metastasis and cancer stemness compared with ERK1 silencing [56]. Consistent with this, our data show that ERK1 is more responsive to MK-8353, while SCAMP3 knockout completely abrogated residual p-ERK2 levels. Interestingly, MK-8353 also decreased SCAMP3 protein levels, suggesting reciprocal regulation. These findings raise the possibility that SCAMP3 stabilizes ERK2-specific signaling complexes and may shape therapeutic responses to ERK1/2 inhibitors.

The MAPK/ERK1/2 pathway is tightly regulated by feedback phosphorylation loops that prevent uncontrolled activation [19]. ERK1/2 phosphorylates RAF1 and MEK1/2 at inhibitory sites to maintain signaling homeostasis [57]. Our phosphoproteomics data show that SCAMP3 loss decreased phosphorylation at Raf-1 S43 and MEK2 T394, suggesting a potential disruption of these feedback mechanisms. SCAMP3 may contribute to maintaining feedback fidelity by organizing signaling complexes or facilitating spatial compartmentalization, similar to the role of scaffold proteins, kinase suppressor of Ras 1 (KSR1) and IQ motif containing GTPase activating protein 1 (IQGAP1) [19].

ERK1/2-driven transcriptional reprogramming depends on nuclear translocation, which is regulated in part by phosphorylation of nucleoporins. ERK1/2 targets FG-repeat nucleoporins such as the nuclear pore complex protein 153 (NUP153) and the nuclear pore complex protein 214 (NUP214) to modulate nuclear pore complex dynamics and selective import [22,24]. We found that SCAMP3 depletion reduced the phosphorylation of NUP153 (S334) and NUP214 (S940/S1055), suggesting a possible role for SCAMP3 in coordinating ERK1/2 access to nuclear transcriptional regulators. This was accompanied by decreased phosphorylation of E74-like ETS transcription factor (ELF1, S187) and ELF4 (S188), transcription factors implicated in cancer survival and therapy resistance. ELF1 has been shown to suppress autophagy and modulate chemoresistance via the miR-152-3p/neural cell adhesion molecule 1 (NCAM1)/ERK1/2 axis [58], while ELF4 phosphorylation influences its activation and degradation in response to DNA damage and cellular stress [59,60].

Beyond transcriptional control, SCAMP3 loss altered the phosphorylation of metabolic enzymes. Tumor cells reprogram metabolic pathways to adapt to nutrient fluctuations [37,61]. Our data evidence that SCAMP3 is associated with the phosphorylation of enzymes involved in glycolysis (triosephosphate isomerase, TPI1), lipogenesis (ATP-citrate lyase, ACLY), and acetate metabolism (acetyl-CoA synthetase 2, ACSS2). The phosphorylation of TPI1 at S21 has been linked to the prevention of toxic metabolite accumulation, such as methylglyoxal, which contributes to oxidative stress and DNA damage [33,36]. This site is reportedly phosphorylated by protein kinase A (PKA) or cyclin-dependent kinase 2 (CDK2) under metabolic or genotoxic stress, correlating with poor prognosis and metastatic potential [34,35].

SCAMP3 knockout also reduced phosphorylation of ACLY at S455, a site regulated by AKT/mTORC2 [39]. ACLY generates acetyl-CoA for lipid synthesis and histone acetylation, processes that support proliferation and epigenetic plasticity [37,38]. Hatipoglu et al. reported that phosphorylation of ACLY at S455 is essential for the survival of KRas-driven breast cancer cells under glutamine-deprived conditions [38]. This aligns with our previous findings showing SCAMP3 overexpression increases p-AKT at S473 [11]. In our study, MK-8353 increased the phosphorylation of ACSS2A, which catalyzes the conversion of acetate to acetyl-CoA [62,63]. Phosphorylation of ACSS2A at S267, reduced in SC3KO cells, has been associated with tumor growth and disease progression [64,65], and is regulated by cyclin-dependent kinase 5 (CDK5) to promote protein stability and resistance to tamoxifen [66,67].

Our data also suggest that SCAMP3 may cooperate with ERK1/2 to regulate choline-phosphate cytidylyltransferase A (PCYT1A/CCT-α), the enzyme responsible for phosphatidylcholine (PC) synthesis. Elevated PC synthesis supports rapid proliferation and membrane biogenesis in cancer cells, and altered PC metabolism contributes to tumorigenesis and therapy resistance by promoting DNA repair, autophagy, and lipid droplet formation [68,69]. ERK1/2-mediated phosphorylation PCYT1A/CCT-α at S315 suppresses its activity and retains it in the cytosol, while phosphorylation at S362 and Y359 promotes its translocation to the nuclear envelope, enabling PC production [42,43]. Changes in ACSS2A and PCYT1A/CCT-α phosphorylation suggest that SCAMP3 may influence acetate utilization and PC biosynthesis, pathways essential for membrane biogenesis.

Our findings reveal a broader role for SCAMP3 in sustaining receptor signaling networks. SCAMP3 loss reduced phosphorylation of cluster of differentiation 44 (CD44) at S697, a site linked to cell motility [46]. CD44 is a well-established CSC marker that promotes epithelial–mesenchymal transition (EMT) and metastasis, and is frequently upregulated in TNBC, where it correlates with poor prognosis [70,71]. CD44 interacts with epidermal growth factor receptor (EGFR) to support tumor initiation [71], and its inhibition accelerates EGFR degradation, affecting downstream signaling and enhancing drug sensitivity [72]. SC3KO also reduced the expression of CD44 in response to EGFR stimulation. Aligned with SCAMP3 loss promotes EGFR degradation, SCAMP3 may help sustain CD44 expression when EGFR is active [11].

SCAMP3 also influences Roundabout homolog 1 (ROBO1) phosphorylation dynamics. While ROBO1 functions as a Myc activity suppressor, its role in TNBC is complex [47]. Recent studies report high levels of ROBO1 in brain metastases of breast cancer patients [48,73]. Furthermore, the TNBC cell line MDA-MB-231 expresses high levels of ROBO1 ligand, SLIT2, which promotes migration and invasion [48,74]. In our study, SCAMP3 knockout reduces ROBO1 S1055 phosphorylation while increasing S940 phosphorylation, the latter being linked to increased invasiveness [49]. These findings raised the possibility that SCAMP3 modulates ROBO1 signaling dynamics in TNBC, potentially influencing invasive behavior. However, further mechanistic studies are needed to clarify the functional significance of these phosphorylation changes.

Beyond ERK1/2, SCAMP3 affects substrates of multiple kinases. Pseudopodium enriched atypical kinase (PEAK1)-related kinase-activating pseudokinase 1 (PEAK2), a pseudokinase and scaffold protein involved in Abl- and CSK-mediated cancer motility [28,29], showed reduced phosphorylation at S745 in SC3KO cells. This site is regulated by the tumor suppressor PRP4K, which is involved in mitosis and EGFR degradation [30,75,76,77]. Kinase enrichment analysis predicted casein kinase 2 (CK2) as a major kinase, with substrates affected by SCAMP3 loss. CK2 is a serine/threonine kinase composed of two catalytic (α or α’) and two regulatory (CK2β) subunits and is involved in diverse oncogenic processes [78]. SCAMP3 knockout increased phosphorylation of CK2β at S209, a site phosphorylated by cyclin-dependent kinase 1 (CDK1) that enhances CK2 activity [79]. Additionally, SCAMP3 loss reduced CK2-dependent phosphorylation of calnexin at S554. This modification regulates calnexin’s interaction with the cytosolic protein phosphofurin acidic cluster sorting protein 2 (PACS-2) and its trafficking to the cell surface, a process linked to cancer control [32,80]. While these findings are consistent with known roles of these proteins in cancer progression, further experiments are necessary to validate their interaction with SCAMP3.

A critical finding of our study is the role of SCAMP3 as a coordinator of ERK1/2 signaling with autophagy regulation. This process involves autophagosome formation, maturation, and fusion with lysosomes, regulated by nutrient-sensing pathways such as mTOR [81]. Under nutrient-rich conditions, mTORC1 inhibits autophagy by interacting with the Ras-related protein Rab-7a (RAB7A) GTPase-activating protein TBC1 domain family member 5 (TBC1D5), thereby preventing RAB7A activation. RAB7A is central for late endosome lysosome fusion and autophagosome maturation, recruiting tethering and fusion machinery for lysosomal degradation [50]. During nutrient starvation or mTORC1 inhibition, TBC1D5 associates with microtubule-associated protein 1 light chain 3 (LC3) to redirect endosomal cargo toward degradation [50,51,52,82,83].

SCAMP3 knockout impairs autophagic flux, evidenced by reduced levels of Rab7, optineurin, and sequestosome (SQSTM1/p62), along with LC3B-II accumulation. Moderate ERK1/2 activation supports protective autophagy by promoting mTORC1 translocation to late endosomes or lysosomes [84,85]. In our study, ERK inhibition amplified autophagy defects in SC3KO cells. These findings suggest that SCAMP3 may contribute to ERK activation and help maintain the coupling between ERK signaling and autophagy, supporting efficient degradation of damaged or surplus cellular components.

In summary, our comprehensive analysis positions SCAMP3 as a master regulator of oncogenic signaling in TNBC. We reveal SCAMP3 as a multifunctional scaffold that sustains ERK1/2 activity. The changes in the phosphorylation landscape suggest that SCAMP3 depletion prevents compensatory responses that typically limit therapeutic efficacy. This finding identifies SCAMP3 as a potential biomarker for predicting ERK target therapy response and a therapeutic target for overcoming adaptive resistance. The multi-pathway regulation by SCAMP3 suggests that targeting this protein could provide broader therapeutic benefits than single-pathway inhibition.

## 4. Materials and Methods

### 4.1. Cell Culture and Reagents

The human triple negative breast cancer (SUM-149) cells were purchased from BiolVT Inc. (Westbury, NY, USA) and cultured in Ham’s F-12 nutrient mix (Gibco/Life Technologies, Waltham, MA, USA) supplemented with 10% FBS. The knockout of SCAMP3 in SUM-149 cells was achieved using CRISPR/Cas9 as described in [4]. Cell lines used in this study were authenticated by short tandem repeat (STR) profiling and screened for Mycoplasma with the Mycoplasma Detection Kit (Nordic BioSite AB, Täby, Sweden) before use.

### 4.2. Immunoblotting

Untreated SUM-149 wild-type (WT) and *SCAMP3* knockout (SC3KO) cells remained in complete medium, while the others were serum-starved for 18 h. Quiescent cells were then stimulated with 10 ng/mL EGF for 30 min, followed by 8 μM MK-8353 for 2 h. Cells were then lysed and processed for immunoblotting following the procedures outlined in [4] for the specified primary antibodies. Antibodies against p44/42 MAPK (ERK1/2) (9102), optineurin (E4P8C) (70928), SQSTM1/p62 (8025), LC3B (2775), mTOR (7C10) (2983), phospho-mTOR (S2448) (5536), CD44 (156-3C11) (3570), and β-actin (8H10D10) (3700) were purchased from Cell Signaling Technology (Danvers, MA, USA). Ran antibody (sc-271376) was purchased from Santa Cruz Biotechnology (Dallas, TX, USA). Anti-RAB7A recombinant antibody (84741-1-RR), phospho-p70S6K (T389) (82373-1-RR), phospho-rpS6 (S235/236) (80130-2-RR), and phospho-4E-BP1 (T37) (81812-4-RR) were purchased from Proteintech Group, Inc. (Rosemont, IL, USA). Anti-SCAMP3 antibody (PA5-21428) was purchased from ThermoFisher Scientific (Waltham, MA, USA). Anti-RAB7A (84741-1-RR) was purchased from Proteintech Inc. (Rosemont, IL, USA). Western blot analysis for p-ERK1/2 was performed using the phospho-p44/42 MAPK (p-ERK1/2 Thr202/Tyr204) antibody (Cell Signaling Technology, Danvers, MA, USA; Cat #4370). This antibody detects endogenous levels of ERK1 and ERK2 when dually phosphorylated at the canonical Thr202/Tyr204 (for ERK1) and Thr185/Tyr187 (for ERK2) activation sites.

### 4.3. Protein Extraction

SUM-149 wild-type and SCAMP3 knockout cells were cultured and treated as described above. After treatment, cells were lysed in a buffer containing 10 mM Tris (pH 8.0), 100 mM NaCl, 1 mM EDTA, 1% Triton X-100, 10% glycerol, 1 mM EGTA, 1 mM NaF, 20 mM tetrasodium pyrophosphate, 2 mM sodium orthovanadate, 0.5% sodium deoxycholate, and 0.1% SDS. The buffer was freshly supplemented with phosphatase inhibitor cocktails I and II (MedChemExpress, Monmouth Junction, NJ, USA) and the Complete Mini Protease Inhibitor Cocktail (Sigma Aldrich, St. Louis, MO, USA). To ensure complete disruption, lysates were briefly sonicated and then centrifuged at 14,000 rpm for 10 min. Total protein concentration was measured using the Precision Red Advanced Protein Assay Reagent (Cytoskeleton Inc., Denver, CO, USA) with a BioMate 160 UV-Visible Spectrophotometer (Thermo Fisher Scientific, Waltham, MA, USA).

### 4.4. Sample Processing

Isolated protein samples (n = 24) were aliquoted (1.03 mg) and normalized. Proteins were reduced by incubation with DL-dithiothreitol (DTT) to a final concentration of 2.5 mM DTT for 40 min at 55 °C and alkylated with iodoacetamide (IAA) to a final concentration of 7.5 mM IAA for 40 min at room temperature, in the dark. Alkylation was quenched by adding DTT to a final concentration of 5 mM. In-solution digestion with trypsin (Pierce) was performed overnight at 37 °C in a trypsin/protein ratio of 1:100. Samples were acidified with formic acid (FA) to a final concentration of 1% and centrifuged, and the supernatant was recovered. The samples were cleaned using OASIS MCX 3cc columns (Waters Corp., Milford, MA, USA). Columns were first conditioned with 100% acetonitrile, 50% acetonitrile, and 0.1% TFA. Samples were loaded onto columns, washed with 0.1% TFA, and eluted with 0.1% TFA/50% acetonitrile. Elutions were dried using speed vacuum. Internal controls were prepared by mixing the remaining amounts from all baseline WT samples (n = 4) to gather 3 mg of total protein. The mixture was vortexed and divided into three aliquots, one for each TMT kit (Thermo Fisher Scientific, Waltham, MA, USA). Each aliquot was submitted to the same sample processing.

#### 4.4.1. Phosphopeptide Enrichment

Phosphopeptide enrichment was performed following the manufacturer’s protocol for the High-SelectTM Fe-NTA phosphopeptide enrichment kit (A32992, Thermo Fisher Scientific, Waltham, MA, USA). Briefly, 1.03 mg of total protein digest was suspended in the binding/wash buffer. Columns were equilibrated with the same buffer and centrifuged twice at 1000× *g* for 30 s. Phosphopeptides were then bonded to the column by loading the sample, gently mixing with the resin, and incubating for 30 min with occasional gentle mixing. Columns were then centrifuged at 1000× *g* for 30 s. Samples were washed with buffer and water and eluted with the elution buffer provided. Elutions were dried using speed vacuum (Vacufuge Plus, Eppendorf, MA, USA). After performing phosphopeptide enrichment, proteomics analysis identified 16,654 total peptides, 4408 phosphopeptides, and 12,246 non-phosphopeptides. The percentage of phosphopeptide enrichment (Phosphopeptides/non-phophopeptides × 100) was 35.99% (Table S5).

#### 4.4.2. Tandem Mass Tag (TMT) Labeling

TMT labeling was performed following the manufacturer’s instructions for the TMT11-131C Label Reagent Kit (A34808, Thermo Fisher Scientific, Waltham, MA, USA). TMT reagents were reconstituted in acetonitrile (41 μL for 0.8 mg), and dry digested in 100 mM triethyl ammonium bicarbonate (TEAB). After labeling, samples were incubated for 1 h with gentle shaking and a quenching step of 15 min. Finally, equal amounts of each sample were mixed to generate a final pool for each TMT set that was dried and later subjected to fractionation. For this study, three TMT kits were used, and sample pools were generated with the 24 samples as described in Table S1. Six (n = 6) sample groups were included with four independent biological replicates per group for a total of 24 samples. For the internal standard, an aliquot from a pooled mixture of four representative wild-type untreated samples was included in each of the three kits.

#### 4.4.3. Fractionation

The fractionation of peptides was performed using the Pierce^TM^ High pH Reversed-Phase Peptide Fractionation Kit (89875, Thermo Fisher Scientific, Waltham, MA, USA) and following the manufacturer’s instructions. The column was subjected to two conditionings using 300 μL of acetonitrile, followed by a 2 m centrifugation at 5000× *g*. Subsequently, the procedures were repeated using 0.1% TFA. The TMT-labeled pool was reconstituted in 300 μL of 0.1% TFA, loaded onto the column, washed, and eluted eight times using a series of elution solutions with different percentages of acetonitrile/0.1% triethylamine and centrifugation at 3000× *g* for 2 min. Three TMT pools were submitted to this procedure, and 24 fractions were recovered, dried, and stored at −80 °C until LC-MS/MS analysis.

#### 4.4.4. LC-MS/MS Analysis

The liquid chromatography and mass spectrometry (LC-MS/MS) analysis was performed using an Easy nLC 1200 (Thermo Scientific, Waltham, MA, USA) coupled to the Q-Exactive Plus (Thermo Scientific, Waltham, MA, USA). A PicoChip chromatographic column (New Objective) was used with the following specifications: H354 REPROSIL-Pur C18-AQ (3–5 μm), 120–300 Å, and a 105 mm bed length. Mobile phases for the gradient consisted of 0.1% formic acid in water (Buffer A) and 0.1% formic acid in 80% acetonitrile (Buffer B). Peptide separation was obtained using a gradient of 7–25% of Buffer B for 102 min, 25–60% of Buffer B for 20 min, and 60–95% Buffer B for 6 min. The total gradient time was 128 min at a flow rate of 300 nl/min, a maximum pressure of 300 bars, and an injection volume of 2 μL per sample. The Q-Exactive Plus MS instrument operates in the positive polarity mode and data-dependent mode. The full scan was measured over the range of 375 to 1400 at a resolution of 70,000. The default charge state is 2. The AGC target for the full MS is set to 3 × 10 ^6^. The MS2 analysis was configured to select the ten (10) most intense ions (Top10) for HCD fragmentation with a resolution of 35,000, an AGC target of 1 × 10^5^, and a 1.2 *m*/*z* isolation window. Collision energy was set to 32. A dynamic exclusion parameter was set for 30 s.

#### 4.4.5. Database Analysis

Raw mass spectrometric data files were analyzed using the software Proteome Discoverer (PD), v.2.5 (Thermo Fisher Scientific, Waltham, MA, USA). Files were searched against a human database downloaded using the PD Protein Center tool (tax ID = 9606) containing 42,380 sequences. Sequest HT was used for the search. Trypsin was specified as the enzyme for proteolysis with a maximum of 2 mixed cleavage sites. Precursor mass tolerance was set to 20 ppm, and fragment mass tolerance was set to 0.5 Da. The modifications included a dynamic modification for Phospho of +79.966 Da (S, T, Y), a static modification of +57.021 Da (C), and static modifications of the TMT reagents +229.163 Da (Any N Term, K). Values in the TMT certificate of analysis (Lot: WJ322334) were included to correct for reporter ion isotopic impurities. Mass analyzer was set to FTMS. The percolator node was included for target/decoy selection, and FDR targets were set to 0.01 (Strict) and 0.05 (Relaxed). All sample conditions were normalized against the internal control. These conditions resulted in a medium to high level of confidence, by only considering proteins with two or more #unique peptides, removing keratins, and showing only master proteins. Protein hits were 8522 and 2084 proteins after filters were applied (Tables S6 and S7).

### 4.5. Identification of Dysregulated Proteins and Statistical Analyses

Before the statistical analysis, 8522 proteins were processed and normalized according to the design described in Table S1, using Proteome Discoverer v.2.5 (Thermo Fisher Scientific, Waltham, MA, USA). Scaled abundances from Proteome Discoverer were used for the analysis. Using the public license of MetaboAnalyst6.0 [86], data were filtered using the relative standard deviation (RSD), calculated as the standard deviation divided by the mean. Features with a ≥20% RSD were omitted from the following analysis. Missing values were estimated using the machine learning feature-wise technique K-Nearest Neighbor (KNN). A one-factor analysis was used to compare cases and controls. Statistical analysis based on a fold change (Log_2_FC) of 1.5 and a *t*-test *p*-value ≤ 0.05 identified proteins that differed in abundance between group comparisons. Given the limited sample size (4 replicates per group), FDR-adjusted p-values were not considered reliable due to low statistical power, as noted in previous proteomics studies [87,88,89,90]. Therefore, raw *p*-values and fold changes were reported to identify candidate phospho-peptides, and the results were interpreted as exploratory. The proteins of interest were subsequently validated using Western blot analysis. Dysregulated proteins were visualized in volcano plots generated in MetaboAnalyst 6.0. Furthermore, a list of phosphoproteins was obtained for each comparison group (n = 4) to generate a Venn diagram using Excel’s custom sorting and repeated measures tools.

### 4.6. Ingenuity Pathway Analysis (IPA)

The statistical analysis results were used for enrichment analysis with the Ingenuity Pathway Analysis Software (IPA, version 24.0.1, QIAGEN Digital Insights, Germantown, MD, USA). The lists of differentially expressed phosphoproteins were annotated using the accession numbers obtained during the IPA enrichment analysis. An Ingenuity CORE analysis was implemented to enrich the dataset, identify canonical pathways. A log_2_FC ≥ 1.5 and a *p*-value of ≤0.05 were considered significant in the differentially expressed phosphoproteins; a –log10 of *p* ≥ 1.5 (or *p* ≤ 0.05) was considered significant for the Canonical Pathway enrichment analyses.

### 4.7. Kinase Enrichment Analysis

Kinase substrate enrichment analysis was performed using K3 kinase enrichment analysis 3 (IKEA3), version 3.0 (https://maayanlab.cloud/kea3/ (accessed on 30 June 2025)) [91]. Hyperphosphorylated or hypophosphorylated proteins in the SC3KO vs. WT dataset were used as input to identify enriched kinases within the dataset.

### 4.8. STRING Consortium Analysis Software

The protein–protein interaction network was generated using the STRING database (version 12.0) provided by the String Consortium 2024. The network was constructed with an interaction confidence score of 0.400. The analysis included known and predicted interactions based on experimental data, neighborhood, gene fusion, curated databases, co-expression, textmining, and co-occurrence. Edge thickness reflects the confidence level of interactions.

### 4.9. Statistical Analysis

Statistical analysis was performed using GraphPad Prism 10.4.2 (Dotmatics, San Diego, CA, USA) with analysis of variance (ANOVA). A *p* ≤ 0.05 was considered statistically significant.

## 5. Conclusions

In conclusion, this study highlights SCAMP3 as a potential regulator of multiple oncogenic pathways. Further experimental validation is necessary to confirm its role in receptor signaling, metabolic reprogramming, and kinase regulation. These insights lay the groundwork for future investigations into SCAMP3 as a candidate target for cancer therapeutic interventions.

## Figures and Tables

**Table 1:** ERK1/2 targets identified as deregulated in the NT: SC3KO vs. WT comparison. The table lists ERK targets deregulated in the phosphoproteomics analysis based on datasets from https://sys-bio.net/erk_targets/targets_all.html (accessed on 30 June 2025) and PhosphoSitePlus^®^.

Accession No.	Gene	Description	Phosphosite	log2(FC) ^1^
P35658	*NUP214*	Nuclear pore complex protein Nup214	S940 ↓	−3.21
P27824	*CANX*	Calnexin	S554 ↓	−1.98
P36507	*MAP2K2*	Dual specificity mitogen-activated protein kinase kinase 2	T394 ↓	−1.94
P04049	*RAF1*	RAF proto-oncogene serine/threonine-protein kinase	S43 ↓	−1.59
Q96CV9	*OPTN*	Optineurin	S526 ↓	−1.50
Q13501	*SQSTM1*	Sequestosome-1	S272 ↓	−1.47
Q92609	*TBC1D5*	TBC1 domain family member 5	S554 ↓	−1.46
P28482	*MAPK1*	Mitogen-activated protein kinase 1	T185 ↓	−1.15
P49790	*NUP153*	Nuclear pore complex protein Nup153	S209 ↓	−2.00
			S334 ↑	+1.99

^1^ Negative values indicate downregulated phosphosites and are marked with blue arrows, while positive values indicate upregulated phosphosites and are marked with orange arrows. Phosphsites in the table are ordered from the most downregulated to the least downregulated, followed by the most upregulated to the least upregulated, considering a threshold of Log_2_FC ≥ 1.5 and a *p* ≤ 0.05.

**Table 2:** Phosphosites identified as deregulated in the NT: SC3KO vs. WT dataset. The table lists deregulated phosphoproteins and phosphosites identified as deregulated in the NT: SC3KO vs. WT dataset and their corresponding putative kinases based on data from PhosphoSitePlus^®^.

Accession No.	Gene	Protein Description	Phosphosite	log_2_(FC) ^1^	Putative Kinase
P08238	*HSP90AB1*	Heat shock protein 90β	S226 ↓	−3.41	CK2alpha1
			S255 ↓	−3.05	
Q9NTI5	*PDS5B*	Sister chromatid cohesion protein PDS5 homolog B	T1370 ↓	−3.24	CDK1
Q14134	*TRIM29*	Tripartite motif-containing protein 29	S552 ↓	−3.11	MAPK2
P60174	*TPI1*	Triosephosphate isomerase	S21 ↓	−2.92	SIK, PKA, CDK2
Q8WWI1	*LMO7*	LIM domain only protein 7	S751 ↓	−2.75	AurB
Q14980	*NUMA1*	Nuclear mitotic apparatus protein 1	S1969 ↓	−2.60	AurA-B
P29966	*MARCKS*	Myristoylated alanine-rich C-kinase substrate	S170 ↓	−2.49	PRKCA
Q16181	*SEPTIN7*	Septin-7	T426 ↓	−2.45	TAO1/2
Q14814	*MEF2D*	Myocyte-specific enhancer factor 2D	S231 ↓	−2.43	MAPK13/14
Q92974	*ARHGEF2*	Rho guanine nucleotide exchange factor 2	S960 ↓	−2.31	CDK1
Q8WX93	*PALLD*	Palladin	S641 ↓	−2.30	CDK1
Q96E09	*PABIR1*	PPP2R1A-PPP2R2A interacting phosphatase regulator 1	S37 ↓	−2.16	CHK1
Q86YV5	*PRAG1*	Inactive tyrosine-protein kinase PRAG1	S745 ↓	−2.06	PRP4K
P17096-2	*HMGA1*	High mobility group protein HMG-I/HMG-Y	T42 ↓	−2.04	CDK1 HIPK2
Q14247	*CTTN*	Src substrate cortactin	T401 ↓	−1.93	AMPKA1
P16070	*CD44*	CD44 antigen	S697 ↓	−1.87	PRKCA
Q86VM9	*ZC3H18*	Zinc finger CCCH domain-containing protein 18	S534 ↓	−1.82	PLK1
P53396	*ACLY*	ATP-citrate synthase	S455 ↓	−1.75	AKT1
P06702	*S100A9*	Protein S100-A9	T113 ↓	−1.73	MAPK14
Q9UQN3	*CHMP2B*	Charged multivesicular body protein 2b	S199 ↓	−1.68	TBK1
Q9NZT2	*OGFR*	Opioid growth factor receptor	S378 ↓	−1.65	CDK1
P04049	*RAF1*	RAF proto-oncogene serine/threonine-protein kinase	S43 ↓	−1.59	PRKCA
Q9UQE7	*SMC3*	Structural maintenance of chromosomes protein 3	S1067 ↓	−1.57	ATM; CK2alpha1, CK2alpha2
Q9UKV3	*ACIN1*	Apoptotic chromatin condensation inducer in the nucleus	S183 ↓	−1.56	Pim1
Q13501	*SQSTM1*	Sequestosome-1	S272 ↓	−1.47	CDK1; CDKL5
Q96D71	*REPS1*	RalBP1-associated Eps domain-containing protein	S709 ↓	−1.46	p90RSK
P67870	*CSNK2B*	Casein kinase II subunit beta	S209 ↑	+3.06	CDK1
P49585	*PCYT1A*	Choline-phosphate cytidylyltransferase A	S362 ↑	+2.48	CK2alpha1
Q92597	*NDRG1*	Protein NDRG1	S330 ↑	+2.19	Pim1; PRKCA
Q9NSK0	*KLC4*	Kinesin light chain 4	S590 ↑	+2.17	AMPKA2
Q8ND76	*CCNY*	Cyclin-Y	S326 ↑	+1.85	AMPKA1

^1^ Negative values indicate downregulated phosphosites and are marked with blue arrows, while upregulated phosphosites indicate positive values marked with orange arrows. Phosphoproteins in the table are ordered from the most downregulated to the least downregulated, followed by the most upregulated to the least upregulated. Refer to the list of abbreviations for the description of kinase names.

## Data Availability

The original data presented in the study are openly available in ProteomeXchange at 10.6019/PXD064784 or PXD064784 accession number.

## Supplementary Materials

The following supporting information can be downloaded at: https://www.mdpi.com/article/10.3390/ijms26199577/s1.

## Abbreviations

The following abbreviations are used in this manuscript:

4EBP1	Eukaryotic translation initiation factor 4E-binding protein 1
ABL1	Abelson tyrosine-protein kinase 1
ACIN1	Apoptotic chromatin condensation inducer in the nucleus
ACLY	ATP-citrate lyase
ACSS2A	Acetyl-CoA synthetase 2
AKT1	Protein kinase B
AMPKA1	5’-AMP-activated protein kinase catalytic subunit α1
ATG9	Autophagy-related 9A
ATM	Ataxia telangiectasia mutated
AURKA, B	Aurora kinase A, B
CANX	Calnexin
CCT-α	choline-phosphate cytidylyltransferase A
CD44	Cluster of differentiation 44
CDK	Cyclin-dependent kinase
CHK1	Serine/threonine-protein kinase Chk1
CK2	Casein kinase 2
CoA	Coenzyme A
CSCs	Cancer stem cells
CSK	C-terminal Src kinase
CK2alpha1	Casein kinase 2 alpha 1
CK2alpha2	Casein kinase 2 alpha 2
DHAP	Dihydroxyacetone phosphate
DTT	DL-dithiothreitol
EGFR	Epidermal growth factor receptor
ELF	E74 like ETS transcription factor
EMT	Epithelial–mesenchymal transition
ER	Estrogen receptor
ERK 1/2	Extracellular signal-regulated kinase 1/2
ETS1	Protein C-ets-1
FBS	Fetal bovine serum
FG	Phenylalanine glycine
GOLGA4	Golgin subfamily A member 4
GRB2	Growth factor receptor bound protein 2
GSK3B	Glycogen synthase kinase 3β
HER2	Human epidermal growth factor receptor 2
HIPK2	Homeodomain-interacting protein kinase 2
HSP90AB1	Heat shock protein HSP 90-beta
IAA	Iodoacetamide
IKEA3	Kinase enrichment analysis 3
IPA	Ingenuity Pathway Analysis Software
JAK	Janus kinase
KLC4	Kinesin light chain 4
KNN	K-Nearest Neighbor
KSR1	Ras kinase suppressor
LMNB1	Lamin-B1
LC3	Microtubule-associated protein light chain 3
LKB1	Liver kinase B1
MAP1LC3B	Microtubule-associated protein 1 light chain 3 beta
MAP2K2	Dual specificity mitogen-activated protein kinase kinase 2
MAPK	Mitogen-activated protein kinase
MAPK13,14	Mitogen-activated protein kinase 13, 14
MAPKAPK2	MAP kinase-activated protein kinase 2
MDR1	Multidrug resistance protein
MEK1/2	Dual-specificity mitogen-activated protein kinase kinase 1, 2
MGO	Methylglyoxal
MKK4/JNK	c-Jun N-terminal kinase/c-Jun NH 2 terminal kinase
mTOR	Mammalian target of rapamycin
NCAM1	Neural cell adhesion molecule 1
NDRG1	N-myc downstream-regulated gene 1
NUP153	Nuclear pore complex protein 153
OPTN	Optineurin
p70S6K	Ribosomal protein S6 kinase
p90RSK	Ribosomal Protein S6 Kinase A1
PACS2	Phosphofurin acidic cluster sorting protein 2
PAK 4	Serine/threonine-protein kinase PAK 4
PCYT1A	Choline-phosphate cytidylyltransferase A
PEAK2	Pseudopodium enriched atypical kinase (PEAK1)-related kinase-activating pseudokinase 1
PI3K	Phosphatidylinositol 3-kinase
PI4K2A	Phosphatidylinositol 4-kinase type 2-alpha
PIM1	Serine/threonine-protein kinase Pim-1
PKA	Protein kinase A
PLEC	Plectin
PLK1	Polo-like kinase 1
PPP1R10	serine/threonine-protein phosphatase 1 regulatory subunit 10
PPP2CA	Protein phosphatase 2a
PR	Progesterone receptor
PRKACA	Protein Kinase cAMP-Activated Catalytic Subunit α
PRKCA	Protein kinase Cα
PRKCD	Protein kinase C delta type
PRP4K	pre-mRNA processing factor kinase
Rab7A	Ras-related protein Rab-7a
Raf-1	RAF proto-oncogene serine/threonine-protein kinase
RAS	Rat sarcoma
ROBO1	Roundabout homolog 1
rpS6	Ribosomal subunit protein S6
SC3KO	SCAMP3 knockout
SCAMP3	Secretory carrier membrane protein 3
SFKs	Src family kinases
SIK	Salt-inducible kinase
SLK	STE20-like serine/threonine-protein kinase
SOS	SOS Ras/Rac Guanine Nucleotide Exchange Factor 1
SQSTM1	Sequestosome-1
Src	Proto-oncogene tyrosine-protein kinase Src
STAT	Signal transducer and activator of transcription
STR	Short tandem repeat
TAO1,2	TAO Kinase 1, 2
TBC1D5	TBC1 domain family member 5
TBK1	TANK-binding kinase 1
TFA	Trifluoroacetic acid
TJP1	Tight junction protein ZO-1
TMT	Tandem mass tag
TNBC	Triple negative breast cancer
TPI1	Triosephosphate isomerase
TPR	Translocated promoter region protein
TSC1	Tuberous sclerosis 1
WT	Wild type
