# Supplementary material for: SCAMP3-Driven Regulation of ERK1/2 and Autophagy Phosphoproteomics Signatures in Triple-Negative Breast Cancer
Source: Int J Mol Sci. 2025 Oct 1;26(19):9577. doi: 10.3390/ijms26199577 (PMC12525412; doi:10.3390/ijms26199577)
Supplement: Supplementary file 1 [file ijms-26-09577-s001.zip › ijms-3795029-supplementary.pdf]

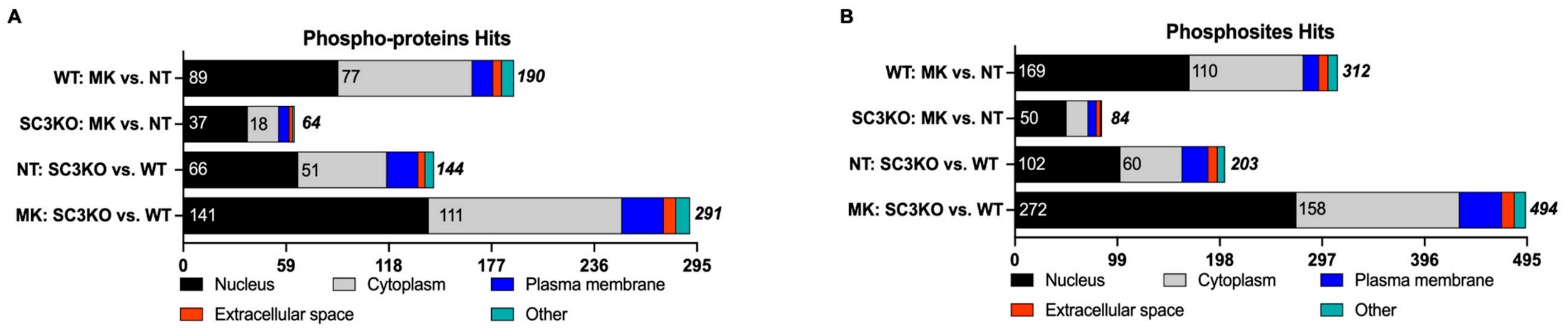

Figure S1: Subcellular distribution of differentially expressed phosphoproteins and phosphosites. Subcellular distribution of differentially expressed **(A)** phosphoproteins and **(B)** phosphosites was determined across the compared groups. Statistical analysis was performed using MetaboAnalyst 5.0 with a significance threshold of $p \leq 0.05$ and a Log2 fold change (FC) ≥ 1.5. Functional analysis was performed using Ingenuity Pathway Analysis (IPA) version 24.0.1.

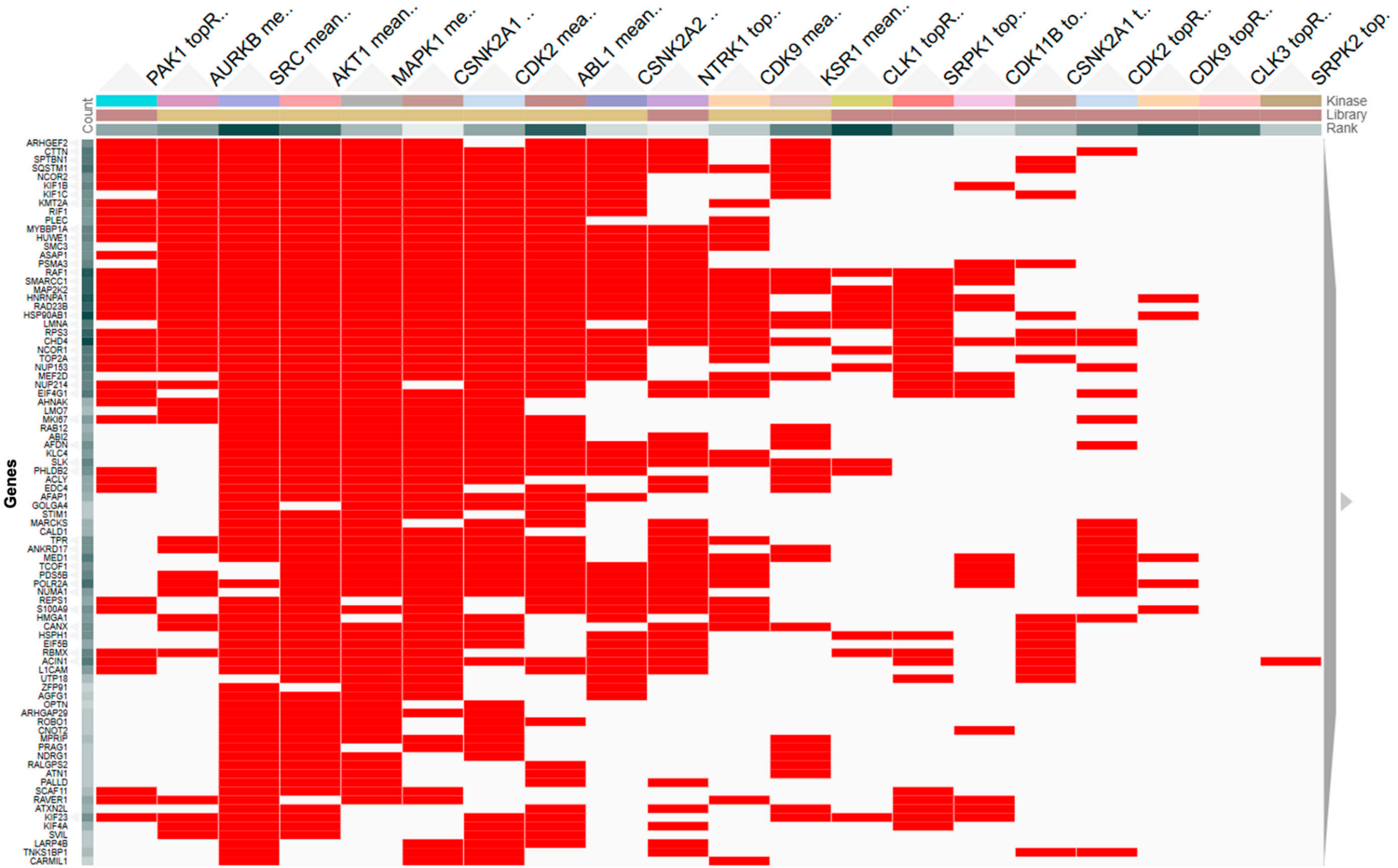

Figure S2. Kinase enrichment analysis of hypophosphorylated proteins in the SC3KO vs. WT group. Integrated kinase enrichment analysis using the MeanRank method. Clustergrammer visualization displaying enriched kinases and their substrates identified as hypophosphorylated in the SC3KO vs. WT group. Columns correspond to kinases; rows correspond to hypophosphorylated substrate proteins from the input list. Kinase enrichment was performed using software K3 Kinase Enrichment Analysis 3 (KEA3) version 3.0.

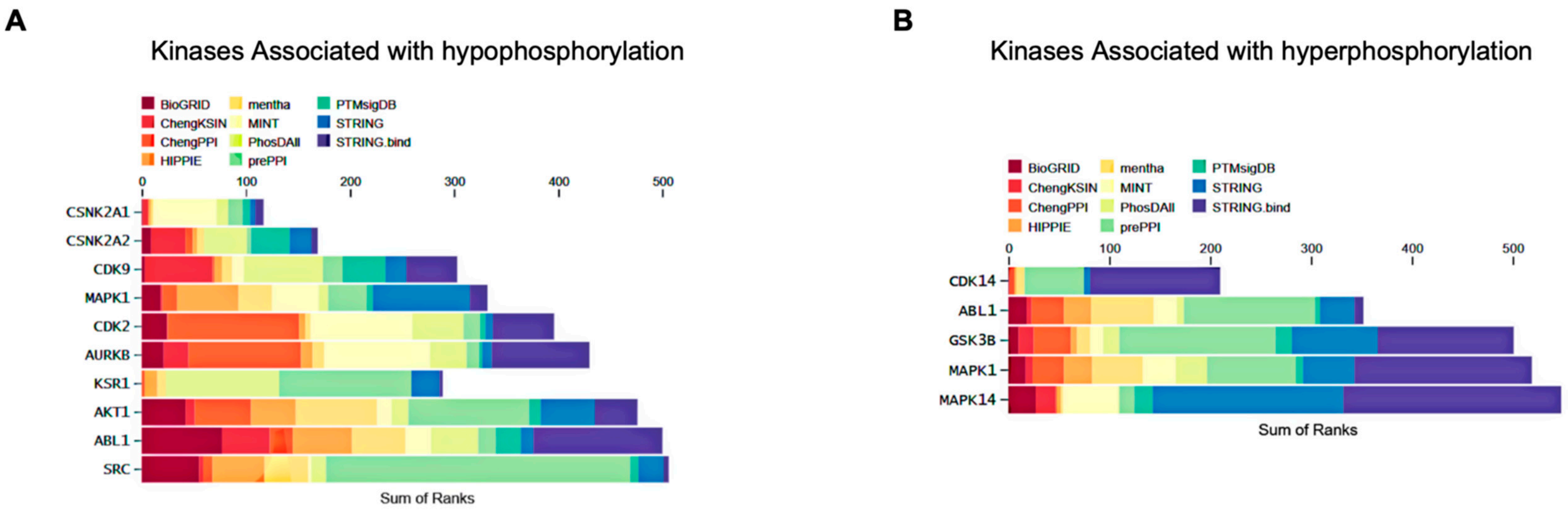

Figure S3. Kinase enrichment analysis of hypophosphorylated and hyperphosphorylated proteins in the SC3KO vs. WT group. Integrated kinase ranking across libraries for **(A)** hypophosphorylated and **(B)** hyperphosphorylated proteins. Each colored segment represents individual library rankings for a specific kinase. Kinase enrichment was performed with software K3 Kinase Enrichment Analysis 3 (KEA3) version 3.0 using the MeanRank method.

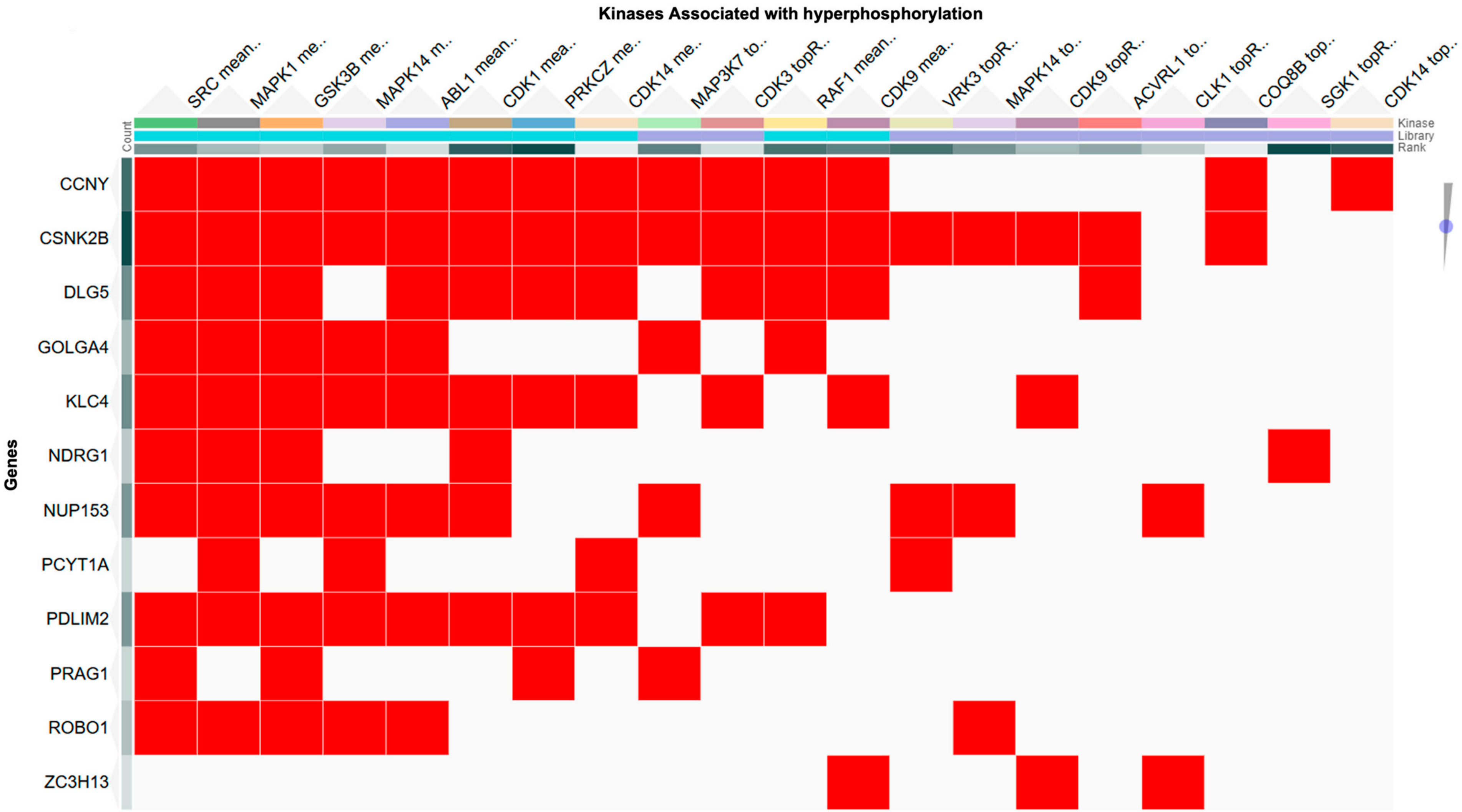

Figure S4. Kinase enrichment analysis of hyperphosphorylated proteins in the SC3KO vs. WT group. Integrated kinase enrichment analysis using the MeanRank method. Clustergrammer visualization displaying enriched kinases and their substrates identified as hyperphosphorylated in the SC3KO vs. WT group. Columns correspond to kinases; rows correspond to hyperphosphorylated substrate proteins from the input list. Kinase enrichment was performed using software K3 Kinase Enrichment Analysis 3 (KEA3) version 3.0.

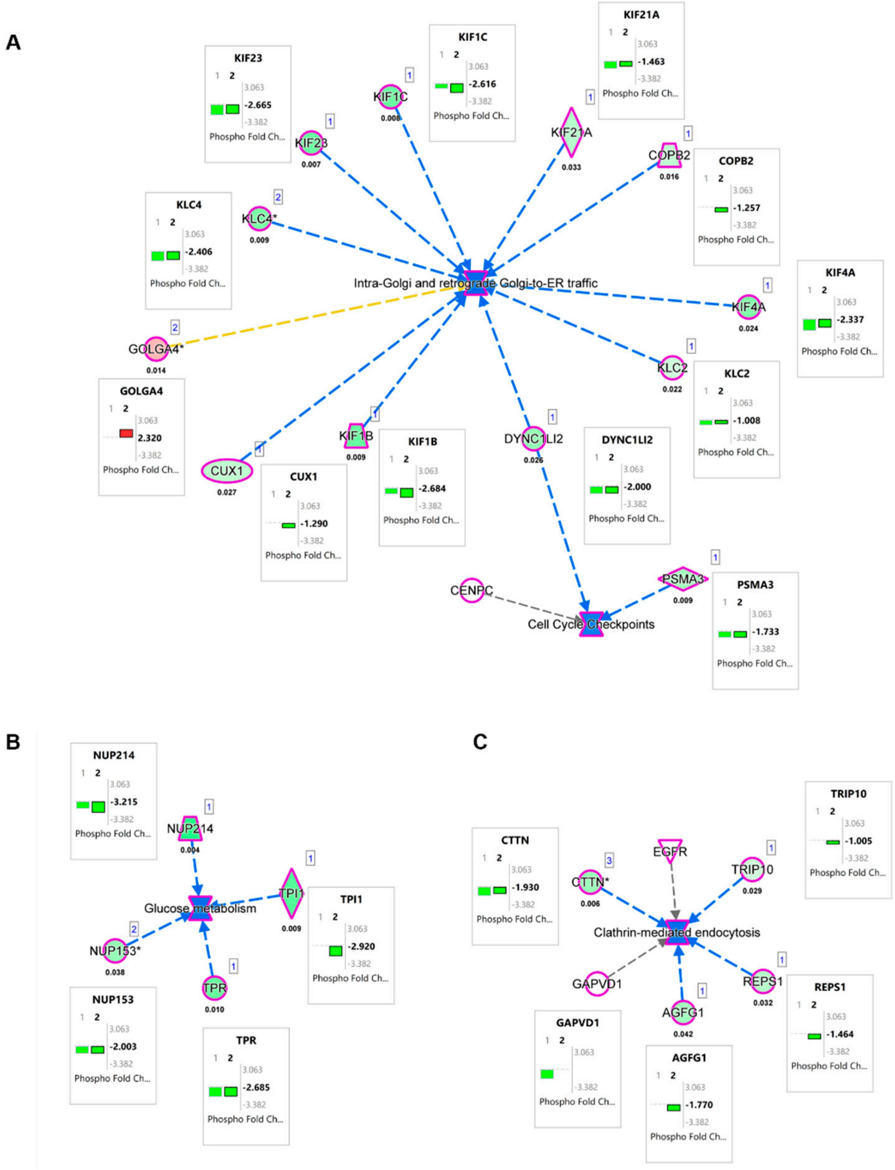

Figure S5. Molecular network analysis of canonical pathways deregulated in the NT (SC3KO vs. WT) group. Differentially expressed phosphoproteins/sites were mapped to key pathways. **(A)** Intra-Golgi and retrograde Golgi-to-ER traffic, **(B)** glucose metabolism, and **(C)** clathrin-mediated endocytosis. Molecular network analysis was performed using Ingenuity Pathway Analysis (IPA) version 24.0.1. Node color indicates relative expression (red: upregulated; green: downregulated; blue: predicted inhibition), and edges represent curated molecular relationships from the IPA knowledgebase.

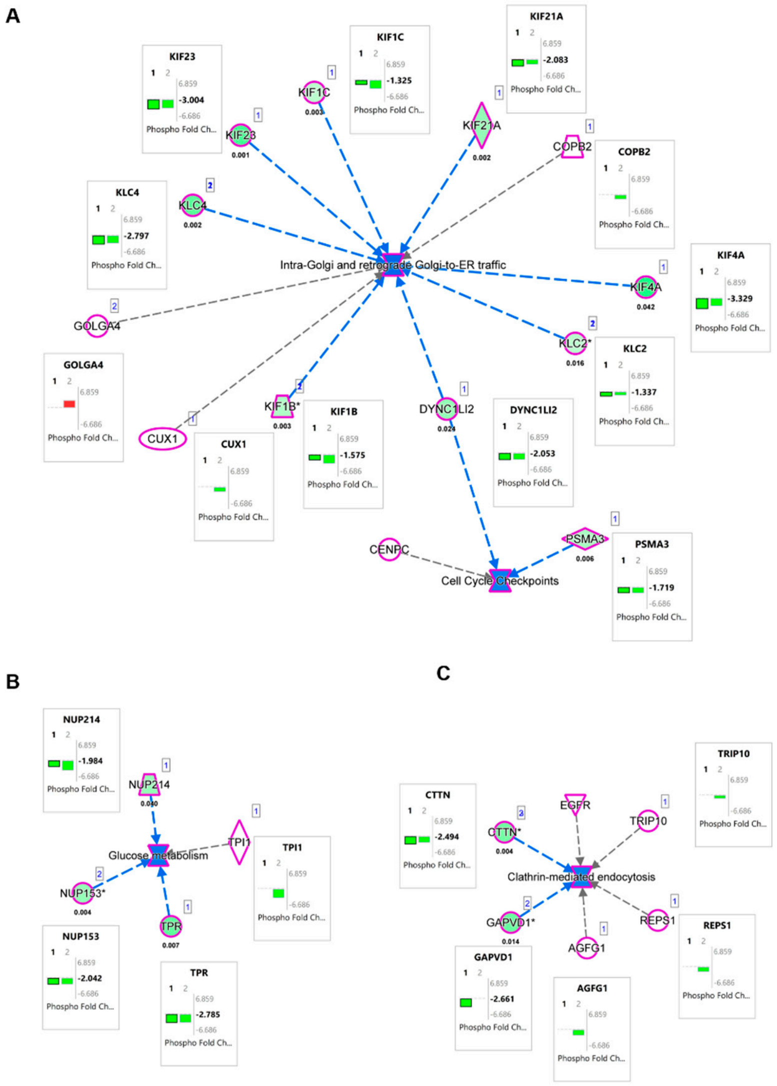

Figure S6. Molecular network analysis of canonical pathways deregulated in the MK (SC3KO vs. WT) group. Comparative analyses were performed on SUM-149 WT and SC3KO treated with 8 μM MK8353 treatment (MK: SC3KO vs. WT). Differentially expressed phosphoproteins/sites were mapped to key pathways. **(A)** Intra-Golgi and retrograde Golgi-to-ER traffic, **(B)** glucose metabolism, and **(C)** clathrin-mediated endocytosis. Molecular network analysis was performed using Ingenuity Pathway Analysis (IPA) version 24.0.1. Node color indicates relative expression (red: upregulated; green: downregulated; blue: predicted inhibition), and edges represent curated molecular relationships from the IPA knowledgebase.

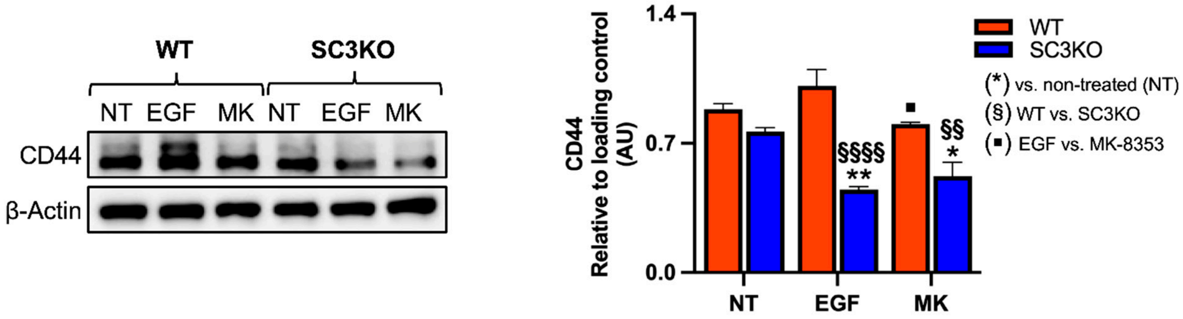

Figure S7. Evaluation of CD44 Expression. Representative immunoblot of total protein lysates from wild-type (WT) and SCAMP3 knockout (SC3KO) TNBC SUM-149 cells. Cells were either left untreated (NT), stimulated with EGF (10 ng/mL EGF for 30 min), or treated with 8 μM MK-8353 (MK) for 2h. Blots were probed for CD44. β-actin served as a loading control. Densitometric quantification of CD44 levels normalized to the loading control from three independent experiments. Band intensities were measured using ImageJ software. Data are presented as mean ± SEM (n=3). Statistical significance was determined by two-way ANOVA with Tukey's post-hoc test. (*) indicates significance relative to the untreated (NT) control within each genotype (*p≤0.05, **p<0.01), and (§) indicates significance between WT and SC3KO groups for the same treatment condition (§§p<0.01).
