# Supplementary material for: SCAMP3-Driven Regulation of ERK1/2 and Autophagy Phosphoproteomics Signatures in Triple-Negative Breast Cancer
Source: Int J Mol Sci. 2025 Oct 1;26(19):9577. doi: 10.3390/ijms26199577 (PMC12525412; doi:10.3390/ijms26199577)
Supplement: Supplementary file 1 [file ijms-26-09577-s001.zip › Table S1.pdf]

Table S1: TMT-based quantitative proteomic analysis: Experimental and labeling scheme.

TMT 1			
**TMT Label**	**Sample ID**	**Tube Label**	**Category**
126	N1 WT p13 (29-Oct-22) NT	1	WT Not Treated
127N	N1 WT p13 (29-Oct-22) EGF	2	WT 10ng/mL EGF
127C	N1 WT p13 (29-Oct-22) MK	3	WT 8uM MK-8353
128N	N1 SC3KO p14 (14-Sep-22) NT	4	SC3KO Not Treated
128C	N1 SC3KO p14 (14-Sep-22) EGF	5	SC3KO 10ng/mL EGF
129N	N1 SC3KO p14 (14-Sep-22) MK-8353	6	SC3KO8uM MK-8353
129C	N4 WT p16 (9-Nov-22) NT	19	WT Not Treated
130N	N4 WT p16 (9-Nov-22) EGF	20	WT 10ng/mL EGF
130C	Pool (n=4) WT NT, aliquot 1		Internal Control
131N	N/A	N/A	N/A
131C	N/A	N/A	N/A
**TMT 2**			
126	N4 WT p16 (9-Nov-22) MK-8353	21	WT Not Treated
127N	N4 SC3-KO p12 (18-Oct-22) NT	22	SC3KO Not Treated
127C	N2 WT p15 (2-Nov-22) NT	7	WT Basal
128N	N2 WT p15 (2-Nov-22) EGF	8	WT 10ng/mL EGF
128C	N2 WT p15 (2-Nov-22) MK-8353	9	WT 8uM MK-8353
129N	N2 SC3KO p12 (5-Oct-22) NT	10	SC3KO Not Treated
129C	N2 SC3KO p12 (5-Oct-22) EGF	11	SC3KO 10ng/mL EGF
130N	N2 SC3KO p12 (5-Oct-22) MK-8353	12	SC3KO 8uM MK-8353
130C	Pool (n=4) WT NT, aliquot 2		Internal Control
131N	N/A	N/A	N/A
131C	N/A	N/A	N/A
**TMT 3**			
126	N3 WT p14 (4-Nov-22) NT	13	WT Not Treated
127N	N3 WT p14 (4-Nov-22) EGF	14	WT10ng/mL EGF
127C	N3 WT p14 (4-Nov-22) MK-8353	15	WT 8uM MK-8353
128N	N3 SC3KO p13 (11-Oct-22) NT	16	SC3KO Not Treated
128C	N3 SC3KO p13 (11-Oct-22) EGF	17	SC3KO 10ng/mL EGF
129N	N3 SC3KO p13 (11-Oct-22) MK-8353	18	SC3KO 8uM MK-8353
129C	N4 SC3KO (18-Oct-22) EGF	23	SC3KO10ng/mL EGF
130N	N4 SC3KO (18-Oct-22) MK-8353	24	SC3KO 8uM MK-8353
130C	Pool (n=4) WT NT, aliquot 3		Internal Control
131N	N/A	N/A	N/A
131C	N/A	N/A	N/A

[1]All analyses were conducted using human protein extracts, with each sample containing 1mg total protein (n=24).
