# Supplementary material for: SCAMP3-Driven Regulation of ERK1/2 and Autophagy Phosphoproteomics Signatures in Triple-Negative Breast Cancer
Source: Int J Mol Sci. 2025 Oct 1;26(19):9577. doi: 10.3390/ijms26199577 (PMC12525412; doi:10.3390/ijms26199577)
Supplement: Supplementary file 1 [file ijms-26-09577-s001.zip › Table S2.pdf]

Table S2: Proteomic data showing phosphorylated peptides detected in TNBC cells.

**WT: MK vs. NT**				
**Accesion**	**Gene**	**Name**	**Phosphosite**	**log2(FC)**
Q09666	AHNAK	Neuroblast differentiation-associated protein AHNAK [OS=Homo sapiens]	S41	-6.01
Q6PKG0	LARP1	La-related protein 1 [OS=Homo sapiens]	S143	-5.76
Q8TF01	PNISR	Arginine/serine-rich protein PNISR [OS=Homo sapiens]	S211	-5.68
Q9UJU6	DBNL	Drebrin-like protein [OS=Homo sapiens]	S232	-5.64
P38159	RBMX	RNA-binding motif protein, X chromosome [OS=Homo sapiens]	[318-339]	-5.63
Q5T200	ZC3H13	Zinc finger CCCH domain-containing protein 13 [OS=Homo sapiens]	S207	-5.59
Q03164	KMT2A	Histone-lysine N-methyltransferase 2A [OS=Homo sapiens]	S3036	-5.59
Q6WKZ4	RAB11FIP1	Rab11 family-interacting protein 1 [OS=Homo sapiens]	S435	-5.53
P38159	RBMX	RNA-binding motif protein, X chromosome [OS=Homo sapiens]	[325-339]	-5.53
Q5T0W9	FAM83B	Protein FAM83B [OS=Homo sapiens]	S388	-5.46
Q9NX40	OCIAD1	OCIA domain-containing protein 1 [OS=Homo sapiens]	[121-131]	-5.37
Q96JY6	PDLIM2	PDZ and LIM domain protein 2 [OS=Homo sapiens]	S197	-5.34
Q9UQ35	SRRM2	Serine/arginine repetitive matrix protein 2 [OS=Homo sapiens]	T2599	-5.25
Q14160	SCRIB	Protein scribble homolog [OS=Homo sapiens]	[1536-1574]	-5.21
Q9BST9	RTKN	Rhotekin [OS=Homo sapiens]	S543	-5.16
Q9UQ35	SRRM2	Serine/arginine repetitive matrix protein 2 [OS=Homo sapiens]	[987-998]	-4.93
Q14134	TRIM29	Tripartite motif-containing protein 29 [OS=Homo sapiens]	[483-492]	-4.93
Q5VTL8	PRPF38B	Pre-mRNA-splicing factor 38B [OS=Homo sapiens]	S527; S529	-4.75
Q8TDM6	DLG5	Disks large homolog 5 [OS=Homo sapiens]	S264	-4.72
P46013	MKI67	Proliferation marker protein Ki-67 [OS=Homo sapiens]	[1368-1388]	-4.62
Q9H1E3	NUCKS1	Nuclear ubiquitous casein and cyclin-dependent kinase substrate 1 [OS=Homo sapiens]	S229; S234; S240	-4.36
Q8IYB3	SRRM1	Serine/arginine repetitive matrix protein 1 [OS=Homo sapiens]	S450; S452	-3.69
O75367	MACROH2A1	Core histone macro-H2A.1 [OS=Homo sapiens]	T129 [122-134]	-3.35
P02545-2	LMNA	Isoform C of Prelamin-A/C [OS=Homo sapiens]	S390	-3.01
P02545	LMNA	Prelamin-A/C [OS=Homo sapiens]	S390	-3.00
Q8IYB3	SRRM1	Serine/arginine repetitive matrix protein 1 [OS=Homo sapiens]	S696	-2.98
Q9H1E3	NUCKS1	Nuclear ubiquitous casein and cyclin-dependent kinase substrate 1 [OS=Homo sapiens]	S214 [202-218]	-2.95
P06748-3	NPM1	Isoform 3 of Nucleophosmin [OS=Homo sapiens]	T199	-2.87
P06748	NPM1	Nucleophosmin [OS=Homo sapiens]	T199	-2.86
P27816	MAP4	Microtubule-associated protein 4 [OS=Homo sapiens]	T521	-2.83
Q8IUD2	ERC1	ELKS/Rab6-interacting/CAST family member 1 [OS=Homo sapiens]	S17	-2.81
Q9UQ35	SRRM2	Serine/arginine repetitive matrix protein 2 [OS=Homo sapiens]	S2581	-2.74
Q00587	CDC42EP1	Cdc42 effector protein 1 [OS=Homo sapiens]	S121	-2.71
Q9H7N4	SCAF1	Splicing factor, arginine/serine-rich 19 [OS=Homo sapiens]	S965	-2.69
Q9H4A3	WNK1	Serine/threonine-protein kinase WNK1 [OS=Homo sapiens]	S2372	-2.67
Q8IYB3	SRRM1	Serine/arginine repetitive matrix protein 1 [OS=Homo sapiens]	S562	-2.61
Q9UQ35	SRRM2	Serine/arginine repetitive matrix protein 2 [OS=Homo sapiens]	S2132	-2.58
Q09666	AHNAK	Neuroblast differentiation-associated protein AHNAK [OS=Homo sapiens]	S3426	-2.55

Q9NZN5	ARHGEF12	Rho guanine nucleotide exchange factor 12 [OS=Homo sapiens]	T736	-2.52
Q6WCQ1-2	MPRIP	Isoform 2 of Myosin phosphatase Rho-interacting protein [OS=Homo sapiens]	S619	-2.49
Q9NTI5	PDS5B	Sister chromatid cohesion protein PDS5 homolog B [OS=Homo sapiens]	S1166	-2.48
Q9UQN3	CHMP2B	Charged multivesicular body protein 2b [OS=Homo sapiens]	S199 [196-208]	-2.46
P20700	LMNB1	Lamin-B1 [OS=Homo sapiens]	S23	-2.44
Q96T58	SPEN	Msx2-interacting protein [OS=Homo sapiens]	S736; S740	-2.44
P16144	ITGB4	Integrin beta-4 [OS=Homo sapiens]	S1474	-2.43
Q5SSJ5	HP1BP3	Heterochromatin protein 1-binding protein 3 [OS=Homo sapiens]	S142	-2.39
O60231	DHX16	Pre-mRNA-splicing factor ATP-dependent RNA helicase DHX16 [OS=Homo sapiens]	S160	-2.37
Q8IYB3	SRRM1	Serine/arginine repetitive matrix protein 1 [OS=Homo sapiens]	S715	-2.37
P46013	MKI67	Proliferation marker protein Ki-67 [OS=Homo sapiens]	S584	-2.36
Q14160	SCRIB	Protein scribble homolog [OS=Homo sapiens]	S1486	-2.34
P19338	NCL	Nucleolin [OS=Homo sapiens]	T76	-2.33
Q15629	TRAM1	Translocating chain-associated membrane protein 1 [OS=Homo sapiens]	S365	-2.33
*Q9BUQ8	DDX23	Probable ATP-dependent RNA helicase DDX23 [OS=Homo sapiens]	S14	-2.32
Q9NYV4	CDK12	Cyclin-dependent kinase 12 [OS=Homo sapiens]	[253-271]	-2.30
Q9BXP5	SRRT	Serrate RNA effector molecule homolog [OS=Homo sapiens]	S67; S74	-2.27
P67870	CSNK2B	Casein kinase II subunit beta [OS=Homo sapiens]	[S/T/Y] [192-215]	-2.2429
Q8NI27	THOC2	THO complex subunit 2 [OS=Homo sapiens]	[1438-1453]	-2.14
P46937	YAP1	Transcriptional coactivator YAP1 [OS=Homo sapiens]	S367	-2.14
P35658	NUP214	Nuclear pore complex protein Nup214 [OS=Homo sapiens]	S646	-2.13
Q92922	SMARCC1	SWI/SNF complex subunit SMARCC1 [OS=Homo sapiens]	S328; S330	-2.12
Q9NR30	DDX21	Nucleolar RNA helicase 2 [OS=Homo sapiens]	S121 [117-132]	-2.11
Q15424	SAFB	Scaffold attachment factor B1 [OS=Homo sapiens]	S383	-2.11
P27824	CANX	Calnexin [OS=Homo sapiens]	S554	-2.09
Q07157	TJP1	Tight junction protein ZO-1 [OS=Homo sapiens]	S617	-2.09
Q07157-2	TJP1	Isoform Short of Tight junction protein ZO-1 [OS=Homo sapiens]	S617	-2.08
Q9UQ35	SRRM2	Serine/arginine repetitive matrix protein 2 [OS=Homo sapiens]	T1531	-2.06
Q53GS9	USP39	U4/U6.U5 tri-snRNP-associated protein 2 [OS=Homo sapiens]	S82	-2.06
Q9H7N4	SCAF1	Splicing factor, arginine/serine-rich 19 [OS=Homo sapiens]	S498; S500	-2.05
Q13442	PDAP1	28 kDa heat- and acid-stable phosphoprotein [OS=Homo sapiens]	S60; S63	-2.05
Q14C86	GAPVD1	GTPase-activating protein and VPS9 domain-containing protein 1 [OS=Homo sapiens]	S902	-2.02
*Q07955	SRSF1	Serine/arginine-rich splicing factor 1 [OS=Homo sapiens]	S199	-2.00
O14497	ARID1A	AT-rich interactive domain-containing protein 1A [OS=Homo sapiens]	S696	-1.99
Q9H910	JPT2	Jupiter microtubule associated homolog 2 [OS=Homo sapiens]	S97	-1.98
Q8N3D4	EHBP1L1	EH domain-binding protein 1-like protein 1 [OS=Homo sapiens]	S1257	-1.95

Q9ULH1	ASAP1	Arf-GAP with SH3 domain, ANK repeat and PH domain-containing protein 1 [OS=Homo sapiens]	S1027	-1.93
Q86SQ0	PHLDB2	Pleckstrin homology-like domain family B member 2 [OS=Homo sapiens]	S73	-1.93
Q13188	STK3	Serine/threonine-protein kinase 3 [OS=Homo sapiens]	[382-405]	-1.89
Q99590	SCAF11	Protein SCAF11 [OS=Homo sapiens]	S963	-1.88
Q09666	AHNAK	Neuroblast differentiation-associated protein AHNAK [OS=Homo sapiens]	S93 [90-102]	-1.86
Q96JP5	ZFP91	E3 ubiquitin-protein ligase ZFP91 [OS=Homo sapiens]	S103	-1.85
P27816	MAP4	Microtubule-associated protein 4 [OS=Homo sapiens]	S787	-1.79
Q01082	SPTBN1	Spectrin beta chain, non-erythrocytic 1 [OS=Homo sapiens]	S2358	-1.75
P32519	ELF1	ETS-related transcription factor Elf-1 [OS=Homo sapiens]	[160-175]	-1.64
Q92576	PHF3	PHD finger protein 3 [OS=Homo sapiens]	[116-127]	-1.63
P51858	HDGF	Hepatoma-derived growth factor [OS=Homo sapiens]	S165	-1.54
O75475	PSIP1	PC4 and SFRS1-interacting protein [OS=Homo sapiens]	T141	-1.50
O95785	WIZ	Protein Wiz [OS=Homo sapiens]	S983	-1.47
Q86X29	LSR	Lipolysis-stimulated lipoprotein receptor [OS=Homo sapiens]	T501	1.46
Q9C0C2	TNKS1BP1	182 kDa tankyrase-1-binding protein [OS=Homo sapiens]	[1532-1551]	1.47
P48634	PRRC2A	Protein PRRC2A [OS=Homo sapiens]	S1219	1.47
P16949	STMN1	Stathmin [OS=Homo sapiens]	S38	1.47
Q6PKG0	LARP1	La-related protein 1 [OS=Homo sapiens]	S165	1.47
Q9H1K1	ISCU	Iron-sulfur cluster assembly enzyme ISCU, mitochondrial [OS=Homo sapiens]	S29	1.48
Q07866-4	KLC1	Isoform J of Kinesin light chain 1 [OS=Homo sapiens]	S631	1.48
Q8N4C8	MINK1	Misshapen-like kinase 1 [OS=Homo sapiens]	[714-739]	1.48
Q09666	AHNAK	Neuroblast differentiation-associated protein AHNAK [OS=Homo sapiens]	S4986	1.48
Q14166	TTLL12	Tubulin--tyrosine ligase-like protein 12 [OS=Homo sapiens]	S16	1.48
P32519	ELF1	ETS-related transcription factor Elf-1 [OS=Homo sapiens]	S187	1.49
O75475	PSIP1	PC4 and SFRS1-interacting protein [OS=Homo sapiens]	S106	1.50
Q9UKV3	ACIN1	Apoptotic chromatin condensation inducer in the nucleus [OS=Homo sapiens]	S216	1.50
Q9BW71	HIRIP3	HIRA-interacting protein 3 [OS=Homo sapiens]	S227	1.50
Q13523	PRP4K	Serine/threonine-protein kinase PRP4 homolog [OS=Homo sapiens]	S166	1.50
Q8TAD8	SNIP1	Smad nuclear-interacting protein 1 [OS=Homo sapiens]	S52; S54	1.51
Q9UKJ3	GPATCH8	G patch domain-containing protein 8 [OS=Homo sapiens]	S1107	1.54
P26358	DNMT1	DNA (cytosine-5)-methyltransferase 1 [OS=Homo sapiens]	[141-156]	1.54
Q9NR30	DDX21	Nucleolar RNA helicase 2 [OS=Homo sapiens]	S121 [114-132]	1.55
O14686	EML3	Histone-lysine N-methyltransferase 2D [OS=Homo sapiens]	S2274	1.55
Q32P44	KMT2D	Echinoderm microtubule-associated protein-like 3 [OS=Homo sapiens]	S156	1.55
P46087	NOP2	Probable 28S rRNA (cytosine(4447)-C(5))-methyltransferase [OS=Homo sapiens]	S67	1.56
Q96TA1	NIBAN2	Protein Niban 2 [OS=Homo sapiens]	S665	1.56
P62826	RAN	GTP-binding nuclear protein Ran [OS=Homo sapiens]	S135	1.57
P49023	PXN	Paxillin [OS=Homo sapiens]	S321	1.57

P62753	RPS6	40S ribosomal protein S6 [OS=Homo sapiens]	S236; S240	1.57
Q9UQ35	SRRM2	Serine/arginine repetitive matrix protein 2 [OS=Homo sapiens]	S854; T866	1.57
Q9Y2W1	THRAP3	Thyroid hormone receptor-associated protein 3 [OS=Homo sapiens]	S243	1.58
Q9BZE4	GTPBP4	GTP-binding protein 4 [OS=Homo sapiens]	S558	1.58
Q70EL4	USP43	Ubiquitin carboxyl-terminal hydrolase 43 [OS=Homo sapiens]	S1111	1.59
Q9UKV3	ACIN1	Apoptotic chromatin condensation inducer in the nucleus [OS=Homo sapiens]	S16	1.59
Q09666	AHNAK	Neuroblast differentiation-associated protein AHNAK [OS=Homo sapiens]	S5752	1.59
Q6DT37	CDC42BPG	Serine/threonine-protein kinase MRCK gamma [OS=Homo sapiens]	S1482	1.59
Q96ST3	SIN3A	Paired amphipathic helix protein Sin3a [OS=Homo sapiens]	S1112	1.60
Q8NEY1	NAV1	Neuron navigator 1 [OS=Homo sapiens]	S672	1.61
P43307	SSR1	Translocon-associated protein subunit alpha [OS=Homo sapiens]	S268	1.61
Q14004	CDK13	Cyclin-dependent kinase 13 [OS=Homo sapiens]	T588	1.62
Q9P206	KIAA1522	Uncharacterized protein KIAA1522 [OS=Homo sapiens]	S545	1.62
Q9UQ35	SRRM2	Serine/arginine repetitive matrix protein 2 [OS=Homo sapiens]	S351; S353	1.63
P78347	GTF2I	General transcription factor II-I [OS=Homo sapiens]	[807-826]	1.64
P09651	HNRNPA1	Heterogeneous nuclear ribonucleoprotein A1 [OS=Homo sapiens]	S6	1.64
Q9UQB8	BAIAP2	Brain-specific angiogenesis inhibitor 1-associated protein 2 [OS=Homo sapiens]	T360	1.64
O95785	WIZ	Protein Wiz [OS=Homo sapiens]	S1017	1.65
Q13177	PAK2	Serine/threonine-protein kinase PAK 2 [OS=Homo sapiens]	S141	1.66
O75676	RPS6KA4	Ribosomal protein S6 kinase alpha-4 [OS=Homo sapiens]	S682	1.66
Q9UDY2	TJP2	Tight junction protein ZO-2 [OS=Homo sapiens]	S986	1.69
P35611	ADD1	Alpha-adducin [OS=Homo sapiens]	S726	1.71
Q9UEY8	ADD3	Gamma-adducin [OS=Homo sapiens]	S693	1.71
Q5T0W9	FAM83B	Protein FAM83B [OS=Homo sapiens]	S764	1.72
*P18615	NELFE	Negative elongation factor E [OS=Homo sapiens]	S181	1.72
Q9Y2W1	THRAP3	Thyroid hormone receptor-associated protein 3 [OS=Homo sapiens]	S698	1.72
P10644	PRKAR1A	cAMP-dependent protein kinase type I-alpha regulatory subunit [OS=Homo sapiens]	S77; S83	1.73
O14497	ARID1A	AT-rich interactive domain-containing protein 1A [OS=Homo sapiens]	S1600	1.74
Q6Y7W6	GIGYF2	GRB10-interacting GYF protein 2 [OS=Homo sapiens]	T382	1.74
Q9H0B6	KLC2	Kinesin light chain 2 [OS=Homo sapiens]	[506-518]	1.76
*Q9BUQ8	DDX23	Probable ATP-dependent RNA helicase DDX23 [OS=Homo sapiens]	S109	1.77
P48634	PRRC2A	Protein PRRC2A [OS=Homo sapiens]	T1347	1.77
Q9H1E3	NUCKS1	Nuclear ubiquitous casein and cyclin-dependent kinase substrate 1 [OS=Homo sapiens]	S214 [202-219]	1.79
Q09666	AHNAK	Neuroblast differentiation-associated protein AHNAK [OS=Homo sapiens]	[5748-5772]	1.80
Q13330	MTA1	Metastasis-associated protein MTA1 [OS=Homo sapiens]	S576	1.81

*Q13263	TRIM28	Transcription intermediary factor 1-beta [OS=Homo sapiens]	S473	1.81
Q7KZI7-14	MARK2	Isoform 14 of Serine/threonine-protein kinase MARK2 [OS=Homo sapiens]	[413-429]	1.82
Q2M2I8	AAK1	AP2-associated protein kinase 1 [OS=Homo sapiens]	S678	1.83
Q96QC0	PPP1R10	Serine/threonine-protein phosphatase 1 regulatory subunit 10 [OS=Homo sapiens]	S313	1.84
O94979	SEC31A	Protein transport protein Sec31A [OS=Homo sapiens]	S799	1.85
Q04637	EIF4G1	Eukaryotic translation initiation factor 4 gamma 1 [OS=Homo sapiens]	S1092	1.85
P23588	EIF4B	Eukaryotic translation initiation factor 4B [OS=Homo sapiens]	S597	1.85
Q13428-6	TCOF1	Isoform 6 of Treacle protein [OS=Homo sapiens]	T249	1.85
Q13428-7	TCOF1	Isoform 7 of Treacle protein [OS=Homo sapiens]	T249	1.86
Q92615	LARP4B	La-related protein 4B [OS=Homo sapiens]	S524	1.86
Q13428-3	TCOF1	Isoform 3 of Treacle protein [OS=Homo sapiens]	T249	1.87
*Q9HCD5	NCOA5	Nuclear receptor coactivator 5 [OS=Homo sapiens]	S126	1.89
P06730	EIF4E	Eukaryotic translation initiation factor 4E [OS=Homo sapiens]	[193-212]	1.89
P35658	NUP214	Nuclear pore complex protein Nup214 [OS=Homo sapiens]	S433	1.92
P35658	NUP214	Nuclear pore complex protein Nup214 [OS=Homo sapiens]	T434	1.92
Q92576	PHF3	PHD finger protein 3 [OS=Homo sapiens]	S1133	1.92
P46937	YAP1	Transcriptional coactivator YAP1 [OS=Homo sapiens]	S105; S109	1.92
Q9NR19	ACSS2	Acetyl-coenzyme A synthetase, cytoplasmic [OS=Homo sapiens]	S267	1.92
P49454	CENPF	Centromere protein F [OS=Homo sapiens]	[2988-3003]	1.93
Q8IUD2	ERC1	ELKS/Rab6-interacting/CAST family member 1 [OS=Homo sapiens]	S191	1.94
P06748	NPM1	Nucleophosmin [OS=Homo sapiens]	T219	1.94
P06748-3	NPM1	Isoform 3 of Nucleophosmin [OS=Homo sapiens]	T219	1.95
Q03164	KMT2A	Histone-lysine N-methyltransferase 2A [OS=Homo sapiens]	S3527	1.95
Q9H6Z4	RANBP3	Ran-binding protein 3 [OS=Homo sapiens]	S100; S101	1.95
Q8N3D4	EHBP1L1	EH domain-binding protein 1-like protein 1 [OS=Homo sapiens]	S1273	1.99
Q6ZRV2	FAM83H	Protein FAM83H [OS=Homo sapiens]	S998	2.00
Q86X27	RALGPS2	Ras-specific guanine nucleotide-releasing factor RalGPS2 [OS=Homo sapiens]	S329	2.01
Q8NFJ5	GPRC5A	Retinoic acid-induced protein 3 [OS=Homo sapiens]	S345	2.01
Q9NYF8	BCLAF1	Bcl-2-associated transcription factor 1 [OS=Homo sapiens]	S177	2.01
Q9NYF8-4	BCLAF1	Isoform 4 of Bcl-2-associated transcription factor 1 [OS=Homo sapiens]	S177	2.02
Q96K21	ZFYVE19	Abscission/NoCut checkpoint regulator [OS=Homo sapiens]	S144	2.02
Q14160	SCRIB	Protein scribble homolog [OS=Homo sapiens]	S1348	2.03
O60333	KIF1B	Kinesin-like protein KIF1B [OS=Homo sapiens]	S1454	2.03
P08651	NFIC	Nuclear factor 1 C-type [OS=Homo sapiens]	S323	2.03
Q9H6H4	REEP4	Receptor expression-enhancing protein 4 [OS=Homo sapiens]	S152	2.04
Q9H0D6	XRN2	5'-3' exoribonuclease 2 [OS=Homo sapiens]	S448	2.05
Q8IYB3	SRRM1	Serine/arginine repetitive matrix protein 1 [OS=Homo sapiens]	S260	2.06
Q9H4A3	WNK1	Serine/threonine-protein kinase WNK1 [OS=Homo sapiens]	[T/S] [366-381]	2.06
Q8WXE1	ATRIP	ATR-interacting protein [OS=Homo sapiens]	S224	2.07
Q8IZP0	ABI1	Abl interactor 1 [OS=Homo sapiens]	S225	2.07
Q9UQ35	SRRM2	Serine/arginine repetitive matrix protein 2 [OS=Homo sapiens]	S994; T983	2.09

P48634	PRRC2A	Protein PRRC2A [OS=Homo sapiens]	S1147	2.10
P28749	RBL1	Retinoblastoma-like protein 1 [OS=Homo sapiens]	S640	2.10
P29350	PTPN6	Tyrosine-protein phosphatase non-receptor type 6 [OS=Homo sapiens]	[555-570]	2.11
Q9UQ35	SRRM2	Serine/arginine repetitive matrix protein 2 [OS=Homo sapiens]	S1179	2.11
Q8IZ21	PHACTR4	Phosphatase and actin regulator 4 [OS=Homo sapiens]	S590	2.12
Q9P107	GMIP	GEM-interacting protein [OS=Homo sapiens]	S437	2.13
Q9ULJ3	ZBTB21	Zinc finger and BTB domain-containing protein 21 [OS=Homo sapiens]	S411; S422	2.14
Q14155-1	ARHGEF7	Isoform 1 of Rho guanine nucleotide exchange factor 7 [OS=Homo sapiens]	S340	2.14
Q04637	EIF4G1	Eukaryotic translation initiation factor 4 gamma 1 [OS=Homo sapiens]	S1231	2.14
Q14980	NUMA1	Nuclear mitotic apparatus protein 1 [OS=Homo sapiens]	S1757	2.14
Q9C0C2	TNKS1BP1	182 kDa tankyrase-1-binding protein [OS=Homo sapiens]	S672	2.16
P19338	NCL	Nucleolin [OS=Homo sapiens]	S67	2.17
P67870	CSNK2B	Casein kinase II subunit beta [OS=Homo sapiens]	S209	2.20
Q96CV9	OPTN	Optineurin [OS=Homo sapiens]	[525-537]	2.21
Q15054	POLD3	DNA polymerase delta subunit 3 [OS=Homo sapiens]	S307	2.22
Q14C86	GAPVD1	GTPase-activating protein and VPS9 domain-containing protein 1 [OS=Homo sapiens]	S914	2.22
Q8IXM2	BAP18	Chromatin complexes subunit BAP18 [OS=Homo sapiens]	S96	2.22
P02545	LMNA	Prelamin-A/C [OS=Homo sapiens]	S18	2.24
P02545-2	LMNA	Isoform C of Prelamin-A/C [OS=Homo sapiens]	S18	2.24
P02545	LMNA	Prelamin-A/C [OS=Homo sapiens]	T19	2.25
Q8IYB3	SRRM1	Serine/arginine repetitive matrix protein 1 [OS=Homo sapiens]	S675	2.25
P02545-2	LMNA	Isoform C of Prelamin-A/C [OS=Homo sapiens]	T19	2.26
Q6ZRS2	SRCAP	Helicase SRCAP [OS=Homo sapiens]	S1859	2.26
Q9UKV3	ACIN1	Apoptotic chromatin condensation inducer in the nucleus [OS=Homo sapiens]	S838	2.28
Q9Y520	PRRC2C	Protein PRRC2C [OS=Homo sapiens]	S2105	2.29
P46013	MKI67	Proliferation marker protein Ki-67 [OS=Homo sapiens]	S1679	2.30
P62750	RPL23A	60S ribosomal protein L23a [OS=Homo sapiens]	S43	2.31
P46013	MKI67	Proliferation marker protein Ki-67 [OS=Homo sapiens]	S1071	2.31
Q7Z309-3	PABIR2	Isoform 3 of PABIR family member 2 [OS=Homo sapiens]	S25	2.32
P23396	RPS3	40S ribosomal protein S3 [OS=Homo sapiens]	T221	2.33
Q9UQ35	SRRM2	Serine/arginine repetitive matrix protein 2 [OS=Homo sapiens]	[304-329]	2.34
Q96T58	SPEN	Msx2-interacting protein [OS=Homo sapiens]	S1287	2.35
Q14814	MEF2D	Myocyte-specific enhancer factor 2D [OS=Homo sapiens]	S180	2.36
Q96E09	PABIR1	PPP2R1A-PPP2R2A-interacting phosphatase regulator 1 [OS=Homo sapiens]	S76	2.38
Q99590	SCAF11	Protein SCAF11 [OS=Homo sapiens]	S796	2.39
Q8ND76	CCNY	Cyclin-Y [OS=Homo sapiens]	S326	2.39
Q13442	PDAP1	28 kDa heat- and acid-stable phosphoprotein [OS=Homo sapiens]	S60	2.39
Q96JY6	PDLIM2	PDZ and LIM domain protein 2 [OS=Homo sapiens]	S123; S129	2.42
P35222	CTNNB1	Catenin beta-1 [OS=Homo sapiens]	S552	2.45

O43166	SIPA1L1	Signal-induced proliferation-associated 1-like protein 1 [OS=Homo sapiens]	[205-227]	2.45
Q9BQG0	MYBBP1A	Myb-binding protein 1A [OS=Homo sapiens]	[1156-1169]	2.48
Q8IY67-2	RAVER1	Isoform 2 of Ribonucleoprotein PTB-binding 1 [OS=Homo sapiens]	T463	2.48
Q8TF01	PNISR	Arginine/serine-rich protein PNISR [OS=Homo sapiens]	[286-307]	2.50
Q8NDT2	RBM15B	Putative RNA-binding protein 15B [OS=Homo sapiens]	S562	2.50
P40818	USP8	Ubiquitin carboxyl-terminal hydrolase 8 [OS=Homo sapiens]	S718	2.52
Q6IBW4	NCAPH2	Condensin-2 complex subunit H2 [OS=Homo sapiens]	[275-293]	2.52
Q13442	PDAP1	28 kDa heat- and acid-stable phosphoprotein [OS=Homo sapiens]	S63	2.53
O60841	EIF5B	Eukaryotic translation initiation factor 5B [OS=Homo sapiens]	S214	2.54
P16144	ITGB4	Integrin beta-4 [OS=Homo sapiens]	T1530	2.55
P36507	MAP2K2	Dual specificity mitogen-activated protein kinase kinase 2 [OS=Homo sapiens]	T394	2.55
Q92597	NDRG1	Protein NDRG1 [OS=Homo sapiens]	S330; S333; S336	2.57
Q9Y2D5	PALM2AKAP2	A-kinase anchor protein 2 [OS=Homo sapiens]	S393	2.57
O43896	KIF1C	Kinesin-like protein KIF1C [OS=Homo sapiens]	T1083	2.58
P12270	TPR	Nucleoprotein TPR [OS=Homo sapiens]	S2155	2.58
Q9UQ35	SRRM2	Serine/arginine repetitive matrix protein 2 [OS=Homo sapiens]	S2030; S2032; T2034	2.60
Q8WWI1	LMO7	LIM domain only protein 7 [OS=Homo sapiens]	S116 [111-130]	2.60
*Q07955	SRSF1	Serine/arginine-rich splicing factor 1 [OS=Homo sapiens]	S238	2.61
Q7Z417	NUFIP2	FMR1-interacting nuclear fragile X mental retardation-interacting protein 2 [OS=Homo sapiens]	[564-581]	2.63
P46060	RANGAP1	Ran GTPase-activating protein 1 [OS=Homo sapiens]	S442	2.64
Q9NTI5	PDS5B	Sister chromatid cohesion protein PDS5 homolog B [OS=Homo sapiens]	S1358	2.65
P38159	RBMX	RNA-binding motif protein, X chromosome [OS=Homo sapiens]	S326	2.67
Q9ULH1	ASAP1	Arf-GAP with SH3 domain, ANK repeat and PH domain-containing protein 1 [OS=Homo sapiens]	S1008	2.73
Q5T200	ZC3H13	Zinc finger CCCH domain-containing protein 13 [OS=Homo sapiens]	S325	2.78
O15231-3	ZNF185	Isoform 3 of Zinc finger protein 185 [OS=Homo sapiens]	S520; T506	2.78
Q00587	CDC42EP1	Cdc42 effector protein 1 [OS=Homo sapiens]	S192	2.80
P04792	HSPB1	Heat shock protein beta-1 [OS=Homo sapiens]	S15	2.87
Q9BXP5	SRRT	Serrate RNA effector molecule homolog [OS=Homo sapiens]	T544	2.88
Q5VT52	RPRD2	Regulation of nuclear pre-mRNA domain-containing protein 2 [OS=Homo sapiens]	T723	2.92
O60231	DHX16	Pre-mRNA-splicing factor ATP-dependent RNA helicase DHX16 [OS=Homo sapiens]	S103; S106	3.02
Q86UU0	BCL9L	B-cell CLL/lymphoma 9-like protein [OS=Homo sapiens]	S21	3.09
O75367	MACROH2A1	Core histone macro-H2A.1 [OS=Homo sapiens]	T129 [122-135]	3.11
Q9Y520	PRRC2C	Protein PRRC2C [OS=Homo sapiens]	T2673	3.21
Q9Y2W1	THRAP3	Thyroid hormone receptor-associated protein 3 [OS=Homo sapiens]	[182-215]	3.21
Q8N1F8	STK11IP	Serine/threonine-protein kinase 11-interacting protein [OS=Homo sapiens]	[770-782]	3.28

P67809	YBX1	Y-box-binding protein 1 [OS=Homo sapiens]	S165	3.32
Q9UQN3	CHMP2B	Charged multivesicular body protein 2b [OS=Homo sapiens]	S199 [196-205]	3.32
Q9Y580	RBM7	RNA-binding protein 7 [OS=Homo sapiens]	S204	3.57
P02545	LMNA	Prelamin-A/C [OS=Homo sapiens]	S458	3.95
P02545-2	LMNA	Isoform C of Prelamin-A/C [OS=Homo sapiens]	S458	3.96
P49585	PCYT1A	Choline-phosphate cytidylyltransferase A [OS=Homo sapiens]	S362	3.99
Q8TB72	PUM2	Pumilio homolog 2 [OS=Homo sapiens]	S589	4.63
Q9H1E3	NUCKS1	Nuclear ubiquitous casein and cyclin-dependent kinase substrate 1 [OS=Homo sapiens]	S19	4.69
Q6WKZ4	RAB11FIP1	Rab11 family-interacting protein 1 [OS=Homo sapiens]	[433-443]	5.19
Q09666	AHNAK	Neuroblast differentiation-associated protein AHNAK [OS=Homo sapiens]	S93 [89-102]	5.33
Q6PKG0	LARP1	La-related protein 1 [OS=Homo sapiens]	[1054-1083]	5.34
Q9NX40	OCIAD1	OCIA domain-containing protein 1 [OS=Homo sapiens]	S108	5.38
Q9ULM3	YEATS2	YEATS domain-containing protein 2 [OS=Homo sapiens]	[524-540]	5.42
Q9BST9	RTKN	Rhotekin [OS=Homo sapiens]	S106	5.46
Q04637	EIF4G1	Eukaryotic translation initiation factor 4 gamma 1 [OS=Homo sapiens]	S1209	5.48
Q14160	SCRIB	Protein scribble homolog [OS=Homo sapiens]	S1378	5.49
Q9UJU6	DBNL	Drebrin-like protein [OS=Homo sapiens]	S269	5.51
Q8IYB3	SRRM1	Serine/arginine repetitive matrix protein 1 [OS=Homo sapiens]	S740	5.53
Q8IYB3	SRRM1	Serine/arginine repetitive matrix protein 1 [OS=Homo sapiens]	S738	5.53
Q8WWI1	LMO7	LIM domain only protein 7 [OS=Homo sapiens]	S116 [112-130]	5.56
Q13541	EIF4EBP1	Eukaryotic translation initiation factor 4E-binding protein 1 [OS=Homo sapiens]	S65	5.57
Q7L7X3	TAOK1	Serine/threonine-protein kinase TAO1 [OS=Homo sapiens]	S974	5.62
Q13029	PRDM2	PR domain zinc finger protein 2 [OS=Homo sapiens]	S643	5.64

**SC3KO: MK vs. NT**				
**Accesion**	**Gene**	**Name**	**Phosphosite**	**log2(FC)**
P32004	L1CAM	Neural cell adhesion molecule L1 [OS=Homo sapiens]	T1247	-1.79
P67870	CSNK2B	Casein kinase II subunit beta [OS=Homo sapiens]	S209	-1.75
Q02241-2	KIF23	Isoform 2 of Kinesin-like protein KIF23 [OS=Homo sapiens]	S807	-1.73
Q6WCQ1-2	MPRIP	Isoform 2 of Myosin phosphatase Rho-interacting protein [OS=Homo sapiens]	S1016	-1.72
Q5T200	ZC3H13	Zinc finger CCCH domain-containing protein 13 [OS=Homo sapiens]	S325	-1.64
Q96E09	PABIR1	PPP2R1A-PPP2R2A-interacting phosphatase regulator 1 [OS=Homo sapiens]	S76	-1.62
P49585	PCYT1A	Choline-phosphate cytidylyltransferase A [OS=Homo sapiens]	S362	-1.58
Q9BQG0	MYBBP1A	Myb-binding protein 1A [OS=Homo sapiens]	[1152-1167]	1.46
Q13428-3	TCOF1	Isoform 3 of Treacle protein [OS=Homo sapiens]	S381	1.49
Q13428-6	TCOF1	Isoform 6 of Treacle protein [OS=Homo sapiens]	S381	1.51
Q13428-7	TCOF1	Isoform 7 of Treacle protein [OS=Homo sapiens]	S381	1.50
O15234	CASC3	Protein CASC3 [OS=Homo sapiens]	S148	1.56
*Q13501	SQSTM1	Sequestosome-1 [OS=Homo sapiens]	S272	1.58
Q9UK58	CCNL1	Cyclin-L1 [OS=Homo sapiens]	S352	1.59

O60832	DKC1	H/ACA ribonucleoprotein complex subunit DKC1 [OS=Homo sapiens]	S494	1.62
Q9NYB9	ABI2	Abl interactor 2 [OS=Homo sapiens]	S183	1.65
Q9Y618	NUCKS1	Nuclear receptor corepressor 2 [OS=Homo sapiens]	S956	1.68
Q9H1E3	NCOR2	Nuclear ubiquitous casein and cyclin-dependent kinase substrate 1 [OS=Homo sapiens]	S54	1.68
O43237	DYNC1LI2	Cytoplasmic dynein 1 light intermediate chain 2 [OS=Homo sapiens]	S391	1.68
Q92615	LARP4B	La-related protein 4B [OS=Homo sapiens]	S731; S736	1.70
P29590-2	PML	Isoform PML-5 of Protein PML [OS=Homo sapiens]	S518; S527	1.74
Q96JP5	ZFP91	E3 ubiquitin-protein ligase ZFP91 [OS=Homo sapiens]	S103	1.76
Q01082	SPTBN1	Spectrin beta chain, non-erythrocytic 1 [OS=Homo sapiens]	S2161; S2165	1.86
Q8NFJ5	GPRC5A	Retinoic acid-induced protein 3 [OS=Homo sapiens]	S345	1.87
P46013	MKI67	Proliferation marker protein Ki-67 [OS=Homo sapiens]	S357	1.87
Q96E09	PABIR1	PPP2R1A-PPP2R2A-interacting phosphatase regulator 1 [OS=Homo sapiens]	S37	1.91
P46013	MKI67	Proliferation marker protein Ki-67 [OS=Homo sapiens]	[1218-1245]	1.96
Q9ULJ3	ZBTB21	Zinc finger and BTB domain-containing protein 21 [OS=Homo sapiens]	S1003	2.00
Q05519	SRSF11	Serine/arginine-rich splicing factor 11 [OS=Homo sapiens]	S434	2.03
Q03188	CENPC	Centromere protein C [OS=Homo sapiens]	[331-344]	2.08
Q6ZRV2	FAM83H	Protein FAM83H [OS=Homo sapiens]	[508-520]	2.12
P48634	PRRC2A	Protein PRRC2A [OS=Homo sapiens]	S1219	2.15
Q14C86	GAPVD1	GTPase-activating protein and VPS9 domain-containing protein 1 [OS=Homo sapiens]	S914	2.16
A0MZ66	SHTN1	Shootin-1 [OS=Homo sapiens]	S534	2.20
P38159	RBMX	RNA-binding motif protein, X chromosome [OS=Homo sapiens]	[325-339]	2.21
Q14814	MEF2D	Myocyte-specific enhancer factor 2D [OS=Homo sapiens]	S231	2.23
Q7Z417	NUFIP2	Nuclear fragile X mental retardation-interacting protein 2 [OS=Homo sapiens]	S112	2.28
P12270	TPR	Nucleoprotein TPR [OS=Homo sapiens]	[644-669]	2.30
Q96JY6	PDLIM2	PDZ and LIM domain protein 2 [OS=Homo sapiens]	S197	2.30
Q14980	NUMA1	Nuclear mitotic apparatus protein 1 [OS=Homo sapiens]	S1969	2.32
P24928	POLR2A	DNA-directed RNA polymerase II subunit RPB1 [OS=Homo sapiens]	S1913; S1920; S1927	2.34
P24928	POLR2A	DNA-directed RNA polymerase II subunit RPB1 [OS=Homo sapiens]	S1934	2.35
Q9NSK0	KLC4	Kinesin light chain 4 [OS=Homo sapiens]	S611	2.34
O95239	KIF4A	Chromosome-associated kinesin KIF4A [OS=Homo sapiens]	S801	2.38
Q9Y6M7-7	SLC4A7	Isoform 7 of Sodium bicarbonate cotransporter 3 [OS=Homo sapiens]	S242	2.38
Q6WCQ1-2	MPRIP	Isoform 2 of Myosin phosphatase Rho-interacting protein [OS=Homo sapiens]	S993	2.40
Q7L2J0	MEPCE	7SK snRNA methylphosphate capping enzyme [OS=Homo sapiens]	S254	2.40
Q9UKV3	ACIN1	Apoptotic chromatin condensation inducer in the nucleus [OS=Homo sapiens]	S386; S388	2.42
Q9C0C2	TNKS1BP1	182 kDa tankyrase-1-binding protein [OS=Homo sapiens]	S1620; S1621	2.43

Q9UQ35	SRRM2	Serine/arginine repetitive matrix protein 2 [OS=Homo sapiens]	S2426	2.49
Q7KZI7-14	MARK2	Isoform 14 of Serine/threonine-protein kinase MARK2 [OS=Homo sapiens]	S376	2.49
P08238	HSP90AB1	Heat shock protein HSP 90-beta [OS=Homo sapiens]	S255	2.49
Q9UQ35	SRRM2	Serine/arginine repetitive matrix protein 2 [OS=Homo sapiens]	T1208	2.50
Q9BTE3	MCMBP	Mini-chromosome maintenance complex-binding protein [OS=Homo sapiens]	S154	2.53
Q9Y2W1	THRAP3	Thyroid hormone receptor-associated protein 3 [OS=Homo sapiens]	S672	2.54
P32004	L1CAM	Neural cell adhesion molecule L1 [OS=Homo sapiens]	S1243	2.55
Q5T200	ZC3H13	Zinc finger CCCH domain-containing protein 13 [OS=Homo sapiens]	T364	2.59
Q8NDT2	RBM15B	Putative RNA-binding protein 15B [OS=Homo sapiens]	S609	2.59
Q8WWI1	LMO7	LIM domain only protein 7 [OS=Homo sapiens]	S751	2.60
P29966	MARCKS	Myristoylated alanine-rich C-kinase substrate [OS=Homo sapiens]	S170	2.62
P35658	NUP214	Nuclear pore complex protein Nup214 [OS=Homo sapiens]	S940	2.67
O75475	PSIP1	PC4 and SFRS1-interacting protein [OS=Homo sapiens]	S273; S275	2.75
Q9UQ35	SRRM2	Serine/arginine repetitive matrix protein 2 [OS=Homo sapiens]	S2740; T2738	2.79
Q9NTI5	PDS5B	Sister chromatid cohesion protein PDS5 homolog B [OS=Homo sapiens]	T1370	2.81
P08651	NFIC	Nuclear factor 1 C-type [OS=Homo sapiens]	S305	2.92
P08238	HSP90AB1	Heat shock protein HSP 90-beta [OS=Homo sapiens]	S226	2.93
Q9C0C2	TNKS1BP1	182 kDa tankyrase-1-binding protein [OS=Homo sapiens]	S1666	2.96
Q9P2E9	RRBP1	Ribosome-binding protein 1 [OS=Homo sapiens]	S583	3.03
Q7Z5L9	IRF2BP2	Interferon regulatory factor 2-binding protein 2 [OS=Homo sapiens]	S460	3.07
P02545-2	LMNA	Isoform C of Prelamin-A/C [OS=Homo sapiens]	S458	3.09
P02545	LMNA	Prelamin-A/C [OS=Homo sapiens]	S458	3.08
Q15424	SAFB	Scaffold attachment factor B1 [OS=Homo sapiens]	S383; S384	3.36
P27824	CANX	Calnexin [OS=Homo sapiens]	S564	3.49
O75367	MACROH2A1	Core histone macro-H2A.1 [OS=Homo sapiens]	T129	3.70

**NT: SC3KO vs. WT**				
**Accesion**	**Gene**	**Name**	**Phosphosite**	**log2(FC)**
P08238	HSP90AB1	Heat shock protein HSP 90-beta [OS=Homo sapiens]	S226	-3.41
Q7Z5L9	IRF2BP2	Interferon regulatory factor 2-binding protein 2 [OS=Homo sapiens]	S460	-3.38
Q9NTI5	PDS5B	Sister chromatid cohesion protein PDS5 homolog B [OS=Homo sapiens]	T1370	-3.24
P35658	NUP214	Nuclear pore complex protein Nup214 [OS=Homo sapiens]	S940	-3.21
Q96MU7	YTHDC1	YTH domain-containing protein 1 [OS=Homo sapiens]	S308	-3.14
Q14134	TRIM29	Tripartite motif-containing protein 29 [OS=Homo sapiens]	S552	-3.11
Q86WR7	PROSER2	Proline and serine-rich protein 2 [OS=Homo sapiens]	S328	-3.10
Q5VZK9	CARMIL1	F-actin-uncapping protein LRRC16A [OS=Homo sapiens]	S1291	-3.10
Q9Y2D5	PALM2AKAP2	A-kinase anchor protein 2 [OS=Homo sapiens]	S748	-3.08
P08238	HSP90AB1	Heat shock protein HSP 90-beta [OS=Homo sapiens]	S255	-3.05

Q9UQ35	SRRM2	Serine/arginine repetitive matrix protein 2 [OS=Homo sapiens]	T2738; S2740	-3.00
*P54727	RAD23B	UV excision repair protein RAD23 homolog B [OS=Homo sapiens]	S160	-2.97
P60174	TPI1	Triosephosphate isomerase [OS=Homo sapiens]	S21	-2.92
Q9C0C2	TNKS1BP1	182 kDa tankyrase-1-binding protein [OS=Homo sapiens]	S1620; S1621	-2.91
Q7RTP6	MICAL3	[F-actin]-monooxygenase MICAL3 [OS=Homo sapiens]	[Y/T] [1818-1831]	-2.85
Q6WCQ1-2	MPRIP	Isoform 2 of Myosin phosphatase Rho-interacting protein [OS=Homo sapiens]	S993	-2.84
P48634	PRRC2A	Protein PRRC2A [OS=Homo sapiens]	S1219	-2.82
Q99590	SCAF11	Protein SCAF11 [OS=Homo sapiens]	S963	-2.81
Q9P206	KIAA1522	Uncharacterized protein KIAA1522 [OS=Homo sapiens]	S862	-2.78
P32004	L1CAM	Neural cell adhesion molecule L1 [OS=Homo sapiens]	S1243	-2.76
Q03164	KMT2A	Histone-lysine N-methyltransferase 2A [OS=Homo sapiens]	[504-527]	-2.75
Q8WWI1	LMO7	LIM domain only protein 7 [OS=Homo sapiens]	S751	-2.75
Q9Y6N7	ROBO1	Roundabout homolog 1 [OS=Homo sapiens]	S1055	-2.73
Q7Z417	NUFIP2	Nuclear fragile X mental retardation-interacting protein 2 [OS=Homo sapiens]	S629	-2.72
Q9UQ35	SRRM2	Serine/arginine repetitive matrix protein 2 [OS=Homo sapiens]	S2426	-2.69
P12270	TPR	Nucleoprotein TPR [OS=Homo sapiens]	[644-669]	-2.68
O60333	KIF1B	Kinesin-like protein KIF1B [OS=Homo sapiens]	S1057	-2.68
Q5T200	ZC3H13	Zinc finger CCCH domain-containing protein 13 [OS=Homo sapiens]	T364	-2.68
Q9UQ35	SRRM2	Serine/arginine repetitive matrix protein 2 [OS=Homo sapiens]	T1208	-2.68
Q6ZRV2	FAM83H	Protein FAM83H [OS=Homo sapiens]	[508-520]	-2.67
Q02241-2	KIF23	Isoform 2 of Kinesin-like protein KIF23 [OS=Homo sapiens]	S684	-2.67
Q9Y2W1	THRAP3	Thyroid hormone receptor-associated protein 3 [OS=Homo sapiens]	S672	-2.63
O43896	KIF1C	Kinesin-like protein KIF1C [OS=Homo sapiens]	[1078-1088]	-2.6163
P24928	POLR2A	DNA-directed RNA polymerase II subunit RPB1 [OS=Homo sapiens]	S1913; S1920	-2.62
Q13428-3	TCOF1	Isoform 3 of Treacle protein [OS=Homo sapiens]	S233	-2.62
Q13428-6	TCOF1	Isoform 6 of Treacle protein [OS=Homo sapiens]	S233	-2.61
P24928	POLR2A	DNA-directed RNA polymerase II subunit RPB1 [OS=Homo sapiens]	S1927; S1934	-2.61
Q13428-7	TCOF1	Isoform 7 of Treacle protein [OS=Homo sapiens]	S233	-2.60
Q14980	NUMA1	Nuclear mitotic apparatus protein 1 [OS=Homo sapiens]	S1969	-2.60
Q09666	AHNAK	Neuroblast differentiation-associated protein AHNAK [OS=Homo sapiens]	S3426	-2.59
Q9BTE3	MCMBP	Mini-chromosome maintenance complex-binding protein [OS=Homo sapiens]	S154	-2.57
Q8WY36	BBX	HMG box transcription factor BBX [OS=Homo sapiens]	S844	-2.53
P29966	MARCKS	Myristoylated alanine-rich C-kinase substrate [OS=Homo sapiens]	S170	-2.49
*O00515	LAD1	Ladinin-1 [OS=Homo sapiens]	S272	-2.47
Q16181	SEPTIN7	Septin-7 [OS=Homo sapiens]	T426	-2.45
P46013	MKI67	Proliferation marker protein Ki-67 [OS=Homo sapiens]	S2528	-2.45
P38159	RBMX	RNA-binding motif protein, X chromosome [OS=Homo sapiens]	[310-324]	-2.43

Q14814	MEF2D	Myocyte-specific enhancer factor 2D [OS=Homo sapiens]	S231	-2.43
Q0VG06	FAAP100	Fanconi anemia core complex-associated protein 100 [OS=Homo sapiens]	Y870	-2.42
Q9ULJ3	ZBTB21	Zinc finger and BTB domain-containing protein 21 [OS=Homo sapiens]	S411	-2.41
Q5T200	ZC3H13	Zinc finger CCCH domain-containing protein 13 [OS=Homo sapiens]	S77	-2.41
Q9NSK0	KLC4	Kinesin light chain 4 [OS=Homo sapiens]	S611	-2.41
P11388	TOP2A	DNA topoisomerase 2-alpha [OS=Homo sapiens]	[1374-1411]	-2.38
Q01082	SPTBN1	Spectrin beta chain, non-erythrocytic 1 [OS=Homo sapiens]	S2161; S2165	-2.37
Q6IQ22	RAB12	Ras-related protein Rab-12 [OS=Homo sapiens]	S21	-2.35
Q92597	NDRG1	Protein NDRG1 [OS=Homo sapiens]	[344-353]; [354-363]	-2.35
Q7Z6E9	RBBP6	E3 ubiquitin-protein ligase RBBP6 [OS=Homo sapiens]	S1179	-2.35
Q92615	LARP4B	La-related protein 4B [OS=Homo sapiens]	S731; S736	-2.35
Q8NDT2	RBM15B	Putative RNA-binding protein 15B [OS=Homo sapiens]	S609	-2.34
O95239	KIF4A	Chromosome-associated kinesin KIF4A [OS=Homo sapiens]	S801	-2.34
Q92974	ARHGEF2	Rho guanine nucleotide exchange factor 2 [OS=Homo sapiens]	S960	-2.31
*Q8WX93	PALLD	Palladin [OS=Homo sapiens]	S641	-2.30
Q9Y2W1	THRAP3	Thyroid hormone receptor-associated protein 3 [OS=Homo sapiens]	S682	-2.29
Q9ULH1	ASAP1	Arf-GAP with SH3 domain, ANK repeat and PH domain-containing protein 1 [OS=Homo sapiens]	S1027	-2.27
Q9C0B5	ZDHHC5	Palmitoyltransferase ZDHHC5 [OS=Homo sapiens]	S529	-2.27
P02545	LMNA	Prelamin-A/C [OS=Homo sapiens]	S458	-2.27
Q9Y520	PRRC2C	Protein PRRC2C [OS=Homo sapiens]	S1544	-2.25
P02545-2	LMNA	Isoform C of Prelamin-A/C [OS=Homo sapiens]	S458	-2.25
P38159	RBMX	RNA-binding motif protein, X chromosome [OS=Homo sapiens]	[325-339]	-2.24
Q92922	SMARCC1	SWI/SNF complex subunit SMARCC1 [OS=Homo sapiens]	S328; S330	-2.24
Q9Y6M7-7	SLC4A7	Isoform 7 of Sodium bicarbonate cotransporter 3 [OS=Homo sapiens]	S1258	-2.24
Q9NTI5	PDS5B	Sister chromatid cohesion protein PDS5 homolog B [OS=Homo sapiens]	S1358	-2.19
O75376	NCOR1	Nuclear receptor corepressor 1 [OS=Homo sapiens]	S1472	-2.19
P55196	AFDN	Afadin [OS=Homo sapiens]	S1721	-2.19
P23396	RPS3	40S ribosomal protein S3 [OS=Homo sapiens]	[215-243]	-2.17
Q96E09	PABIR1	PPP2R1A-PPP2R2A-interacting phosphatase regulator 1 [OS=Homo sapiens]	S37	-2.16
Q8IY67-2	RAVER1	Isoform 2 of Ribonucleoprotein PTB-binding 1 [OS=Homo sapiens]	S567	-2.16
Q9NZN8	CNOT2	CCR4-NOT transcription complex subunit 2 [OS=Homo sapiens]	S165	-2.13
Q15149-4	PLEC	Isoform 4 of Plectin [OS=Homo sapiens]	S1584	-2.13
Q7Z5L9	IRF2BP2	Interferon regulatory factor 2-binding protein 2 [OS=Homo sapiens]	S360	-2.12
Q9UK58	CCNL1	Cyclin-L1 [OS=Homo sapiens]	S352	-2.11
A0MZ66	SHTN1	Shootin-1 [OS=Homo sapiens]	S534	-2.09
O60841	EIF5B	Eukaryotic translation initiation factor 5B [OS=Homo sapiens]	S214	-2.09
Q9NYF8	BCLAF1	Bcl-2-associated transcription factor 1 [OS=Homo sapiens]	S496	-2.09

O75179	ANKRD17	Ankyrin repeat domain-containing protein 17 [OS=Homo sapiens]	S2041	-2.07
P38159	RBMX	RNA-binding motif protein, X chromosome [OS=Homo sapiens]	[245-258]	-2.07
Q86YV5	PRAG1	Inactive tyrosine-protein kinase PRAG1 [OS=Homo sapiens]	S745	-2.06
P17096-2	HMGA1	Isoform HMG-Y of High mobility group protein HMG-I/HMG-Y [OS=Homo sapiens]	T42	-2.04
Q8N556	AFAP1	Actin filament-associated protein 1 [OS=Homo sapiens]	[246-275]	-2.04
Q9C0C2	TNKS1BP1	182 kDa tankyrase-1-binding protein [OS=Homo sapiens]	S1652	-2.04
Q7Z309-3	PABIR2	Isoform 3 of PABIR family member 2 [OS=Homo sapiens]	S63	-2.03
Q13439	GOLGA4	Golgin subfamily A member 4 [OS=Homo sapiens]	S71	-2.02
Q9H1E3	NUCKS1	Nuclear ubiquitous casein and cyclin-dependent kinase substrate 1 [OS=Homo sapiens]	S54	-2.01
P49790	NUP153	Nuclear pore complex protein Nup153 [OS=Homo sapiens]	S209	-2.00
O43237	DYNC1LI2	Cytoplasmic dynein 1 light intermediate chain 2 [OS=Homo sapiens]	S391	-2.00
Q86X27	RALGPS2	Ras-specific guanine nucleotide-releasing factor RalGPS2 [OS=Homo sapiens]	S296	-2.00
Q5T0W9	FAM83B	Protein FAM83B [OS=Homo sapiens]	S388	-1.99
P27824	CANX	Calnexin [OS=Homo sapiens]	S554	-1.98
Q14839	CHD4	Chromodomain-helicase-DNA-binding protein 4 [OS=Homo sapiens]	S1535	-1.98
O15234	CASC3	Protein CASC3 [OS=Homo sapiens]	S148	-1.97
P36507	MAP2K2	Dual specificity mitogen-activated protein kinase kinase 2 [OS=Homo sapiens]	T394	-1.94
Q9UKV3	ACIN1	Apoptotic chromatin condensation inducer in the nucleus [OS=Homo sapiens]	S838	-1.93
Q14247	CTTN	Src substrate cortactin [OS=Homo sapiens]	T401; T411	-1.93
*Q92598	HSPH1	Heat shock protein 105 kDa [OS=Homo sapiens]	S809	-1.93
*Q7Z6Z7	HUWE1	E3 ubiquitin-protein ligase HUWE1 [OS=Homo sapiens]	T2889	-1.92
P09651	HNRNPA1	Heterogeneous nuclear ribonucleoprotein A1 [OS=Homo sapiens]	[353-370]	-1.92
Q9BQG0	MYBBP1A	Myb-binding protein 1A [OS=Homo sapiens]	[1152-1167]	-1.91
Q9Y5J1	UTP18	U3 small nucleolar RNA-associated protein 18 homolog [OS=Homo sapiens]	S206; S210	-1.88
P16070	CD44	CD44 antigen [OS=Homo sapiens]	S697	-1.87
O60841	EIF5B	Eukaryotic translation initiation factor 5B [OS=Homo sapiens]	S164	-1.86
*Q52LW3	ARHGAP29	Rho GTPase-activating protein 29 [OS=Homo sapiens]	S1019	-1.85
Q96JP5	ZFP91	E3 ubiquitin-protein ligase ZFP91 [OS=Homo sapiens]	S103	-1.83
Q13586	STIM1	Stromal interaction molecule 1 [OS=Homo sapiens]	S660	-1.83
Q86VM9	ZC3H18	Zinc finger CCCH domain-containing protein 18 [OS=Homo sapiens]	S534	-1.82
P48634	PRRC2A	Protein PRRC2A [OS=Homo sapiens]	S761	-1.80
Q13428-6	TCOF1	Isoform 6 of Treacle protein [OS=Homo sapiens]	S381	-1.80
Q99575	POP1	Ribonucleases P/MRP protein subunit POP1 [OS=Homo sapiens]	[719-740]	-1.78
Q9NRA8	EIF4ENIF1	Eukaryotic translation initiation factor 4E transporter [OS=Homo sapiens]	S577	-1.78
P52594	AGFG1	Arf-GAP domain and FG repeat-containing protein 1 [OS=Homo sapiens]	S181	-1.77

Q8WWM7	ATXN2L	Ataxin-2-like protein [OS=Homo sapiens]	S684	-1.75
P53396	ACLY	ATP-citrate synthase [OS=Homo sapiens]	S455	-1.75
P25788	PSMA3	Proteasome subunit alpha type-3 [OS=Homo sapiens]	S250	-1.73
*P06702	S100A9	Protein S100-A9 [OS=Homo sapiens]	T113	-1.73
Q7Z6Z7	HUWE1	E3 ubiquitin-protein ligase HUWE1 [OS=Homo sapiens]	[3552-3570]	-1.72
Q5T200	ZC3H13	Zinc finger CCCH domain-containing protein 13 [OS=Homo sapiens]	S64	-1.69
Q8WXF7	ATL1	Atlastin-1 [OS=Homo sapiens]	S10	-1.68
*Q9UQN3	CHMP2B	Charged multivesicular body protein 2b [OS=Homo sapiens]	S199	-1.68
*Q9NZT2	OGFR	Opioid growth factor receptor [OS=Homo sapiens]	S378	-1.65
Q9Y618	NCOR2	Nuclear receptor corepressor 2 [OS=Homo sapiens]	S956	-1.65
O15427	SLC16A3	Monocarboxylate transporter 4 [OS=Homo sapiens]	T463; S464	-1.64
Q15648	MED1	Mediator of RNA polymerase II transcription subunit 1 [OS=Homo sapiens]	S1156	-1.64
Q3KQU3	MAP7D1	MAP7 domain-containing protein 1 [OS=Homo sapiens]	S116	-1.60
P04049	RAF1	RAF proto-oncogene serine/threonine-protein kinase [OS=Homo sapiens]	S43	-1.59
Q01082	SPTBN1	Spectrin beta chain, non-erythrocytic 1 [OS=Homo sapiens]	S2169	-1.59
Q86SQ0	PHLDB2	Pleckstrin homology-like domain family B member 2 [OS=Homo sapiens]	T898	-1.58
Q9UQE7	SMC3	Structural maintenance of chromosomes protein 3 [OS=Homo sapiens]	S1067	-1.57
*Q99618	CDCA3	Cell division cycle-associated protein 3 [OS=Homo sapiens]	S68	-1.56
Q9NYB9	ABI2	Abl interactor 2 [OS=Homo sapiens]	S183	-1.56
P54259	ATN1	Atrophin-1 [OS=Homo sapiens]	S645	-1.56
*Q9NZT2	OGFR	Opioid growth factor receptor [OS=Homo sapiens]	S315	-1.56
Q9C0B5	ZDHHC5	Palmitoyltransferase ZDHHC5 [OS=Homo sapiens]	[583-597]	-1.55
Q14247	CTTN	Src substrate cortactin [OS=Homo sapiens]	T401	-1.55
Q4G0J3	LARP7	La-related protein 7 [OS=Homo sapiens]	S261	-1.55
Q86VM9	ZC3H18	Zinc finger CCCH domain-containing protein 18 [OS=Homo sapiens]	S532	-1.54
Q9UQ35	SRRM2	Serine/arginine repetitive matrix protein 2 [OS=Homo sapiens]	S1132	-1.54
Q8IU81	IRF2BP1	Interferon regulatory factor 2-binding protein 1 [OS=Homo sapiens]	S384	-1.53
Q13428-3	TCOF1	Isoform 3 of Treacle protein [OS=Homo sapiens]	S381	-1.53
Q13428-7	TCOF1	Isoform 7 of Treacle protein [OS=Homo sapiens]	S381	-1.52
Q9BQG0	MYBBP1A	Myb-binding protein 1A [OS=Homo sapiens]	S1267	-1.52
*Q9Y3Q8	TSC22D4	TSC22 domain family protein 4 [OS=Homo sapiens]	T229	-1.52
Q3KQU3	MAP7D1	MAP7 domain-containing protein 1 [OS=Homo sapiens]	T813	-1.52
Q9Y2W1	THRAP3	Thyroid hormone receptor-associated protein 3 [OS=Homo sapiens]	S55	-1.52
Q13442	PDAP1	28 kDa heat- and acid-stable phosphoprotein [OS=Homo sapiens]	S60; S63	-1.51
Q05682-4	CALD1	Isoform 4 of Caldesmon [OS=Homo sapiens]	S202	-1.50
*O00515	LAD1	Ladinin-1 [OS=Homo sapiens]	S394	-1.50
Q9H2G2	SLK	STE20-like serine/threonine-protein kinase [OS=Homo sapiens]	S779	-1.50
*Q9H501	ESF1	ESF1 homolog [OS=Homo sapiens]	S694	-1.50

*Q96CV9	OPTN	Optineurin [OS=Homo sapiens]	S526	-1.50
Q6P2E9	EDC4	Enhancer of mRNA-decapping protein 4 [OS=Homo sapiens]	[727-754]	-1.49
*Q5UIP0	RIF1	Telomere-associated protein RIF1 [OS=Homo sapiens]	S1542	-1.48
*Q9NZ63	C9orf78	Telomere length and silencing protein 1 homolog [OS=Homo sapiens]	S261	-1.47
*Q13501	SQSTM1	Sequestosome-1 [OS=Homo sapiens]	S272	-1.47
*Q8N556	AFAP1	Actin filament-associated protein 1 [OS=Homo sapiens]	S548	-1.47
*O95425-2	SVIL	Isoform 2 of Supervillin [OS=Homo sapiens]	S221	-1.46
Q96D71	REPS1	RalBP1-associated Eps domain-containing protein 1 [OS=Homo sapiens]	S709	-1.46
Q7Z4S6	KIF21A	Kinesin-like protein KIF21A [OS=Homo sapiens]	S1212	-1.46
Q92609	TBC1D5	TBC1 domain family member 5 [OS=Homo sapiens]	S554	-1.46
Q04637	EIF4G1	Eukaryotic translation initiation factor 4 gamma 1 [OS=Homo sapiens]	T205	-1.46
Q8ND76	CCNY	Cyclin-Y [OS=Homo sapiens]	S326	1.85
P49790	NUP153	Nuclear pore complex protein Nup153 [OS=Homo sapiens]	S334	1.99
Q5T200	ZC3H13	Zinc finger CCCH domain-containing protein 13 [OS=Homo sapiens]	S325	2.05
Q7Z309-3	PABIR2	Isoform 3 of PABIR family member 2 [OS=Homo sapiens]	S25	2.11
Q96E09	PABIR1	PPP2R1A-PPP2R2A-interacting phosphatase regulator 1 [OS=Homo sapiens]	S76	2.14
Q9NSK0	KLC4	Kinesin light chain 4 [OS=Homo sapiens]	S590	2.17
Q92597	NDRG1	Protein NDRG1 [OS=Homo sapiens]	S330; S333; S336	2.18
Q96JY6	PDLIM2	PDZ and LIM domain protein 2 [OS=Homo sapiens]	S123; S129	2.24
Q9Y6N7	ROBO1	Roundabout homolog 1 [OS=Homo sapiens]	S940	2.24
Q13439	GOLGA4	Golgin subfamily A member 4 [OS=Homo sapiens]	S41	2.32
Q8TDM6	DLG5	Disks large homolog 5 [OS=Homo sapiens]	T1011	2.47
P49585	PCYT1A	Choline-phosphate cytidylyltransferase A [OS=Homo sapiens]	S362	2.48
Q86YV5	PRAG1	Inactive tyrosine-protein kinase PRAG1 [OS=Homo sapiens]	[138-154]	2.52
P67870	CSNK2B	Casein kinase II subunit beta [OS=Homo sapiens]	S209	3.06

**MK: SC3KO vs. NT**				
**Accesion**	**Gene**	**Name**	**Phosphosite**	**log2(FC)**
Q13029	PRDM2	PR domain zinc finger protein 2 [OS=Homo sapiens]	S643	-6.71
Q7L7X3	TAOK1	Serine/threonine-protein kinase TAO1 [OS=Homo sapiens]	S974	-6.69
Q13541	EIF4EBP1	Eukaryotic translation initiation factor 4E-binding protein 1 [OS=Homo sapiens]	S65	-6.64
Q8WWI1	LMO7	LIM domain only protein 7 [OS=Homo sapiens]	S116	-6.63
Q8IYB3	SRRM1	Serine/arginine repetitive matrix protein 1 [OS=Homo sapiens]	S738; S740	-6.60
Q9UJU6	DBNL	Drebrin-like protein [OS=Homo sapiens]	S269	-6.58
Q14160	SCRIB	Protein scribble homolog [OS=Homo sapiens]	S1378	-6.56
Q04637	EIF4G1	Eukaryotic translation initiation factor 4 gamma 1 [OS=Homo sapiens]	S1209	-6.55
Q9BST9	RTKN	Rhotekin [OS=Homo sapiens]	S106	-6.53
Q9ULM3	YEATS2	YEATS domain-containing protein 2 [OS=Homo sapiens]	[524-540]	-6.49
Q9NX40	OCIAD1	OCIA domain-containing protein 1 [OS=Homo sapiens]	S108	-6.45
Q6PKG0	LARP1	La-related protein 1 [OS=Homo sapiens]	[1054-1083]	-6.40

Q09666	AHNAK	Neuroblast differentiation-associated protein AHNAK [OS=Homo sapiens]	S93	-6.40
Q6WKZ4	RAB11FIP1	Rab11 family-interacting protein 1 [OS=Homo sapiens]	[433-443]	-6.26
Q9H1E3	NUCKS1	Nuclear ubiquitous casein and cyclin-dependent kinase substrate 1 [OS=Homo sapiens]	S19	-5.76
Q8TB72	PUM2	Pumilio homolog 2 [OS=Homo sapiens]	S589	-5.70
O15231-3	ZNF185	Isoform 3 of Zinc finger protein 185 [OS=Homo sapiens]	S206	-4.40
*Q8WVC0	LEO1	RNA polymerase-associated protein LEO1 [OS=Homo sapiens]	S658	-4.31
P48634	PRRC2A	Protein PRRC2A [OS=Homo sapiens]	S1219	-4.29
Q9UQN3	CHMP2B	Charged multivesicular body protein 2b [OS=Homo sapiens]	S199	-4.28
Q96F86	EDC3	Enhancer of mRNA-decapping protein 3 [OS=Homo sapiens]	S161	-4.17
Q9NWH9	SLTM	SAFB-like transcription modulator [OS=Homo sapiens]	S553	-4.14
Q5T0W9	FAM83B	Protein FAM83B [OS=Homo sapiens]	S764	-4.11
*P04792	HSPB1	Heat shock protein beta-1 [OS=Homo sapiens]	S15	-4.10
O60231	DHX16	Pre-mRNA-splicing factor ATP-dependent RNA helicase DHX16 [OS=Homo sapiens]	S103; S106	-4.01
O75367	MACROH2A1	Core histone macro-H2A.1 [OS=Homo sapiens]	T129 [122-135]	-3.93
Q13428-3	TCOF1	Isoform 3 of Treacle protein [OS=Homo sapiens]	S233	-3.89
Q13428-6	TCOF1	Isoform 6 of Treacle protein [OS=Homo sapiens]	S233	-3.88
Q13428-7	TCOF1	Isoform 7 of Treacle protein [OS=Homo sapiens]	S233	-3.87
Q99590	SCAF11	Protein SCAF11 [OS=Homo sapiens]	S796	-3.87
O15231-3	ZNF185	Isoform 3 of Zinc finger protein 185 [OS=Homo sapiens]	S520; T506	-3.80
Q96T58	SPEN	Msx2-interacting protein [OS=Homo sapiens]	S1287	-3.79
Q5VT52	RPRD2	Regulation of nuclear pre-mRNA domain-containing protein 2 [OS=Homo sapiens]	T723	-3.71
Q7Z5L9	IRF2BP2	Interferon regulatory factor 2-binding protein 2 [OS=Homo sapiens]	S460	-3.65
*Q9C0C2	TNKS1BP1	182 kDa tankyrase-1-binding protein [OS=Homo sapiens]	S1620; S1621	-3.64
Q15424	SAFB	Scaffold attachment factor B1 [OS=Homo sapiens]	S383; S384	-3.60
Q07955	SRSF1	Serine/arginine-rich splicing factor 1 [OS=Homo sapiens]	S238	-3.59
Q14134	TRIM29	Tripartite motif-containing protein 29 [OS=Homo sapiens]	S552	-3.57
*O00515	LAD1	Ladinin-1 [OS=Homo sapiens]	S38	-3.47
Q7RTP6	MICAL3	[F-actin]-monooxygenase MICAL3 [OS=Homo sapiens]	[Y/T] [1818-1835]	-3.47
P23588	EIF4B	Eukaryotic translation initiation factor 4B [OS=Homo sapiens]	S597	-3.46
P27824	CANX	Calnexin [OS=Homo sapiens]	S564	-3.42
P32004	L1CAM	Neural cell adhesion molecule L1 [OS=Homo sapiens]	S1243	-3.36
Q14155-1	ARHGEF7	Isoform 1 of Rho guanine nucleotide exchange factor 7 [OS=Homo sapiens]	[514-537]	-3.34
O95239	KIF4A	Chromosome-associated kinesin KIF4A [OS=Homo sapiens]	S801	-3.33
Q96CV9	OPTN	Optineurin [OS=Homo sapiens]	[525-537]	-3.32
Q9Y2W1	THRAP3	Thyroid hormone receptor-associated protein 3 [OS=Homo sapiens]	S672	-3.30
Q5T200	ZC3H13	Zinc finger CCCH domain-containing protein 13 [OS=Homo sapiens]	T364	-3.25
Q8WY36	BBX	HMG box transcription factor BBX [OS=Homo sapiens]	S844	-3.25

Q9ULJ3	ZBTB21	Zinc finger and BTB domain-containing protein 21 [OS=Homo sapiens]	S411; S422	-3.24
Q6WCQ1-2	MPRIP	Isoform 2 of Myosin phosphatase Rho-interacting protein [OS=Homo sapiens]	S993	-3.24
Q9UQ35	SRRM2	Serine/arginine repetitive matrix protein 2 [OS=Homo sapiens]	T1208	-3.24
Q14980	NUMA1	Nuclear mitotic apparatus protein 1 [OS=Homo sapiens]	S1969	-3.23
*P06702	S100A9	Protein S100-A9 [OS=Homo sapiens]	T113	-3.20
Q86UP2	KTN1	Kinectin [OS=Homo sapiens]	T153	-3.19
P46013	MKI67	Proliferation marker protein Ki-67 [OS=Homo sapiens]	S1071	-3.18
Q6UN15	FIP1L1	Pre-mRNA 3'-end-processing factor FIP1 [OS=Homo sapiens]	S492	-3.18
Q7L2J0	MEPCE	7SK snRNA methylphosphate capping enzyme [OS=Homo sapiens]	S254	-3.17
Q00587	CDC42EP1	Cdc42 effector protein 1 [OS=Homo sapiens]	S192	-3.17
Q86WR7	PROSER2	Proline and serine-rich protein 2 [OS=Homo sapiens]	S328	-3.17
Q12789	GTF3C1	General transcription factor 3C polypeptide 1 [OS=Homo sapiens]	[508-521]	-3.15
P29966	MARCKS	Myristoylated alanine-rich C-kinase substrate [OS=Homo sapiens]	S170	-3.13
Q9Y6N7	ROBO1	Roundabout homolog 1 [OS=Homo sapiens]	S1055	-3.12
Q9Y580	RBM7	RNA-binding protein 7 [OS=Homo sapiens]	S204	-3.12
Q8IXM2	BAP18	Chromatin complexes subunit BAP18 [OS=Homo sapiens]	S96	-3.11
Q16204	CCDC6	Coiled-coil domain-containing protein 6 [OS=Homo sapiens]	S244	-3.09
Q8NDT2	RBM15B	Putative RNA-binding protein 15B [OS=Homo sapiens]	S609	-3.05
P46013	MKI67	Proliferation marker protein Ki-67 [OS=Homo sapiens]	[1218-1245]	-3.04
Q5T200	ZC3H13	Zinc finger CCCH domain-containing protein 13 [OS=Homo sapiens]	S77	-3.01
O75475	PSIP1	PC4 and SFRS1-interacting protein [OS=Homo sapiens]	S273; S275	-3.01
Q02241-2	KIF23	Isoform 2 of Kinesin-like protein KIF23 [OS=Homo sapiens]	S684	-3.00
P40818	USP8	Ubiquitin carboxyl-terminal hydrolase 8 [OS=Homo sapiens]	S718	-3.00
Q92974	ARHGEF2	Rho guanine nucleotide exchange factor 2 [OS=Homo sapiens]	S960	-2.99
*Q13442	PDAP1	28 kDa heat- and acid-stable phosphoprotein [OS=Homo sapiens]	S60	-2.98
Q9P107	GMIP	GEM-interacting protein [OS=Homo sapiens]	S437	-2.98
Q8IUD2	ERC1	ELKS/Rab6-interacting/CAST family member 1 [OS=Homo sapiens]	S191	-2.97
A0MZ66	SHTN1	Shootin-1 [OS=Homo sapiens]	S534	-2.96
P29350	PTPN6	Tyrosine-protein phosphatase non-receptor type 6 [OS=Homo sapiens]	[555-570]	-2.95
Q6ZRV2	FAM83H	Protein FAM83H [OS=Homo sapiens]	S998	-2.95
Q7Z417	NUFIP2	Nuclear fragile X mental retardation-interacting protein 2 [OS=Homo sapiens]	S629	-2.95
Q9UQ35	SRRM2	Serine/arginine repetitive matrix protein 2 [OS=Homo sapiens]	S2426	-2.92
Q8WWM7	ATXN2L	Ataxin-2-like protein [OS=Homo sapiens]	S684	-2.90
*Q13442	PDAP1	28 kDa heat- and acid-stable phosphoprotein [OS=Homo sapiens]	S60; S63	-2.89
P38159	RBMX	RNA-binding motif protein, X chromosome [OS=Homo sapiens]	S352	-2.89
Q9BXP5	SRRT	Serrate RNA effector molecule homolog [OS=Homo sapiens]	T544	-2.87

Q9H1E3	NUCKS1	Nuclear ubiquitous casein and cyclin-dependent kinase substrate 1 [OS=Homo sapiens]	S54	-2.87
Q6ZRS2	SRCAP	Helicase SRCAP [OS=Homo sapiens]	S1859	-2.86
Q03164	KMT2A	Histone-lysine N-methyltransferase 2A [OS=Homo sapiens]	[504-527]	-2.85
Q9Y2W1	THRAP3	Thyroid hormone receptor-associated protein 3 [OS=Homo sapiens]	S682	-2.85
Q7Z417	NUFIP2	Nuclear fragile X mental retardation-interacting protein 2 [OS=Homo sapiens]	S112	-2.83
Q8IYB3	SRRM1	Serine/arginine repetitive matrix protein 1 [OS=Homo sapiens]	S260	-2.83
Q9P206	KIAA1522	Uncharacterized protein KIAA1522 [OS=Homo sapiens]	S862	-2.83
Q6ZRV2	FAM83H	Protein FAM83H [OS=Homo sapiens]	[508-520]	-2.81
Q9NSK0	KLC4	Kinesin light chain 4 [OS=Homo sapiens]	S611	-2.80
P12270	TPR	Nucleoprotein TPR [OS=Homo sapiens]	[644-669]	-2.79
P46013	MKI67	Proliferation marker protein Ki-67 [OS=Homo sapiens]	S357	-2.78
Q9Y6M7-7	SLC4A7	Isoform 7 of Sodium bicarbonate cotransporter 3 [OS=Homo sapiens]	S1258	-2.76
Q6IQ22	RAB12	Ras-related protein Rab-12 [OS=Homo sapiens]	S21 [10-30]	-2.75
*P29590-2	PML	Isoform PML-5 of Protein PML [OS=Homo sapiens]	S518/S527	-2.74
Q9UQ35	SRRM2	Serine/arginine repetitive matrix protein 2 [OS=Homo sapiens]	S1600; S1601	-2.72
P24928	POLR2A	DNA-directed RNA polymerase II subunit RPB1 [OS=Homo sapiens]	S1913; S1920; S1927; S1934	-2.71
Q9UKV3	ACIN1	Apoptotic chromatin condensation inducer in the nucleus [OS=Homo sapiens]	S386, S388	-2.71
Q8NEY8	PPHLN1	Periphilin-1 [OS=Homo sapiens]	S133	-2.70
P29966	MARCKS	Myristoylated alanine-rich C-kinase substrate [OS=Homo sapiens]	T150	-2.68
*Q14847	LASP1	LIM and SH3 domain protein 1 [OS=Homo sapiens]	T104	-2.68
P08651	NFIC	Nuclear factor 1 C-type [OS=Homo sapiens]	S323	-2.66
P16144	ITGB4	Integrin beta-4 [OS=Homo sapiens]	T1530	-2.66
Q9Y520	PRRC2C	Protein PRRC2C [OS=Homo sapiens]	T2673	-2.66
Q14C86	GAPVD1	GTPase-activating protein and VPS9 domain-containing protein 1 [OS=Homo sapiens]	S914	-2.66
Q9UQ35	SRRM2	Serine/arginine repetitive matrix protein 2 [OS=Homo sapiens]	[304-329]	-2.66
Q86UU0	BCL9L	B-cell CLL/lymphoma 9-like protein [OS=Homo sapiens]	S21	-2.64
*Q9NTI5	PDS5B	Sister chromatid cohesion protein PDS5 homolog B [OS=Homo sapiens]	T1370	-2.64
Q96E09	PABIR1	PPP2R1A-PPP2R2A-interacting phosphatase regulator 1 [OS=Homo sapiens]	S37	-2.62
Q04726	TLE3	Transducin-like enhancer protein 3 [OS=Homo sapiens]	[278-300]	-2.60
P02545-2	LMNA	Isoform C of Prelamin-A/C [OS=Homo sapiens]	S277	-2.58
P02545	LMNA	Prelamin-A/C [OS=Homo sapiens]	S277	-2.57
Q9NR30	DDX21	Nucleolar RNA helicase 2 [OS=Homo sapiens]	S121	-2.57
*P10644	PRKAR1A	cAMP-dependent protein kinase type I-alpha regulatory subunit [OS=Homo sapiens]	S77/S83	-2.56
P11388	TOP2A	DNA topoisomerase 2-alpha [OS=Homo sapiens]	[1374-1411]	-2.55
Q9NYF8	BCLAF1	Bcl-2-associated transcription factor 1 [OS=Homo sapiens]	S531	-2.55
Q92615	LARP4B	La-related protein 4B [OS=Homo sapiens]	S524	-2.55
Q9UQ35	SRRM2	Serine/arginine repetitive matrix protein 2 [OS=Homo sapiens]	S1179	-2.54

Q86YV5	PRAG1	Inactive tyrosine-protein kinase PRAG1 [OS=Homo sapiens]	S745	-2.53
Q01082	SPTBN1	Spectrin beta chain, non-erythrocytic 1 [OS=Homo sapiens]	S2161/S2165	-2.53
Q9NYF8-4	BCLAF1	Isoform 4 of Bcl-2-associated transcription factor 1 [OS=Homo sapiens]	S358	-2.52
P46937	YAP1	Transcriptional coactivator YAP1 [OS=Homo sapiens]	S105/S109	-2.51
*P32519	ELF1	ETS-related transcription factor Elf-1 [OS=Homo sapiens]	S187	-2.50
Q14247	CTTN	Src substrate cortactin [OS=Homo sapiens]	T411	-2.49
Q8N8A6	DDX51	ATP-dependent RNA helicase DDX51 [OS=Homo sapiens]	S83	-2.47
Q7Z417	NUFIP2	Nuclear fragile X mental retardation-interacting protein 2 [OS=Homo sapiens]	[564-581]	-2.45
Q96A49	SYAP1	Synapse-associated protein 1 [OS=Homo sapiens]	T248	-2.45
*Q9Y3Q8	TSC22D4	TSC22 domain family protein 4 [OS=Homo sapiens]	S165	-2.45
*O43399	TPD52L2	Tumor protein D54 [OS=Homo sapiens]	S166	-2.44
*Q15629	TRAM1	Translocating chain-associated membrane protein 1 [OS=Homo sapiens]	S365 [348-367]	-2.43
P38159	RBMX	RNA-binding motif protein, X chromosome [OS=Homo sapiens]	[310-324]	-2.42
P42696	RBM34	RNA-binding protein 34 [OS=Homo sapiens]	S28	-2.41
Q96N67	DOCK7	Dedicator of cytokinesis protein 7 [OS=Homo sapiens]	S432	-2.40
*Q9NZT2	OGFR	Opioid growth factor receptor [OS=Homo sapiens]	S378	-2.40
P06400	RB1	Retinoblastoma-associated protein [OS=Homo sapiens]	T356	-2.40
Q9UQ35	SRRM2	Serine/arginine repetitive matrix protein 2 [OS=Homo sapiens]	S994/T983	-2.40
O15234	CASC3	Protein CASC3 [OS=Homo sapiens]	S148	-2.39
*P04792	HSPB1	Heat shock protein beta-1 [OS=Homo sapiens]	S78	-2.39
Q8NFJ5	GPRC5A	Retinoic acid-induced protein 3 [OS=Homo sapiens]	S345	-2.39
Q9H6Z4	RANBP3	Ran-binding protein 3 [OS=Homo sapiens]	S100; S101	-2.38
O43166	SIPA1L1	Signal-induced proliferation-associated 1-like protein 1 [OS=Homo sapiens]	S1433	-2.38
P49454	CENPF	Centromere protein F [OS=Homo sapiens]	[2988-3003]	-2.37
P27816	MAP4	Microtubule-associated protein 4 [OS=Homo sapiens]	S358; S384	-2.35
Q9UKV3	ACIN1	Apoptotic chromatin condensation inducer in the nucleus [OS=Homo sapiens]	S216 [206-22]	-2.34
Q09666	AHNAK	Neuroblast differentiation-associated protein AHNAK [OS=Homo sapiens]	S5731	-2.33
Q9BW71	HIRIP3	HIRA-interacting protein 3 [OS=Homo sapiens]	S227	-2.33
*Q13442	PDAP1	28 kDa heat- and acid-stable phosphoprotein [OS=Homo sapiens]	S63	-2.31
P49023	PXN	Paxillin [OS=Homo sapiens]	S321	-2.30
Q14155-1	ARHGEF7	Isoform 1 of Rho guanine nucleotide exchange factor 7 [OS=Homo sapiens]	S340	-2.29
O75179	ANKRD17	Ankyrin repeat domain-containing protein 17 [OS=Homo sapiens]	S19	-2.29
Q8IY67-2	RAVER1	Isoform 2 of Ribonucleoprotein PTB-binding 1 [OS=Homo sapiens]	S567	-2.28
Q92609	TBC1D5	TBC1 domain family member 5 [OS=Homo sapiens]	S554	-2.28
P19338	NCL	Nucleolin [OS=Homo sapiens]	S67 [64-79]	-2.28
Q07666	KHDRBS1	KH domain-containing, RNA-binding, signal transduction-associated protein 1 [OS=Homo sapiens]	S58	-2.27
O60841	EIF5B	Eukaryotic translation initiation factor 5B [OS=Homo sapiens]	S164	-2.27

Q92922	SMARCC1	SWI/SNF complex subunit SMARCC1 [OS=Homo sapiens]	S328; S330; T335	-2.26
Q9H0D6	XRN2	5'-3' exoribonuclease 2 [OS=Homo sapiens]	S448	-2.26
Q01082	SPTBN1	Spectrin beta chain, non-erythrocytic 1 [OS=Homo sapiens]	S2161; S2169	-2.25
*P35579	MYH9	Myosin-9 [OS=Homo sapiens]	S1943 [1938-1960]	-2.25
P49006	MARCKSL1	MARCKS-related protein [OS=Homo sapiens]	[158-195]	-2.25
P36507	MAP2K2	Dual specificity mitogen-activated protein kinase kinase 2 [OS=Homo sapiens]	T394	-2.24
*Q86X29	LSR	Lipolysis-stimulated lipoprotein receptor [OS=Homo sapiens]	T514	-2.22
Q5T200	ZC3H13	Zinc finger CCCH domain-containing protein 13 [OS=Homo sapiens]	S64	-2.22
*Q9UDY2	TJP2	Tight junction protein ZO-2 [OS=Homo sapiens]	S986 [976-999]	-2.20
*Q9NTI5	PDS5B	Sister chromatid cohesion protein PDS5 homolog B [OS=Homo sapiens]	S1358	-2.20
Q09666	AHNAK	Neuroblast differentiation-associated protein AHNAK [OS=Homo sapiens]	S4986	-2.19
*Q04726	TLE3	Transducin-like enhancer protein 3 [OS=Homo sapiens]	S286	-2.19
O60832	DKC1	H/ACA ribonucleoprotein complex subunit DKC1 [OS=Homo sapiens]	S494	-2.18
P46060	RANGAP1	Ran GTPase-activating protein 1 [OS=Homo sapiens]	S442	-2.16
P16949	STMN1	Stathmin [OS=Homo sapiens]	S38	-2.16
Q9NRA8	EIF4ENIF1	Eukaryotic translation initiation factor 4E transporter [OS=Homo sapiens]	S577	-2.15
O75683	SURF6	Surfeit locus protein 6 [OS=Homo sapiens]	S138	-2.14
*Q92598	HSPH1	Heat shock protein 105 kDa [OS=Homo sapiens]	S809	-2.14
*Q8IZP0	ABI1	Abl interactor 1 [OS=Homo sapiens]	S225	-2.13
Q69YQ0	JPT2	Cytospin-A [OS=Homo sapiens]	[830-842]	-2.13
Q9H910	SPECC1L	Jupiter microtubule associated homolog 2 [OS=Homo sapiens]	S144	-2.13
P27708	CAD	CAD protein [OS=Homo sapiens]	S1859	-2.12
Q9UQ35	SRRM2	Serine/arginine repetitive matrix protein 2 [OS=Homo sapiens]	S2740; T2738	-2.12
P53396	ACLY	ATP-citrate synthase [OS=Homo sapiens]	S455	-2.11
*Q15629	TRAM1	Translocating chain-associated membrane protein 1 [OS=Homo sapiens]	S365 [345-367]	-2.11
*Q9NTI5	PDS5B	Sister chromatid cohesion protein PDS5 homolog B [OS=Homo sapiens]	S1162; S1166; T1381	-2.11
Q8IYB3	SRRM1	Serine/arginine repetitive matrix protein 1 [OS=Homo sapiens]	S769	-2.10
Q9Y2D5	PALM2AKAP2	A-kinase anchor protein 2 [OS=Homo sapiens]	S393	-2.10
Q7Z6Z7	HUWE1	E3 ubiquitin-protein ligase HUWE1 [OS=Homo sapiens]	[3750-3766]	-2.09
O60841	EIF5B	Eukaryotic translation initiation factor 5B [OS=Homo sapiens]	S214	-2.09
Q5SW79	CEP170	Centrosomal protein of 170 kDa [OS=Homo sapiens]	S1160; S1165	-2.09
P38159	RBMX	RNA-binding motif protein, X chromosome [OS=Homo sapiens]	[245-258]	-2.09
*Q9C0C2	TNKS1BP1	182 kDa tankyrase-1-binding protein [OS=Homo sapiens]	S178	-2.09
Q92576	PHF3	PHD finger protein 3 [OS=Homo sapiens]	S283	-2.08
Q7Z4S6	KIF21A	Kinesin-like protein KIF21A [OS=Homo sapiens]	S1212	-2.08
Q9NYF8	BCLAF1	Bcl-2-associated transcription factor 1 [OS=Homo sapiens]	[881-891]	-2.08
Q9H7N4	SCAF1	Splicing factor, arginine/serine-rich 19 [OS=Homo sapiens]	T976	-2.07

Q15149-4	PLEC	Isoform 4 of Plectin [OS=Homo sapiens]	[18-31]	-2.07
Q7KZI7-14	MARK2	Isoform 14 of Serine/threonine-protein kinase MARK2 [OS=Homo sapiens]	[561-569]	-2.06
Q86WB0	ZC3HC1	Zinc finger C3HC-type protein 1 [OS=Homo sapiens]	S354; S370	-2.06
O43237	DYNC1LI2	Cytoplasmic dynein 1 light intermediate chain 2 [OS=Homo sapiens]	S194	-2.05
Q9ULW0	TPX2	Targeting protein for Xklp2 [OS=Homo sapiens]	S486	-2.04
Q9Y2W1	THRAP3	Thyroid hormone receptor-associated protein 3 [OS=Homo sapiens]	S55	-2.04
Q9UK58	CCNL1	Cyclin-L1 [OS=Homo sapiens]	S352	-2.04
P49790	NUP153	Nuclear pore complex protein Nup153 [OS=Homo sapiens]	S614	-2.04
P46087	NOP2	Probable 28S rRNA (cytosine(4447)-C(5))-methyltransferase [OS=Homo sapiens]	S732	-2.04
Q9H7D7	WDR26	WD repeat-containing protein 26 [OS=Homo sapiens]	S121	-2.04
Q13427	PPIG	Peptidyl-prolyl cis-trans isomerase G [OS=Homo sapiens]	S687	-2.03
*O94804	STK10	Serine/threonine-protein kinase 10 [OS=Homo sapiens]	T58	-2.02
Q13428-3	TCOF1	Isoform 3 of Treacle protein [OS=Homo sapiens]	S1379	-2.01
Q13428-7	TCOF1	Isoform 7 of Treacle protein [OS=Homo sapiens]	S1341	-2.01
Q13330	MTA1	Metastasis-associated protein MTA1 [OS=Homo sapiens]	S576	-2.01
Q13428-6	TCOF1	Isoform 6 of Treacle protein [OS=Homo sapiens]	S1340	-2.00
Q86VM9	ZC3H18	Zinc finger CCCH domain-containing protein 18 [OS=Homo sapiens]	S534	-2.00
Q8NEY8	PPHLN1	Periphilin-1 [OS=Homo sapiens]	S155	-2.00
Q13428-7	TCOF1	Isoform 7 of Treacle protein [OS=Homo sapiens]	S156	-1.99
P04049	RAF1	RAF proto-oncogene serine/threonine-protein kinase [OS=Homo sapiens]	S43	-1.99
P35658	NUP214	Nuclear pore complex protein Nup214 [OS=Homo sapiens]	S433; T434	-1.98
Q13428-3	TCOF1	Isoform 3 of Treacle protein [OS=Homo sapiens]	S156	-1.98
P16070	CD44	CD44 antigen [OS=Homo sapiens]	[695-715]	-1.98
Q8N556	AFAP1	Actin filament-associated protein 1 [OS=Homo sapiens]	[246-275]	-1.98
*Q15121	PEA15	Astrocytic phosphoprotein PEA-15 [OS=Homo sapiens]	S116	-1.97
Q13428-6	TCOF1	Isoform 6 of Treacle protein [OS=Homo sapiens]	S156	-1.97
*O14737	PDCD5	Programmed cell death protein 5 [OS=Homo sapiens]	S119 [116-125]	-1.97
*Q86UE4	MTDH	Protein LYRIC [OS=Homo sapiens]	S568	-1.97
Q86SQ0	PHLDB2	Pleckstrin homology-like domain family B member 2 [OS=Homo sapiens]	T898	-1.96
Q8IYB3	SRRM1	Serine/arginine repetitive matrix protein 1 [OS=Homo sapiens]	S675	-1.96
Q53F19	NCBP3	Nuclear cap-binding protein subunit 3 [OS=Homo sapiens]	S500	-1.95
Q9H501	ESF1	ESF1 homolog [OS=Homo sapiens]	S153	-1.95
Q8IYB3	SRRM1	Serine/arginine repetitive matrix protein 1 [OS=Homo sapiens]	T220	-1.95
Q53GS9	USP39	U4/U6.U5 tri-snRNP-associated protein 2 [OS=Homo sapiens]	S82 [75-89]	-1.95
*O94804	STK10	Serine/threonine-protein kinase 10 [OS=Homo sapiens]	S13	-1.95
O95425-2	SVIL	Isoform 2 of Supervillin [OS=Homo sapiens]	S626	-1.95
Q9NR19	ACSS2	Acetyl-coenzyme A synthetase, cytoplasmic [OS=Homo sapiens]	S267	-1.94
*Q14004	CDK13	Cyclin-dependent kinase 13 [OS=Homo sapiens]	T588	-1.94
P26358	DNMT1	DNA (cytosine-5)-methyltransferase 1 [OS=Homo sapiens]	[141-156]	-1.93
Q92576	PHF3	PHD finger protein 3 [OS=Homo sapiens]	S1133	-1.93

Q13428-7	TCOF1	Isoform 7 of Treacle protein [OS=Homo sapiens]	T249	-1.93
Q8WXF7	ATL1	Atlastin-1 [OS=Homo sapiens]	S10	-1.93
Q9UKV3	ACIN1	Apoptotic chromatin condensation inducer in the nucleus [OS=Homo sapiens]	S1004	-1.92
*Q05209	PTPN12	Tyrosine-protein phosphatase non-receptor type 12 [OS=Homo sapiens]	S449	-1.92
Q8IYB3	SRRM1	Serine/arginine repetitive matrix protein 1 [OS=Homo sapiens]	S638	-1.92
Q13428-3	TCOF1	Isoform 3 of Treacle protein [OS=Homo sapiens]	T249	-1.92
Q13428-6	TCOF1	Isoform 6 of Treacle protein [OS=Homo sapiens]	T249	-1.91
Q8TAD8	SNIP1	Smad nuclear-interacting protein 1 [OS=Homo sapiens]	S52; S54	-1.91
Q9BRG2	SH2D3A	SH2 domain-containing protein 3A [OS=Homo sapiens]	S125	-1.90
P54259	ATN1	Atrophin-1 [OS=Homo sapiens]	S34	-1.89
Q04637	EIF4G1	Eukaryotic translation initiation factor 4 gamma 1 [OS=Homo sapiens]	S1092	-1.88
*Q9ULH1	ASAP1	Arf-GAP with SH3 domain, ANK repeat and PH domain-containing protein 1 [OS=Homo sapiens]	S1008	-1.88
*Q13263	TRIM28	Transcription intermediary factor 1-beta [OS=Homo sapiens]	S473	-1.88
Q15648	MED1	Mediator of RNA polymerase II transcription subunit 1 [OS=Homo sapiens]	S1156	-1.87
*P49585	PCYT1A	Choline-phosphate cytidylyltransferase A [OS=Homo sapiens]	S362 [354-367]	-1.87
*Q9C0C2	TNKS1BP1	182 kDa tankyrase-1-binding protein [OS=Homo sapiens]	S1545	-1.87
Q52LW3	ARHGAP29	Rho GTPase-activating protein 29 [OS=Homo sapiens]	[947-962]	-1.87
Q96PK6	RBM14	RNA-binding protein 14 [OS=Homo sapiens]	T206	-1.87
Q13247	SRSF6	Serine/arginine-rich splicing factor 6 [OS=Homo sapiens]	S314; S316	-1.86
P26368	U2AF2	Splicing factor U2AF 65 kDa subunit [OS=Homo sapiens]	S79	-1.86
Q00839	HNRNPU	Heterogeneous nuclear ribonucleoprotein U [OS=Homo sapiens]	S26	-1.85
*Q9C0C2	TNKS1BP1	182 kDa tankyrase-1-binding protein [OS=Homo sapiens]	S1029	-1.85
Q00839-2	HNRNPU	Isoform 2 of Heterogeneous nuclear ribonucleoprotein U [OS=Homo sapiens]	S26	-1.84
Q8IYB3	SRRM1	Serine/arginine repetitive matrix protein 1 [OS=Homo sapiens]	S560; S562	-1.84
O96013	PAK4	Serine/threonine-protein kinase PAK 4 [OS=Homo sapiens]	S104; S99	-1.83
*O00515	LAD1	Ladinin-1 [OS=Homo sapiens]	S177	-1.83
Q99607	ELF4	ETS-related transcription factor Elf-4 [OS=Homo sapiens]	S188	-1.83
Q9Y3T9	NOC2L	Nucleolar complex protein 2 homolog [OS=Homo sapiens]	S49; S56	-1.82
Q9BZE4	GTPBP4	GTP-binding protein 4 [OS=Homo sapiens]	S558	-1.81
*Q15773	MLF2	Myeloid leukemia factor 2 [OS=Homo sapiens]	S238	-1.81
P78347	GTF2I	General transcription factor II-I [OS=Homo sapiens]	[807-826]	-1.81
Q9Y2W1	THRAP3	Thyroid hormone receptor-associated protein 3 [OS=Homo sapiens]	S919; S243	-1.81
*Q9UHB6	LIMA1	LIM domain and actin-binding protein 1 [OS=Homo sapiens]	S362	-1.80
*Q12929	EPS8	Epidermal growth factor receptor kinase substrate 8 [OS=Homo sapiens]	T223	-1.79
Q07157-2	TJP1	Isoform Short of Tight junction protein ZO-1 [OS=Homo sapiens]	S1490	-1.79
P02545	LMNA	Prelamin-A/C [OS=Homo sapiens]	S458	-1.78
Q07157	TJP1	Tight junction protein ZO-1 [OS=Homo sapiens]	S1570	-1.78
Q9C0B5	ZDHHC5	Palmitoyltransferase ZDHHC5 [OS=Homo sapiens]	[583-597]	-1.78

P02545-2	LMNA	Isoform C of Prelamin-A/C [OS=Homo sapiens]	S458	-1.77
Q9UKV3	ACIN1	Apoptotic chromatin condensation inducer in the nucleus [OS=Homo sapiens]	S216 [205-22]	-1.77
Q8TF01	PNISR	Arginine/serine-rich protein PNISR [OS=Homo sapiens]	[286-307]	-1.77
*Q9UDY2	TJP2	Tight junction protein ZO-2 [OS=Homo sapiens]	S986 [975-997]	-1.77
*Q8TDD1	DDX54	ATP-dependent RNA helicase DDX54 [OS=Homo sapiens]	S782	-1.77
Q9Y232	CDYL	Chromodomain Y-like protein [OS=Homo sapiens]	S149	-1.76
Q13177	PAK2	Serine/threonine-protein kinase PAK 2 [OS=Homo sapiens]	S141	-1.76
Q9H4A3	WNK1	Serine/threonine-protein kinase WNK1 [OS=Homo sapiens]	[366-381]	-1.75
*Q9UDY2	TJP2	Tight junction protein ZO-2 [OS=Homo sapiens]	S174	-1.75
Q7Z460-4	CLASP1	Isoform 4 of CLIP-associating protein 1 [OS=Homo sapiens]	S738	-1.75
Q7Z460-3	CLASP1	Isoform 3 of CLIP-associating protein 1 [OS=Homo sapiens]	S731	-1.74
Q9Y2W1	THRAP3	Thyroid hormone receptor-associated protein 3 [OS=Homo sapiens]	S408 [397-410]	-1.74
Q8IYB3	SRRM1	Serine/arginine repetitive matrix protein 1 [OS=Homo sapiens]	S769; S463	-1.74
Q9P206	KIAA1522	Uncharacterized protein KIAA1522 [OS=Homo sapiens]	S545	-1.73
Q14247	CTTN	Src substrate cortactin [OS=Homo sapiens]	T401	-1.73
Q8IYB3	SRRM1	Serine/arginine repetitive matrix protein 1 [OS=Homo sapiens]	S874	-1.72
*Q4G0J3	LARP7	La-related protein 7 [OS=Homo sapiens]	S300	-1.72
Q9Y2D5	PALM2AKAP2	A-kinase anchor protein 2 [OS=Homo sapiens]	S778	-1.72
*Q9UPT8	ZC3H4	Zinc finger CCCH domain-containing protein 4 [OS=Homo sapiens]	S1275	-1.72
Q15149-4	PLEC	Isoform 4 of Plectin [OS=Homo sapiens]	S4249; S4252	-1.72
P25788	PSMA3	Proteasome subunit alpha type-3 [OS=Homo sapiens]	S250	-1.72
Q66K74	MAP1S	Microtubule-associated protein 1S [OS=Homo sapiens]	S582	-1.72
*O14737	PDCD5	Programmed cell death protein 5 [OS=Homo sapiens]	S119 [114-125]	-1.72
P49736	MCM2	DNA replication licensing factor MCM2 [OS=Homo sapiens]	T25	-1.71
Q96ST3	SIN3A	Paired amphipathic helix protein Sin3a [OS=Homo sapiens]	S1112	-1.71
Q9NQW6	ANLN	Anillin [OS=Homo sapiens]	S295	-1.71
Q04637	EIF4G1	Eukaryotic translation initiation factor 4 gamma 1 [OS=Homo sapiens]	T205	-1.71
*Q07002	CDK18	Cyclin-dependent kinase 18 [OS=Homo sapiens]	S98	-1.69
Q9UQ35	SRRM2	Serine/arginine repetitive matrix protein 2 [OS=Homo sapiens]	S1601	-1.69
Q9C0B5	ZDHHC5	Palmitoyltransferase ZDHHC5 [OS=Homo sapiens]	S299	-1.69
*Q9UDY2	TJP2	Tight junction protein ZO-2 [OS=Homo sapiens]	S986 [976-997]	-1.69
P02545	LMNA	Prelamin-A/C [OS=Homo sapiens]	T19	-1.68
Q05682-4	CALD1	Isoform 4 of Caldesmon [OS=Homo sapiens]	T533	-1.68
P29692-3	EEF1D	Isoform 3 of Elongation factor 1-delta [OS=Homo sapiens]	S109	-1.68
P29692	EEF1D	Elongation factor 1-delta [OS=Homo sapiens]	S133	-1.67
*Q7Z4V5	HDGFL2	Hepatoma-derived growth factor-related protein 2 [OS=Homo sapiens]	S490	-1.67
Q71RC2	LARP4	La-related protein 4 [OS=Homo sapiens]	S722	-1.67
P02545	LMNA	Prelamin-A/C [OS=Homo sapiens]	S18	-1.66
Q14244	MAP7	Ensconsin [OS=Homo sapiens]	S202	-1.66
Q13523	PRP4K	Serine/threonine-protein kinase PRP4 homolog [OS=Homo sapiens]	S166	-1.66
O15231-3	ZNF185	Isoform 3 of Zinc finger protein 185 [OS=Homo sapiens]	S520	-1.66

Q92890-1	UFD1	Isoform Long of Ubiquitin recognition factor in ER-associated degradation protein 1 [OS=Homo sapiens]	S283	-1.65
P61978-2	HNRNPK	Isoform 2 of Heterogeneous nuclear ribonucleoprotein K [OS=Homo sapiens]	S284	-1.65
P02545-2	LMNA	Isoform C of Prelamin-A/C [OS=Homo sapiens]	T19	-1.65
Q6ZRV2	FAM83H	Protein FAM83H [OS=Homo sapiens]	S523	-1.65
P19338	NCL	Nucleolin [OS=Homo sapiens]	S67 [63-71]	-1.65
Q05655	PRKCD	Protein kinase C delta type [OS=Homo sapiens]	S304	-1.65
*P28290	ITPRID2	Protein ITPRID2 [OS=Homo sapiens]	S739	-1.65
P46087	NOP2	Probable 28S rRNA (cytosine(4447)-C(5))-methyltransferase [OS=Homo sapiens]	S67	-1.64
*Q07002	CDK18	Cyclin-dependent kinase 18 [OS=Homo sapiens]	S14	-1.64
P02545-2	LMNA	Isoform C of Prelamin-A/C [OS=Homo sapiens]	S18	-1.64
Q8TEW0	PARD3	Partitioning defective 3 homolog [OS=Homo sapiens]	S1178	-1.63
*P18615	NELFE	Negative elongation factor E [OS=Homo sapiens]	S181	-1.63
Q6Y7W6	GIGYF2	GRB10-interacting GYF protein 2 [OS=Homo sapiens]	T382	-1.63
A7KAX9	ARHGAP32	Rho GTPase-activating protein 32 [OS=Homo sapiens]	S1796	-1.62
Q9BST9	RTKN	Rhotekin [OS=Homo sapiens]	S520; S529	-1.61
Q53EL6	PDCD4	Programmed cell death protein 4 [OS=Homo sapiens]	S457	-1.61
*P49585	PCYT1A	Choline-phosphate cytidylyltransferase A [OS=Homo sapiens]	S362 [356-367]	-1.61
Q9BVG9	PTDSS2	Phosphatidylserine synthase 2 [OS=Homo sapiens]	S16	-1.60
Q2M2I8	AAK1	AP2-associated protein kinase 1 [OS=Homo sapiens]	S678	-1.60
P09651	HNRNPA1	Heterogeneous nuclear ribonucleoprotein A1 [OS=Homo sapiens]	S6	-1.60
Q9H8Y8	GORASP2	Golgi reassembly-stacking protein 2 [OS=Homo sapiens]	T433	-1.60
Q8WUI4	HDAC7	Histone deacetylase 7 [OS=Homo sapiens]	[484-504]	-1.60
Q6PKG0	LARP1	La-related protein 1 [OS=Homo sapiens]	S766; S774	-1.59
Q14978	NOLC1	Nucleolar and coiled-body phosphoprotein 1 [OS=Homo sapiens]	S538	-1.59
Q9UQ35	SRRM2	Serine/arginine repetitive matrix protein 2 [OS=Homo sapiens]	[1394-1418]	-1.59
Q9H2G2	SLK	STE20-like serine/threonine-protein kinase [OS=Homo sapiens]	S372	-1.58
O60333	KIF1B	Kinesin-like protein KIF1B [OS=Homo sapiens]	S1454	-1.58
Q9BW04	SARG	Specifically androgen-regulated gene protein [OS=Homo sapiens]	S133	-1.57
O75369-8	FLNB	Isoform 8 of Filamin-B [OS=Homo sapiens]	S1536	-1.57
*Q6ZN18	AEBP2	Zinc finger protein AEBP2 [OS=Homo sapiens]	S24	-1.57
Q8IU81	IRF2BP1	Interferon regulatory factor 2-binding protein 1 [OS=Homo sapiens]	S384	-1.57
P28749	PWP1	Retinoblastoma-like protein 1 [OS=Homo sapiens]	S640	-1.57
*Q13610	RBL1	Periodic tryptophan protein 1 homolog [OS=Homo sapiens]	S50	-1.57
Q9BTU6	PI4K2A	Phosphatidylinositol 4-kinase type 2-alpha [OS=Homo sapiens]	S47; S51	-1.56
*P35579	MYH9	Myosin-9 [OS=Homo sapiens]	S1943 [1937-1960]	-1.56
P35611	ADD1	Alpha-adducin [OS=Homo sapiens]	S726	-1.56
Q9UEY8	ADD3	Gamma-adducin [OS=Homo sapiens]	S693	-1.56
Q86VM9	ZC3H18	Zinc finger CCCH domain-containing protein 18 [OS=Homo sapiens]	[833-847]	-1.56

*Q8NC56	LEMD2	LEM domain-containing protein 2 [OS=Homo sapiens]	S138; S139	-1.56
*Q96QC0	PPP1R10	Serine/threonine-protein phosphatase 1 regulatory subunit 10 [OS=Homo sapiens]	S313	-1.55
*Q8TB61	SLC35B2	Adenosine 3'-phospho 5'-phosphosulfate transporter 1 [OS=Homo sapiens]	S427	-1.55
*Q7Z6Z7	HUWE1	E3 ubiquitin-protein ligase HUWE1 [OS=Homo sapiens]	T2889	-1.55
P51532	SMARCA4	Transcription activator BRG1 [OS=Homo sapiens]	S1452	-1.54
Q16555	DPYSL2	Dihydropyrimidinase-related protein 2 [OS=Homo sapiens]	T514	-1.54
Q8NC51	SERBP1	Plasminogen activator inhibitor 1 RNA-binding protein [OS=Homo sapiens]	S234	-1.53
*Q07352	ZFP36L1	mRNA decay activator protein ZFP36L1 [OS=Homo sapiens]	S92	-1.52
*Q9BY44	EIF2A	Eukaryotic translation initiation factor 2A [OS=Homo sapiens]	S526	-1.52
Q9BSQ5	CCM2	Cerebral cavernous malformations 2 protein [OS=Homo sapiens]	S384	-1.52
Q53EL6	PDCD4	Programmed cell death protein 4 [OS=Homo sapiens]	S76	-1.52
Q8IZ21	PHACTR4	Phosphatase and actin regulator 4 [OS=Homo sapiens]	S590	-1.51
*Q8NEY1	NAV1	Neuron navigator 1 [OS=Homo sapiens]	S672	-1.51
*Q5UIP0	RIF1	Telomere-associated protein RIF1 [OS=Homo sapiens]	S2161	-1.51
Q09666	AHNAK	Neuroblast differentiation-associated protein AHNAK [OS=Homo sapiens]	[5834-5859]	-1.51
Q9UBF8-2	PI4KB	Isoform 2 of Phosphatidylinositol 4-kinase beta [OS=Homo sapiens]	S277	-1.50
Q86WR7	PROSER2	Proline and serine-rich protein 2 [OS=Homo sapiens]	S179	-1.50
Q13428-6	TCOF1	Isoform 6 of Treacle protein [OS=Homo sapiens]	T1232	-1.50
P05455	SSB	Lupus La protein [OS=Homo sapiens]	[362-383]	-1.49
*Q5T0Z8	C6orf132	Uncharacterized protein C6orf132 [OS=Homo sapiens]	S1011	-1.49
Q96JP5	ZFP91	E3 ubiquitin-protein ligase ZFP91 [OS=Homo sapiens]	S103	-1.49
P48634	PRRC2A	Protein PRRC2A [OS=Homo sapiens]	T1347	-1.49
Q9NQW6	ANLN	Anillin [OS=Homo sapiens]	S323	-1.49
Q8WUI4	HDAC7	Histone deacetylase 7 [OS=Homo sapiens]	S155	-1.49
Q8N3D4	EHBP1L1	EH domain-binding protein 1-like protein 1 [OS=Homo sapiens]	S310	-1.49
P62995	TRA2B	Transformer-2 protein homolog beta [OS=Homo sapiens]	S264; S266	-1.48
Q6PKG0	LARP1	La-related protein 1 [OS=Homo sapiens]	T526; T724	-1.48
P27816	MAP4	Microtubule-associated protein 4 [OS=Homo sapiens]	S280	-1.48
Q9UQ35	SRRM2	Serine/arginine repetitive matrix protein 2 [OS=Homo sapiens]	S2030; S2032; T2034	-1.48
Q13428-7	TCOF1	Isoform 7 of Treacle protein [OS=Homo sapiens]	T1233	-1.47
*Q9UMZ2	SYNRG	Synergin gamma [OS=Homo sapiens]	T1100	-1.47
Q9Y2W1	THRAP3	Thyroid hormone receptor-associated protein 3 [OS=Homo sapiens]	S408 [397-411]	-1.47
*Q9UHB6	LIMA1	LIM domain and actin-binding protein 1 [OS=Homo sapiens]	S374	-1.47
O15027	SEC16A	Protein transport protein Sec16A [OS=Homo sapiens]	S314	-1.47
O95425-2	SVIL	Isoform 2 of Supervillin [OS=Homo sapiens]	S221	-1.46
Q13595	TRA2A	Transformer-2 protein homolog alpha [OS=Homo sapiens]	S260; S262	-1.46
Q13428-3	TCOF1	Isoform 3 of Treacle protein [OS=Homo sapiens]	T1271	-1.46
P49790	NUP153	Nuclear pore complex protein Nup153 [OS=Homo sapiens]	S334	1.49
P31350	RRM2	Ribonucleoside-diphosphate reductase subunit M2 [OS=Homo sapiens]	S20	1.52

P27816	MAP4	Microtubule-associated protein 4 [OS=Homo sapiens]	T521	1.91
Q8IYB3	SRRM1	Serine/arginine repetitive matrix protein 1 [OS=Homo sapiens]	S696	2.03
O15231-3	ZNF185	Isoform 3 of Zinc finger protein 185 [OS=Homo sapiens]	[497-529]	2.08
Q8IY67-2	RAVER1	Isoform 2 of Ribonucleoprotein PTB-binding 1 [OS=Homo sapiens]	S617	2.13
Q8IYB3	SRRM1	Serine/arginine repetitive matrix protein 1 [OS=Homo sapiens]	S414	2.18
Q9UQ35	SRRM2	Serine/arginine repetitive matrix protein 2 [OS=Homo sapiens]	S1132	2.26
P46937	YAP1	Transcriptional coactivator YAP1 [OS=Homo sapiens]	S367	2.26
Q99590	SCAF11	Protein SCAF11 [OS=Homo sapiens]	[329-366]	2.28
Q14978	NOLC1	Nucleolar and coiled-body phosphoprotein 1 [OS=Homo sapiens]	S623	2.29
Q9BXP5	SRRT	Serrate RNA effector molecule homolog [OS=Homo sapiens]	S67; S74	2.31
Q53GS9	USP39	U4/U6.U5 tri-snRNP-associated protein 2 [OS=Homo sapiens]	S82 [77-89]	2.33
Q15424	SAFB	Scaffold attachment factor B1 [OS=Homo sapiens]	S383	2.36
Q07955	SRSF1	Serine/arginine-rich splicing factor 1 [OS=Homo sapiens]	S199	2.36
Q00587	CDC42EP1	Cdc42 effector protein 1 [OS=Homo sapiens]	S121	2.38
P49792	RANBP2	E3 SUMO-protein ligase RanBP2 [OS=Homo sapiens]	S3207	2.42
Q8IYB3	SRRM1	Serine/arginine repetitive matrix protein 1 [OS=Homo sapiens]	S562	2.44
Q96T58	SPEN	Msx2-interacting protein [OS=Homo sapiens]	S736/S740	2.45
Q9Y6N7	ROBO1	Roundabout homolog 1 [OS=Homo sapiens]	S940	2.48
Q9UQ35	SRRM2	Serine/arginine repetitive matrix protein 2 [OS=Homo sapiens]	S2132	2.56
Q9UQ35	SRRM2	Serine/arginine repetitive matrix protein 2 [OS=Homo sapiens]	[1441-1456]	2.60
Q6WCQ1-2	MPRIP	Isoform 2 of Myosin phosphatase Rho-interacting protein [OS=Homo sapiens]	S619	2.61
P16144	ITGB4	Integrin beta-4 [OS=Homo sapiens]	S1474	2.61
Q86YV5	PRAG1	Inactive tyrosine-protein kinase PRAG1 [OS=Homo sapiens]	[138-154]	2.69
Q86VM9	ZC3H18	Zinc finger CCCH domain-containing protein 18 [OS=Homo sapiens]	[529-539]	2.82
O75367	MACROH2A1	Core histone macro-H2A.1 [OS=Homo sapiens]	T129 [122-134]	2.94
Q9UQ35	SRRM2	Serine/arginine repetitive matrix protein 2 [OS=Homo sapiens]	S2581	2.94
P06748-3	NPM1	Isoform 3 of Nucleophosmin [OS=Homo sapiens]	T199	3.26
P06748	NPM1	Nucleophosmin [OS=Homo sapiens]	T199	3.27
P02545-2	LMNA	Isoform C of Prelamin-A/C [OS=Homo sapiens]	S390	4.20
P02545	LMNA	Prelamin-A/C [OS=Homo sapiens]	S390	4.21
Q14160	SCRIB	Protein scribble homolog [OS=Homo sapiens]	[1536-1574]	5.60
Q7L7X3	TAOK1	Serine/threonine-protein kinase TAO1 [OS=Homo sapiens]	S965	5.67
Q5VTL8	PRPF38B	Pre-mRNA-splicing factor 38B [OS=Homo sapiens]	S527; S529	5.68
Q9UQ35	SRRM2	Serine/arginine repetitive matrix protein 2 [OS=Homo sapiens]	[987-998]	6.23
Q9ULM3	YEATS2	YEATS domain-containing protein 2 [OS=Homo sapiens]	S465	6.27
Q6WKZ4	RAB11FIP1	Rab11 family-interacting protein 1 [OS=Homo sapiens]	S435	6.27
Q13586	STIM1	Stromal interaction molecule 1 [OS=Homo sapiens]	S618	6.30
Q9BST9	RTKN	Rhotekin [OS=Homo sapiens]	S543	6.31
Q7RTP6	MICAL3	[F-actin]-monooxygenase MICAL3 [OS=Homo sapiens]	S1704	6.42
Q96JP5	ZFP91	E3 ubiquitin-protein ligase ZFP91 [OS=Homo sapiens]	[81-108]	6.42
Q6IQ22	RAB12	Ras-related protein Rab-12 [OS=Homo sapiens]	S21 [11-31]	6.43
Q6PKG0	LARP1	La-related protein 1 [OS=Homo sapiens]	S143	6.48
Q03164	KMT2A	Histone-lysine N-methyltransferase 2A [OS=Homo sapiens]	S3036	6.52

Q13541	EIF4EBP1	Eukaryotic translation initiation factor 4E-binding protein 1 [OS=Homo sapiens]	[21-57]	6.67
P38159	RBMX	RNA-binding motif protein, X chromosome [OS=Homo sapiens]	[318-339]	6.67
Q8TF01	PNISR	Arginine/serine-rich protein PNISR [OS=Homo sapiens]	S211	6.73
Q5T200	ZC3H13	Zinc finger CCCH domain-containing protein 13 [OS=Homo sapiens]	S207	6.73
Q9Y2W1	THRAP3	Thyroid hormone receptor-associated protein 3 [OS=Homo sapiens]	[182-215]	6.74
Q9UJU6	DBNL	Drebrin-like protein [OS=Homo sapiens]	S232	6.74
Q01130	SRSF2	Serine/arginine-rich splicing factor 2 [OS=Homo sapiens]	[25-32]	6.86
