# Supplementary material for: SCAMP3-Driven Regulation of ERK1/2 and Autophagy Phosphoproteomics Signatures in Triple-Negative Breast Cancer
Source: Int J Mol Sci. 2025 Oct 1;26(19):9577. doi: 10.3390/ijms26199577 (PMC12525412; doi:10.3390/ijms26199577)
Supplement: Supplementary file 1 [file ijms-26-09577-s001.zip › Table S3.pdf]

Table S3. ERK1/2 targets identified as deregulated in the NT: SC3KO vs WT comparison. The table lists ERK targets deregulated in the phosphoproteomics analysis based on datasets from https://sys-bio.net/erk_targets/targets_all.html and PhosphoSitePlus®.

Accession No.	Gene	Description	Phosphosite	log2(FC)[1]
Q5VZK9	CARMIL1	F-actin-uncapping protein LRRC16A	S1291 ↓	-3.10
Q9Y2D5	PALM2AKAP2	A-kinase anchor protein 2	S748 ↓	-3.08
P54727	RAD23B	UV excision repair protein RAD23B	S160 ↓	-2.97
Q7Z417	NUFIP2	Nuclear fragile X mental retardation-interacting protein 2	S629 ↓	-2.72
Q5T200	ZC3H13	Zinc finger CCCH domain-containing protein 13	T364, S77 ↓	-2.68
Q09666	AHNAK	Neuroblast differentiation-associated protein AHNAK	S3426 ↓	-2.63
P29966	MARCKS	Myristoylated alanine-rich C-kinase substrate	S170 ↓	-2.59
Q16181	SEPTIN7	Septin-7	T426 ↓	-2.49
Q9ULJ3	ZBTB21	Zinc finger and BTB domain-containing protein 21	S411 ↓	-2.45
O95239	KIF4A	Chromosome-associated kinesin KIF4A	S801 ↓	-2.41
Q9Y2W1	THRAP3	Thyroid hormone receptor-associated protein 3	S672 ↓	-2.41
Q92615	LARP4B	La-related protein 4B	S731, S736 ↓	-2.34
P02545	LMNA	Prelamin-A/C	S458 ↓	-2.27
P55196	AFDN	Afadin	S1721 ↓	-2.19
Q9NZN8	CNOT2	CCR4-NOT transcription complex subunit 2	S165 ↓	-2.13
Q9UK58	CCNL1	Cyclin-L1	S352 ↓	-2.11
Q9NYF8	BCLAF1	Bcl-2-associated transcription factor 1	S496 ↓	-2.09
Q13439	GOLGA4	Golgin subfamily A member 4	S71 ↓, S41↑	-2.02, +2.32
Q14247	CTTN	Src substrate cortactin	T401 ↓	-1.93
P48634	PRRC2A	Proline-rich and coiled-coil-containing protein 2A	S761 ↓	-1.80

Q8WXF7	ATL1	Atlastin-1	S10 ↓	-1.68
Q9NZT2	OGFR	Opioid growth factor receptor	S378 ↓	-1.65
Q3KQU3	MAP7D1	MAP7 domain-containing protein 1	S116 ↓	-1.60
Q9BQG0	MYBBP1A	Myb-binding protein 1A	S1267 ↓	-1.52
Q9Y3Q8	TSC22D4	TSC22 domain family protein 4	T229 ↓	-1.52
Q96D71	REPS1	RalBP1-associated Eps domain-containing protein 1	S709 ↓	-1.46
O95425-2	SVIL	Isoform 2 of Supervillin	S221 ↓	-1.46
Q92597	NDRG1	Protein NDRG1	S333, S336 ↑	+2.19
Q96E09	PABIR1	PPP2R1A-PPP2R2A-interacting phosphatase regulator	S76 ↑	+2.14
Q7Z309-3	PABIR2	Isoform 3 of PABIR family member 2	S25 ↑	+2.11

[1] Negative values indicate downregulated phosphosites and are marked with blue arrows, while positive values indicate upregulated phosphosites and are marked with orange arrows. Phosphoproteins in the table are ordered from the most downregulated to the least downregulated, followed by the most upregulated to the least upregulated.
