# Supplementary material for: SCAMP3-Driven Regulation of ERK1/2 and Autophagy Phosphoproteomics Signatures in Triple-Negative Breast Cancer
Source: Int J Mol Sci. 2025 Oct 1;26(19):9577. doi: 10.3390/ijms26199577 (PMC12525412; doi:10.3390/ijms26199577)
Supplement: Supplementary file 1 [file ijms-26-09577-s001.zip › Table S4.pdf]

Table S4: Ranking and associated substrates for the enriched kinases.

Rank	Protein	Mean rank	Substrates
**Hypophosphorylated proteins**			
1	CSNK2A1	10.64	TOP2A,HSP90AB1,PRAG1,NUMA1,RIF1,POP1,CHD4,SMC3,MKI67,EDC4,GOLGA4,SLK,MPRIP,RAVER1,TRIM29,BBX,TNKS1BP1,KIF1C,KIF1B,CCNL1,AGFG1,SRRM2,MED1,EIF5B,SMARCC1,MAP2K2,SCAF11,ZFP91,AFAP1,LMO7,LARP4B,RAD23B,UTP18,NCOR2,ACLY,DYNC1LI2,PSMA3,NCOR1,STIM1,CTTN,THRAP3,CANX,ARHGEF2,RAF1,SQSTM1,S100A9,PHLDB2,PLEC,ANKRD17,KMT2A,AHNAK,YTHDC1,ASAP1,PDS5B,KLC4,BCLAF1,RBM15B,HSPH1,POLR2A,TCOF1,CALD1,LMNA,TPR,RPS3,ZC3H18,RBBP6,HNRNPA1,ZC3H13,MEF2D,SPTBN1,FAM83B,REPS1,CARMIL1,HMGA1,HUWE1,MICAL3,IRF2BP2,LARP7,PDAP1,PRRC2C,ARHGAP29,NUP153,L1CAM,AFDN,ABI2,MYBBP1A,RAB12,CHMP2B,ACIN1,ESF1,RBMX,EIF4G1
2	CSNK2A2	16.9	TOP2A,HSP90AB1,NUMA1,RIF1,POP1,CHD4,SMC3,SLK,KIF1C,BBX,KIF1B,CCNL1,AGFG1,SRRM2,EIF5B,SMARCC1,MAP2K2,ZFP91,AFAP1,RAD23B,UTP18,NCOR2,PSMA3,NCOR1,CTTN,THRAP3,ARHGEF2,RAF1,SQSTM1,PHLDB2,S100A9,KMT2A,ASAP1,PDS5B,KLC4,BCLAF1,HSPH1,POLR2A,TCOF1,RPS3,ZC3H18,HNRNPA1,MEF2D,SPTBN1,REPS1,HMGA1,HUWE1,MICAL3,PRRC2C,LARP7,NUP153,L1CAM,AFDN,MYBBP1A,ACIN1,ESF1,RBMX
3	CDK9	27.55	TOP2A,HSP90AB1,KMT2A,CHD4,PDS5B,SMC3,KLC4,BCLAF1,SLK,POLR2A,RAVER1,TCOF1,LMNA,TPR,RPS3,ZC3H18,RBBP6,CCNL1,ZC3H13,HNRNPA1,MEF2D,REPS1,NUP214,MED1,CARMIL1,SMARCC1,MAP2K2,HMGA1,HUWE1,LARP7,RAD23B,NCOR1,MYBBP1A,THRAP3,CANX,RAF1,SQSTM1,S100A9,PLEC,EIF4G1
4	MAPK1	30.18	TOP2A,HSP90AB1,NUMA1,RIF1,ATN1,CHD4,SMC3,MKI67,ROBO1,EDC4,GOLGA4,SLK,MPRIP,RAVER1,TRIM29,KIF1C,KIF1B,CCNL1,AGFG1,RALGPS2,NUP214,MED1,EIF5B,SMARCC1,MAP2K2,SCAF11,ZFP91,AFAP1,LMO7,RAD23B,UTP18,NCOR2,ACLY,DYNC1LI2,PSMA3,NCOR1,STIM1,CTTN,PALLD,CANX,ARHGEF2,RAF1,SQSTM1,S100A9,PHLDB2,OPTN,PLEC,ANKRD17,KMT2A,AHNAK,ASAP1,PDS5B,NDRG1,KLC4,BCLAF1,HSPH1,POLR2A,TCOF1,CALD1,TPR,LMNA,RPS3,RBBP6,HNRNPA1,MEF2D,SPTBN1,HUWE1,ARHGAP29,LARP7,NUP153,L1CAM,AFDN,MARCKS,ABI2,MYBBP1A,RAB12,CNOT2,CHMP2B,ACIN1,RBMX,EIF4G1

5	CDK2	36	TOP2A,HSP90AB1,NUMA1,PRAG1,RIF1,NUCKS1,CHD4,MKI67,SMC3,ROBO1,EIF4ENIF1,GOLGA4,SLK,MPRIP,TNKS1BP1,KIF1C,KIF21A,KIF1B,CCNL1,SRRM2,NUP214,MED1,EIF5B,SMARCC1,MAP2K2,TPI1,AFAP1,LMO7,KIF23,LARP4B,RAD23B,NCOR2,ACLY,PSMA3,NCOR1,CTTN,THRAP3,CANX,RAF1,PHLDB2,SQSTM1,OPTN,PLEC,ANKRD17,CDCA3,AHNAK,KMT2A,NUFIP2,ASAP1,PDS5B,NDRG1,KLC4,BCLAF1,HSPH1,POLR2A,TCOF1,CALD1,TPR,LMNA,RPS3,MCMBP,ZC3H18,RBBP6,HNRNPA1,MEF2D,SPTBN1,CARMIL1,SVIL,HMGA1,HUWE1,ARHGAP29,LARP7,NUP153,PRRC2A,AFDN,MARCKS,ABI2,MYBBP1A,RAB12,KIF4A,CNOT2,ACIN1,EIF4G1
6	AURKB	39.09	TOP2A,HSP90AB1,RIF1,NUMA1,CHD4,MKI67,SMC3,EIF4ENIF1,RAVER1,KIF1C,BBX,KIF21A,KIF1B,NUP214,SMARCC1,MAP2K2,LMO7,KIF23,RAD23B,NCOR2,PSMA3,DYNC1LI2,NCOR1,CTTN,CANX,ARHGEF2,RAF1,SQSTM1,PLEC,ANKRD17,AHNAK,CDCA3,KMT2A,ASAP1,PDS5B,POLR2A,TPR,LMNA,RPS3,MCMBP,HNRNPA1,SPTBN1,SVIL,HMGA1,HUWE1,PRRC2C,NUP153,MYBBP1A,KIF4A,RBMX
7	KSR1	41.29	ANKRD17,HSP90AB1,PRAG1,ZBTB21,ATN1,CHD4,NDRG1,EDC4,RBM15B,SLK,MPRIP,LMNA,MCMBP,KIF1C,KIF1B,MEF2D,SPTBN1,RALGPS2,MED1,SMARCC1,MAP2K2,KIF23,NCOR2,ATXN2L,AFDN,ACLY,CTTN,ABI2,MAP7D1,RAB12,CANX,ARHGEF2,RAF1,SQSTM1,PHLDB2
8	AKT1	43.27	OGFR,TOP2A,LAD1,HSP90AB1,PRAG1,NUMA1,RIF1,ATN1,CHD4,CASC3,MKI67,SMC3,ROBO1,EDC4,SLK,MPRIP,KIF1C,KIF21A,KIF1B,SLC16A3,AGFG1,RALGPS2,NUP214,SRRM2,MED1,EIF5B,SMARCC1,MAP2K2,TPI1,SCAF11,AFAP1,KIF23,LMO7,RAD23B,UTP18,NCOR2,ATXN2L,ACLY,PSMA3,NCOR1,STIM1,CTTN,PALLD,TBC1D5,CANX,ARHGEF2,RAF1,S100A9,SQSTM1,PHLDB2,OPTN,PLEC,ANKRD17,AHNAK,KMT2A,ASAP1,PDS5B,NDRG1,KLC4,RBM15B,BCLAF1,HSPH1,TCOF1,POLR2A,CALD1,LMNA,TPR,RPS3,RBBP6,HNRNPA1,ZC3H13,MEF2D,SPTBN1,FAM83B,REPS1,SVIL,HMGA1,HUWE1,IRF2BP2,ARHGAP29,LARP7,NUP153,L1CAM,SLC4A7,AFDN,MARCKS,ABI2,MYBBP1A,KIF4A,CNOT2,RAB12,ACIN1,RBMX,EIF4G1
9	ABL1	45.45	TOP2A,HSP90AB1,NUMA1,RIF1,ATN1,CHD4,MKI67,SMC3,ROBO1,EDC4,GOLGA4,SLK,KIF1C,KIF1B,FAAP100,RALGPS2,NUP214,MED1,SMARCC1,MAP2K2,AFAP1,KIF23,LARP4B,RAD23B,ATXN2L,NCOR2,PSMA3,NCOR1,CTTN,STIM1,PALLD,TBC1D5,ARHGEF2,RAF1,SQSTM1,S100A9,PHLDB2,PLEC,ANKRD17,KMT2A,YTHDC1,ASAP1,PDS5B,KLC4,BCLAF1,POLR2A,TCOF1,KIAA1522,TPR,LMNA,RPS3,RBBP6,HNRNPA1,MEF2D,SPTBN1,REPS1,SVIL,HUWE1,NUP153,L1CAM,AFDN,MARCKS,ABI2,MYBBP1A,KIF4A,RAB12,ACIN1,EIF4G1

10	SRC	46	TOP2A,HSP90AB1,PRAG1,RIF1,ZDHHC5,ATN1,CHD4,MKI67,SMC3,ROBO1,EDC4,GOLGA4,SLK,MPRIP,RAVER1,TNKS1BP1,KIF1C,KIF1B,SLC16A3,RALGPS2,AGFG1,NUP214,MED1,EIF5B,SMARCC1,MAP2K2,SCAF11,AFAP1,ZFP91,LMO7,KIF23,LARP4B,RAD23B,ATXN2L,NCOR2,ACLY,PSMA3,NCOR1,CTTN,STIM1,THRAP3,PALLD,CANX,ARHGEF2,RAF1,PHLDB2,SQSTM1,S100A9,OPTN,PLEC,ANKRD17,AHNAK,KMT2A,YTHDC1,ASAP1,NDRG1,FAM83H,KLC4,BCLAF1,RBM15B,HSPH1,POLR2A,CALD1,LMNA,TPR,RPS3,RBBP6,HNRNPA1,MEF2D,SPTBN1,REPS1,CARMIL1,SVIL,HMGA1,HUWE1,IRF2BP2,ARHGAP29,NUP153,L1CAM,SLC4A7,AFDN,MARCKS,ABI2,MYBBP1A,RAB12,KIF4A,CNOT2,ACIN1,RBMX,EIF4G1
**Hyperphosphorylated proteins**			
1	CDK14	30	KLC4,PCYT1A,PDLIM2,CCNY,DLG5,CSNK2B
2	ABL1	32	KLC4,GOLGA4,PDLIM2,CCNY,DLG5,CSNK2B,NUP153,ROBO1
3	GSK3B	45.55	KLC4,GOLGA4,PDLIM2,PRAG1,CCNY,DLG5,CSNK2B,NUP153,NDRG1,ROBO1
4	MAPK1	47.18	KLC4,GOLGA4,PCYT1A,PDLIM2,CCNY,DLG5,CSNK2B,NUP153,NDRG1,ROBO1
5	MAPK14*	49.82	KLC4,GOLGA4,PCYT1A,PDLIM2,CCNY,CSNK2B,NUP153,ROBO1
